# Warm Spring Days are Related to Shorter Durations of Reproductive Phenophases for Understory Forest Herbs

**DOI:** 10.1002/ece3.70700

**Published:** 2024-12-16

**Authors:** Chelsea N. Miller, Katharine L. Stuble

**Affiliations:** ^1^ Holden Arboretum Kirtland Ohio USA; ^2^ The University of Akron Akron Ohio USA

**Keywords:** arboreta, botanical gardens, historical climate, phenology, spring ephemerals, temperature normals, wildflowers

## Abstract

As plants continue to respond to global warming with phenological shifts, our understanding of the importance of short‐lived heat events and seasonal weather cues has lagged relative to our understanding of plant responses to broad shifts in mean climate conditions. Here, we explore the importance of warmer‐than‐average days in driving shifts in phenophase duration for spring‐flowering woodland herbs across one growing season. We harnessed the combined power of community science and public gardens, engaging more than 30 volunteers to monitor shifts in phenology (documenting movement from one phenophase to the next) for 198 individual plants of 14 species twice per week for the 2023 growing season (March—October) across five botanic gardens in the midwestern and southeastern US. Gardens included the Holden Arboretum, Kirtland, OH; Dawes Arboretum, Newark, OH; Chicago Botanic Garden, Glencoe, IL; Missouri Botanical Garden, St. Louis, MO; and Huntsville Botanical Garden, Huntsville, AL. We tested: (1) that higher‐than‐average daily temperatures (deviation from 30‐year historical mean daily temperatures for each location) would be related to truncated phenophase durations; and (2) that phenophase durations would vary among species. Our findings support both hypotheses. We documented significant inverse relationships between positive deviations in daily temperature from historic means (e.g., warmer‐than‐average days) and durations of three reproductive phenophases: “First Bud,” “First Ripe Fruit,” and “Early Fruiting.” Similar (non‐significant) trends were noted for several other early‐season phenophases. Additionally, significant differences in mean phenophase durations were detected among the different species, although these differences were inconsistent across plant parts (vegetative, flowering, and fruiting). Results underscore the potential sensitivity of understory herb reproductive phenophases to warmer‐than‐average daily temperatures early in the growing season, contributing to our understanding of phenological responses to short‐term heat events and seasonal weather cues.

## Graphical abstract

The effect of warm daily temperatures on the phenology of 14 species of spring‐flowering woodland herbs was investigated across five US botanic gardens. By engaging community scientists to monitor plants twice per week, we found that high daily temperatures early in the year were significantly related to shorter durations of reproductive phenophases like “First Flower Bud” and “First Ripe Fruit,” along with species‐specific variations. These findings highlight the sensitivity of spring wildflowers to higher‐than‐average daily temperatures, with implications for future climate‐induced phenological shifts.

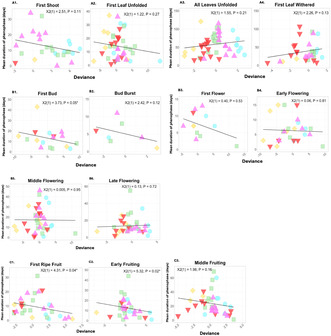

## 1 Introduction

Interest in the effects of warming temperatures on phenology—periodically recurring patterns of organismal growth and development (Lieth 1974)—has surged over the past several decades due to its importance as a driver of ecosystem structure and function (Piao et al. 2019). Responses of plant phenology to temperature are of particular concern because plants play critical roles as primary producers and serve as a foundation for many biotic interactions. In the Northern hemisphere, warming has caused plant phenology to shift earlier in the spring (Cleland et al. 2007; Fu et al. 2014; Allstadt et al. 2015) and, with less consistent evidence, later in the autumn (Gill et al. 2015; Liu et al. 2016; Yan et al. 2021). For example, climate change has been shown to advance spring phenophases anywhere from 1 to 6 days per degree of warming (Bertin 2008; Wolkovich et al. 2012). Long‐term observational datasets indicate that autumn senescence in temperate biomes is delayed by increasing temperatures, although characterization of shifts in autumnal phenology is challenged by the complexity of drivers and the protracted nature of autumn events as compared to spring (Gallinat, Primack, and Wagner 2015). Importantly, autumn senescence has been found to be correlated with the timing of spring budburst across the eastern United States, meaning the effects of climate change on autumn phenology are filtered through shifts in spring phenology (Keenan and Richardson 2015). Such findings highlight the importance of understanding not only shifts in the timing of early‐season phenology but also interphase durations, as these may have cascading effects on later phases throughout the year (Ettinger, Gee, and Wolkovich 2018).

Altered flowering phenology, specifically, can have dramatic negative effects on plant reproduction and fitness. For example, in their study of flowering and fruiting responses to experimental warming of angiosperms in a subalpine meadow, Price and Waser (1998) found that early snowmelt, tied to experimental warming, resulted in early flowering. The authors observed that late spring frosts often damaged flowers and buds of spring ephemerals such as *Erythronium grandiflorum* that had emerged earlier in warmed plots. Thus, temperature‐driven changes in flowering phenology may place subalpine spring ephemerals at greater risk of damage due to late‐spring frost events (Price and Waser 1998). Kudo and Ida (2013) demonstrated the deleterious effects of warming‐induced early flowering on plant–pollinator phenological mismatch for a spring ephemeral, *Corydalis ambigua*, in Hokkaido, Japan. Climate change‐induced shifts in flowering phenology can also lead to fitness tradeoffs for plants, which may find themselves caught between risks and benefits associated with shifts in timing. For instance, Gezon, Inouye, and Irwin (2016) found that, while individuals of the spring herb *Claytonia lanceolata* with experimentally advanced flowering times were able to reap the benefits of enhanced pollination services as a result of decreased competition, severe frost damage ultimately resulted in very low reproductive output for these individuals.

The ramifications of shifts in plant phenology often extend beyond the direct effects on plants themselves (Tang et al. 2016). For example, in the eastern United States, changes in deciduous forest phenology over several decades resulted in significant changes in ecosystem functions, including productivity and carbon cycling (Richardson et al. 2010; Keenan et al. 2014). Ecosystem‐level changes in plant phenology can then feedback with global climate conditions (Piao et al. 2019). As touched on above, shifts in plant phenology can significantly alter species interactions, including pollination (Kudo and Ida 2013; Forrest 2015; Gezon, Inouye, and Irwin 2016; Freimuth et al. 2022), herbivory (Fabina, Abbott, and Gilman 2010; Hamann et al. 2021), and species coexistence (Cousens, Barnett, and Barry 2003; Rudolph, 2019; Tiusanen et al. 2020). Such phenological mismatches can contribute to altered patterns of flowering within plant communities, a redistribution of floral abundance across growing seasons, and expansion of the flowering season itself, demonstrating that phenological shifts have substantial potential to reshape ecological communities with cascading effects (CaraDonna, Iler, and Inouye 2014; Pareja‐Bonilla et al. 2023). Additionally, temporal shifts specifically related to the duration of phenological stages—resulting in shorter or longer phenophases—can have compounding effects on plant fitness, species distributions (Ettinger et al. 2021), and ultimately community composition (Chen et al. 2020).

While research into temperature‐driven phenological shifts usually considers increases in temperature means (i.e., mean climatic conditions), the importance of temperature extremes (i.e., extreme climatic or weather events) as a driver of phenological shifts is less understood (Menzel, Seifert, and Estrella 2011; Reyer et al. 2013; Orsenigo et al. 2014; Vogel 2022). This, despite the fact that the frequency, intensity, geographic scale, and location of extreme weather and climatic events have been, and will continue to be, altered by climate change (Horton, Folland, and Parker 2001; Trenberth et al. 2007; Luber and McGeehin 2008; Rummukainen 2012; Orsenigo et al. 2014; Ummenhofer and Meehl 2017). Extreme climatic events can include warm and cold spells, heat waves, drought, advanced or delayed snowmelt, frosts, heavy rainfalls, pulsed rainfalls, and flooding (Menzel, Seifert, and Estrella 2011; Orsenigo et al. 2014). It is important to note that perspectives on extreme weather and climate events vary broadly, depending on whether or not they result in extreme impacts to natural systems (Parry et al., 2007; Menzel, Seifert, and Estrella 2011). Further complicating efforts to investigate the effects of extreme weather and climate events on biotic systems is the lack of a consistent definition for “extreme” events, especially among disciplines (physical systems vs. natural sciences; Menzel, Seifert, and Estrella 2011). For example, extreme temperatures may be defined on the basis of tails of distributions (Beniston 2009), by exceeding absolute thresholds (e.g., identification of anomalous warm spells persisting for several days compared to area‐averaged surface temperatures; Satyamurty, da Silva Teixeira, and Klug Padilha 2007), by measures of variation (e.g., seasonally varying thresholds as opposed to fixed temperature thresholds; Jones et al. 1999), by deriving percentiles from fits of climate anomalies (e.g., Horton, Folland, and Parker 2001), or by the application of statistical analyses of extreme values under nonstationarity (e.g., Katz 2010).

Despite these challenges, studies have generally found that short‐lived extreme climatic and weather events have substantial impacts on plant phenology, particularly if they occur during sensitive life stages (Menzel, Seifert, and Estrella 2011; Reyer et al. 2013; Friedl et al. 2014; Vogel 2022). For instance, severe drought (32 days) and heavy rainfall (170 mm over 14 days) caused phenological shifts in grassland plants of Central Europe of the same magnitude as one decade of gradual warming (advancement of mid‐flowering date by 4 days [averaged over all species] and reduction in flowering length by 4.35 days [averaged over all species], respectively; Jentsch et al. 2009). Such extreme (although often short‐term) weather conditions also have the potential to impact the progression of seasonally dependent phenological transitions in plants through increased rates of development (Badeck et al. 2004). These transitions are driven by a combination of phenotypic plasticity and adaptive evolution (Anderson et al. 2012). Taken together, these findings demonstrate the importance of characterizing the effects of fine temporal‐scale temperature increases on phenology—and specifically phenophase duration—particularly as such weather patterns become more common as the climate continues to warm.

While many studies have characterized phenological responses of trees to variation in temperature (Gill et al. 2015; Primack et al. 2015; Piao et al. 2019), less research has focused on understory forest herbs, including spring ephemeral species. Spring ephemerals are among the most vulnerable plants to the deleterious effects of warming‐induced shifts in phenology, given their short phenological window early in the growing season for completion of their aboveground lifecycle (Heberling et al. 2019). Spring ephemerals and other early spring‐flowering herbs are also at increased risk of phenological mismatch with mutualistic partners critical to reproductive success. For example, Kudo and Cooper (2019) found that early snowmelt increased the risk of phenological mismatch under natural conditions between a spring ephemeral (*Corydalis ambigua*) and its pollinator (overwintered bumblebees) in a deciduous forest of northern Japan. This is particularly concerning given that spring ephemerals rely heavily on animal pollinators and seed dispersers (Kudo and Ida 2013; Rafferty, CaraDonna, and Bronstein 2015; Ziemianski and Zych 2016; Kehrberger and Holzschuh 2019; Tsuzuki and Ohara 2022). Furthermore, Schieber (2007) demonstrated via a multiyear observational comparative experiment in central Slovakia that spring‐flowering herbs flowered earlier, experienced shorter interphase interval durations, and exhibited greater variability in both the timing and duration of phenophases than did summer‐flowering herbs, suggesting that spring‐flowering herbaceous species may be at greater risk of warming‐induced shifts in both the timing and duration of critical reproductive phenophases than other herbaceous species. Thus, understanding how changes in both mean climate and short‐term climatic and weather variability impact the overall phenology and phenophase duration of these species is a conservation priority.

It can be difficult to collect phenological data at scales required to document phenological shifts, especially in the context of temporally fine‐scale climatic variations. When scaling across large geographic regions, this can become nearly impossible for individual researchers, or even teams of researchers. Community science (formerly “citizen” science) offers a powerful system to support the collection of data needed to synthesize patterns of phenology across both fine temporal and broad geographic scales (Fuccillo et al. 2015; Gallinat et al. 2021). New user‐friendly tools are beginning to facilitate the collection of phenological data by community scientists (e.g., iNaturalist 2024; Budburst 2024). By bringing together diverse suites of plants into a common space where they are readily accessible to community scientists, botanical gardens and arboreta offer a particularly powerful platform in which community scientists can collect plant phenology data. Public gardens enable people to easily collect data across multiple species in relatively short amounts of time, while simultaneously taking the guesswork out of plant identification, making data collection widely accessible.

Here, we leverage the strengths of a public garden‐based community science approach to link the duration of phenophases in spring wildflowers to deviations in mean daily temperatures from historic normals across the landscape (e.g., Yang and Rudolf 2010). At five public gardens across the midwestern and southeastern United States, community scientists documented twice‐weekly plant phenology data for the 2023 growing season (March—October) for a suite of forest spring wildflowers (14 species). We hypothesized that higher‐than‐average daily temperatures (measured as deviance in mean daily 2023 temperatures from 30‐year historic daily temperatures for each garden location) would be related to shorter mean durations of spring wildflower phenophases. That is to say, we expected that unseasonably warm temperatures would accelerate the advancement of plant phenology from one phenophase to the next at a faster rate. We also tested whether there were differences in the mean duration of vegetative, flowering, and fruiting phenophases among species. Our results establish that short periods of higher‐than‐average temperatures can result in rapid sequential shifts in understory herb phenophases, particularly early in the growing season. Our study also indicates that different aspects of plant phenology (i.e., vegetative, flowering, and fruiting) vary in their relative sensitivity to higher‐than‐average daily temperatures.

## 2 Materials and Methods

### 2.1 Study Species

Fourteen spring ephemeral wildflower species were used in this study: *Arisaema triphyllum* L. (Schott) (jack‐in‐the‐pulpit), *Asarum canadense* L. (wild ginger), *Claytonia virginica* L. (spring beauty), *Erythronium americanum* Ker‐Gawl. (yellow trout lily), *Erythronium albidum* L. (white trout lily), *Jeffersonia diphylla* L. (Pers.) (twinleaf), *Maianthemum racemosum* L. (Link) (false Solomon's seal), *Podophyllum peltatum* L. (mayapple), *Polygonatum biflorum* (Walt.) Ell. (smooth Solomon's seal), *Sanguinaria canadensis* L. (bloodroot), *Stylophorum diphyllum* (Michx.) Nutt. (celandine poppy), *Trillium erectum* L. (red trillium), *Trillium grandiflorum* (Michx.) Salisb. (great white trillium), and *Trillium recurvatum* L. C. Beck. (prairie trillium) (Table 1). All study species are perennial understory herbs native to eastern North American deciduous forests. Although the term “spring ephemeral” is loosely defined (but see Yancy et al. 2023), all of these species adhere to the life history strategy of emerging early in the spring and completing all or most of their reproductive life cycle prior to closure of the forest canopy in early summer. Some of the species, including the two *Erythronium* species and *Claytonia virginica*, are “true” spring ephemerals in the sense that they emerge, complete their entire aboveground lifecycle, and senesce before the forest canopy closes. The remainder of the species are spring‐flowering herbs that persist aboveground at various phenological stages into the summer growing season.

**TABLE 1:** List of study species (Latin binomial and common name) observed during the 2023 growing season at each botanical garden (CBG = Chicago Botanic Garden; Holden = The Holden Arboretum; Dawes = The Dawes Arboretum; Mobot = The Missouri Botanical Garden; Huntsville = Huntsville Botanical Garden). Each cell value represents the number of individual plants of the corresponding species in the respective garden. Total number of plants observed in each garden is listed in the bottom row.

Species	Common name	CBG	Holden	Dawes	Mobot	Huntsville
*Arisaema triphyllum*	Jack‐in‐the‐pulpit	3	6	2	4	3
*Asarum canadense*	Wild ginger	4	5	4	4	3
*Claytonia virginica*	Spring beauty	3	0	5	3	6
*Erythronium albidum*	White trout lily	0	0	2	0	0
*Erythronium americanum*	Yellow trout lily	0	5	0	1	5
*Jeffersonia diphylla*	Twinleaf	1	5	0	1	5
*Maianthemum racemosum*	False Solomon's seal	3	5	1	5	1
*Podophyllum peltatum*	Mayapple	4	5	6	4	5
*Polygonatum biflorum*	Smooth Solomon's seal	0	2	1	6	4
*Sanguinaria canadensis*	Bloodroot	1	5	1	4	4
*Stylophorum diphyllum*	Celandine poppy	0	6	5	5	2
*Trillium erectum*	Red trillium	0	4	1	0	1
*Trillium grandiflorum*	Great white trillium	2	5	5	3	5
*Trillium recurvatum*	Prairie trillium	2	1	1	5	3
	Totals	23	49	34	45	47

### 2.2 Study Sites

**FIGURE 1:**
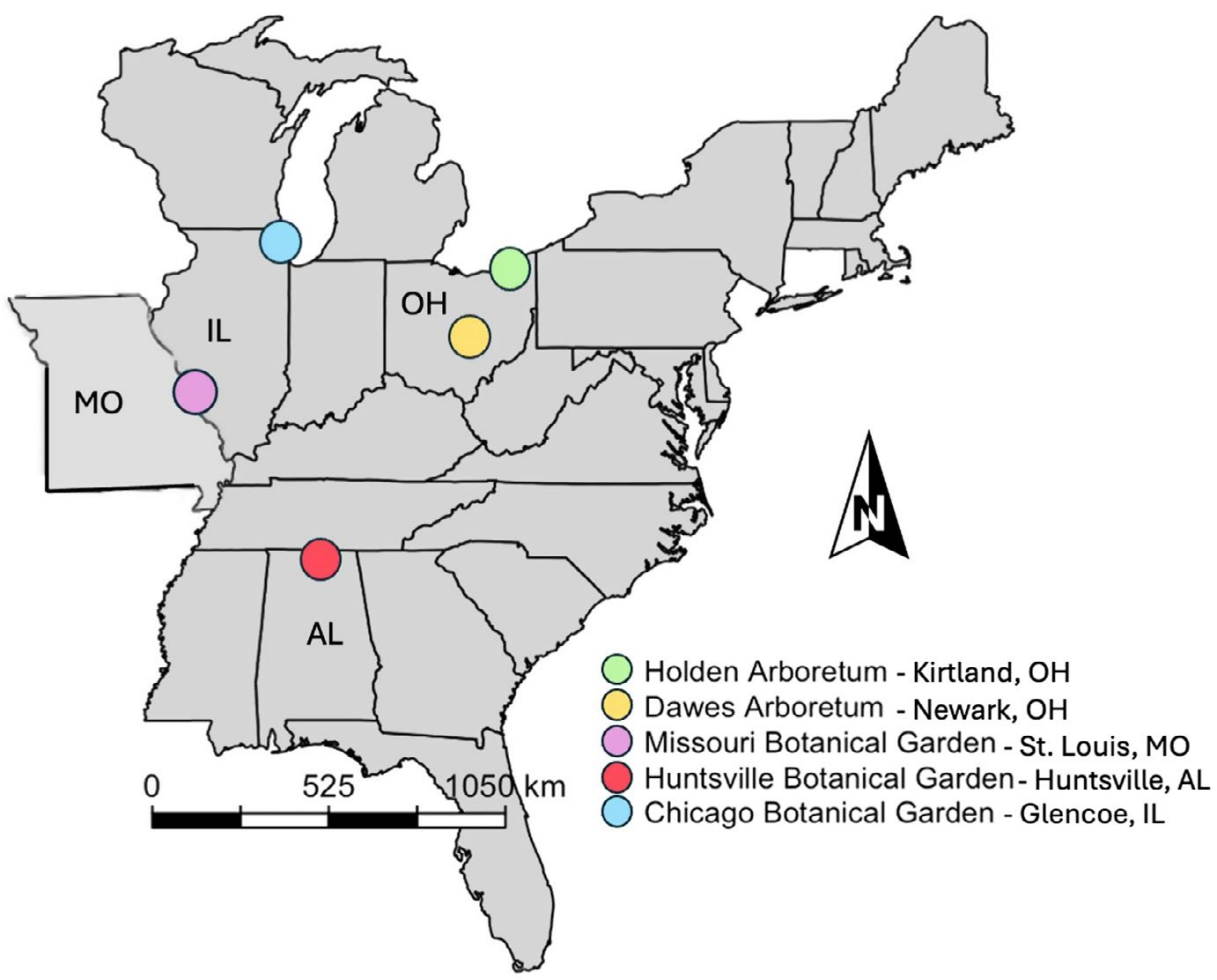
Botanical gardens and arboreta used as study sites (garden identity indicated by colored dots). Map depicts the midwest and southeastern United States. States in which the gardens are located are identified on the map.

We observed individuals of each study species growing within the collections of one or more public gardens in the midwestern and southeastern United States. Gardens included the Chicago Botanic Garden (CBG) in Glencoe, IL; Holden Arboretum in Kirtland, OH; Dawes Arboretum in Newark, OH; Missouri Botanical Garden in St. Louis, MO; and Huntsville Botanical Garden in Huntsville, AL (Figure 1). These gardens were chosen because they contained accessioned individuals of most of the study species ( *T. erectum* and *E. albidum* were only present in two of the gardens, but all other species were present in at least three gardens) and were easily accessible to members of the public.

### 2.3 Phenology Observations

In February 2023, the first author traveled to each garden and selected plants for inclusion in the study with the assistance of garden employees familiar with the collections and in consultation with garden records. A total of 198 individual plants of 14 species were selected for observation. These included 23 plants at CBG; 49 at Holden Arboretum; 34 at Dawes Arboretum; 45 at Missouri Botanical Garden; and 47 at Huntsville Botanical Garden. Table 1 details the number of individuals of each species that were observed at each garden. Community volunteers consistently observed a specific set of assigned plants throughout the growing season (i.e., each volunteer visited each individual plant assigned to them multiple times, and volunteers did not swap plants throughout the course of the study). Between six and nine volunteers collected data at each garden, for a total of 31 volunteers (CBG = 9 volunteers; The Holden Arboretum = 9 volunteers; Missouri Botanical Garden = 7 volunteers; and Huntsville Botanical Garden = 6 volunteers). At The Dawes Arboretum, a team of two to three garden interns conducted the phenology observations.

Plants were each observed twice per week (observation events were separated by at least 1 day) from March 1 to October 31, 2023 (36 weeks), for a total of 14,256 observation events (approximately 72 observation events per individual plant). On rare occasions, an observation event would be missed due to logistical constraints, such as inclement weather or scheduling conflicts; but in general, the schedule outlined above was carefully adhered to. Species identities of all observed plants were provided to the volunteers (e.g., we did not rely on volunteers to confirm the identities of the plants), and garden horticultural staff familiar with each species assisted volunteers in identifying study specimens as they first emerged.

**FIGURE 2:**
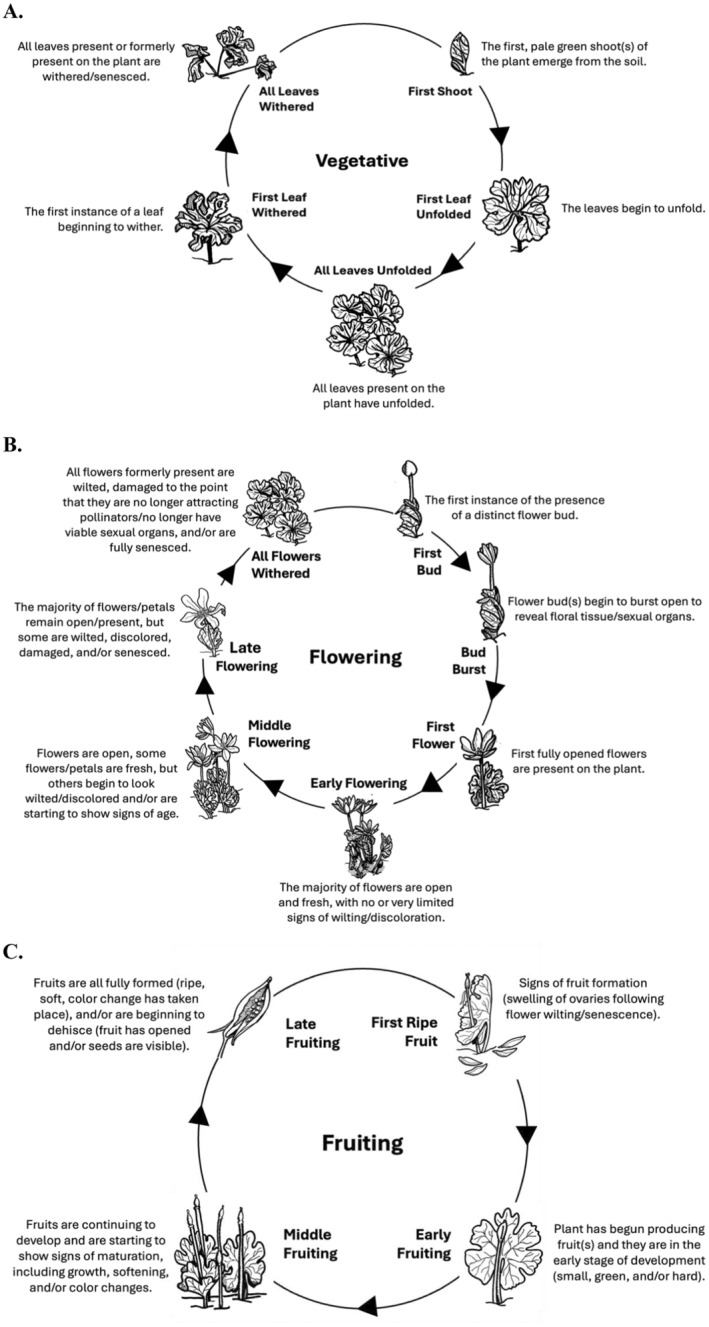
Phenology wheels for plant parts (A. vegetative; B. flowering; C. fruiting) and individual, sequential phenophases, as recorded by observers in the Budburst app. (A) Vegetative phenology consists of five phenophases (first shoot, first leaf unfolded, all leaves unfolded, first leaf withered, and all leaves withered). (B) Flowering phenology consists of seven phenophases (first bud, bud burst, first flower, early flowering, middle flowering, late flowering, and all flowers withered). (C) Fruiting phenology consisted of four phenophases (first ripe fruit, early fruiting, and late fruiting). In total, there were 16 distinct phenophases across vegetative, flowering, and fruiting phenologies for volunteers to select from during each observation event (Figure 2). Wheels modified from Budburst (Budburst 2024); drawings illustrate phenophases for bloodroot ( *Sanguinaria canadensis* ).

During a phenology observation event, a volunteer would record the: (1) vegetative, (2) flowering, and (3) fruiting phenology stage of a target plant. Phenophases (e.g., distinct stages of phenology; Figure 2) were recorded using the web and smartphone app “Budburst” (Budburst 2024). Budburst is a free and publicly available app that allows members of the general public to record the phenological stage of any plant using an interactive infographic called a “phenology wheel” (Figure 2). Excluding the “None” option, vegetative phenology consisted of five phenophases: “First Shoot” (the first, pale green shoot(s) of the plant emerged from the soil), “First Leaf Unfolded” (the leaves began to unfold), “All Leaves Unfolded” (all leaves present on the plant had unfolded), “First Leaf Withered” (the first instance of a leaf that began to wither), “All Leaves Withered” (all leaves present or formerly present on the plant were withered/senesced; Figure 2A). Flowering phenology consisted of seven phenophases: “First Bud” (the first instance of the presence of a distinct flower bud), “Bud Burst” (flower bud(s) began to burst open to reveal floral tissue/sexual organs), “First Flower” (first fully opened flowers were present on the plant), “Early Flowering” (the majority of flowers were open and fresh, with no or very limited signs of wilting/discoloration), “Middle Flowering” (flowers were open, some flowers/petals were fresh, but others began to look wilted/discolored and/or were starting to show signs of age), “Late Flowering” (the majority of flowers/petals remained open/present, but some were wilted, discolored, damaged, and/or senesced), and “All Flowers Withered” (all flowers formerly present were wilted, damaged to the point that they were no longer attracting pollinators/no longer had viable sexual organs, and/or were fully senesced; Figure 2B). Fruiting phenology consisted of four phenophases: “First Ripe Fruit” (signs of fruits formation [swelling of ovaries following flower wilting/senescence]), “Early Fruiting” (the plant had begun producing fruit in the early stage of development [small, green, and/or hard]), “Middle Fruiting” (fruits were continuing to develop and were starting to show signs of maturation, including growth, softening, and/or color changes), and “Late Fruiting” (fruits were all fully formed [ripe, soft, color change had taken place] and/or were beginning to dehisce [fruit had opened and/or seeds were visible]; Figure 2C). In total, there were 16 distinct phenophases across vegetative, flowering, and fruiting phenologies for volunteers to select from during each observation event. We used this standard set of phenophases rather than the aggregated categories of vegetative, flowering, and fruiting because the longer time periods that these latter, coarser groupings span would not allow us to effectively explore how fine temporal scale variability in temperature impacts phenophase duration.

After initial observation, and using the Budburst app on a mobile device, a volunteer would record data by first uploading an image of the study plant in real time while recording the plant's scientific name and location (i.e., the garden in which they were located). Next, using the phenology wheels, the volunteer would select the specific stage of phenology (as described above) for each of the three phenophase categories (vegetative, flowering, and fruiting). The volunteer would have the option of uploading an image pertaining to each phenophase, which enabled us to verify the accuracy of volunteer observations. If there was something particularly notable about the observation, the volunteer would have the option of entering a note. Each phenology observation event took approximately 2–5 min such that each plant was observed for approximately 4–10 min per week and 144–360 min throughout the duration of the study.

At the end of the 2023 growing season, once all observation events were complete, we downloaded all recorded data affiliated with the project from Budburst.org as a single .csv file. Following data cleaning (in which we removed partial and/or inaccurate data entries and sorted data by garden, date, species, and individual plant), we calculated the mean duration of each distinct phenophase for each species in each garden. We accomplished this by first calculating the mean earliest date the phenophase was recorded across the individuals of each species at each garden (ranging from 1 to 6 individuals per garden), and then subtracting this value from the mean earliest date of the next sequential phenophase. For example, to calculate the mean duration of the “First Flower” phenophase for *Trillium grandiflorum* at the CBG, the mean earliest date that the “First Flower” phenophase was recorded for all individual *T. grandiflorum* plants at CBG was subtracted from the mean earliest date that the “Early Flowering” phenophase was recorded for all individual *T. grandiflorum* plants at CBG. In cases where there was only one individual representing the species at a site (*Erythronium americium* at the Missouri Botanical Garden; *Jeffersonia diphylla* at the CBG and the Missouri Botanical Garden; *Maianthemum racemosum* at the Dawes Arboretum and Huntsville Botanical Garden; *Polygonatum biflorum* at the Dawes Arboretum; *Trillium erectum* at the Dawes Arboretum and Huntsville Botanical Garden; and *Trillium recurvatum* at the Holden Arboretum and the Dawes Arboretum; Table 2), we used the absolute (rather than mean) dates to calculate durations. Means were calculated per species per garden for visualization purposes only; statistical analyses were performed on pooled species (*N* = 14 species) and garden (*N* = 5 gardens) mean phenophase durations to increase statistical power and to detect general trends for the entire suite of plants. This meant that the sample size per phenophase depended on the number of unique observations of that phenophase for the entire suite of species across all gardens, ranging from *n* = 7 (for bud burst) to *n* = 45 (for all leaves unfolded; see *Statistical analyses* below).

**TABLE 2:** Results of negative binomial generalized linear models (GLMs) testing whether the mean duration of vegetative, flowering, and fruiting phenophases across the suite of species at all gardens were related to the magnitude of differences between 2023 mean daily temperatures and historic daily mean temperatures (30‐year normals). Significance at *a* = 0.05 of covariate estimates and overall models are indicated with asterisks.

Plant part	Mean duration of phenophase	GLM				Significance of overall model	
		Coeff. est.	SE	*z*	*p*	*X* ^2^	*p*
Vegetative	First Shoot	−0.03875	0.02310	−1.678	0.09	2.51	0.11
	First Leaf Unfolded	−0.02470	0.01864	−1.325	0.19	1.22	0.27
	All Leaves Unfolded	0.04428	0.03110	1.424	0.15	1.55	0.21
	First Leaf Withered	0.08793	0.04847	1.814	0.07	2.26	0.13
Flowers	First Bud	−0.05645	0.02818	−2.003	0.04*	3.73	0.05*
	Bud Burst	−0.08468	0.05633	−1.503	0.13	2.42	0.12
	First Flower	−0.01641	0.02196	−0.748	0.46	0.40	0.53
	Early Flowering	−0.004462	0.016802	−0.266	0.80	0.06	0.81
	Middle Flowering	−0.001877	0.021687	−0.087	0.93	0.005	0.95
	Late Flowering	0.01099	0.02623	0.419	0.68	0.13	0.72
Fruits	First Ripe Fruit	−0.07922	0.03148	−2.516	0.01*	4.31	0.04*
	Early Fruiting	−0.07599	0.02519	−3.017	0.003*	5.32	0.02*
	Middle Fruiting	−0.05320	0.03051	−1.744	0.08	1.98	0.16

### 2.4 Temperature Variables

**FIGURE 3:**
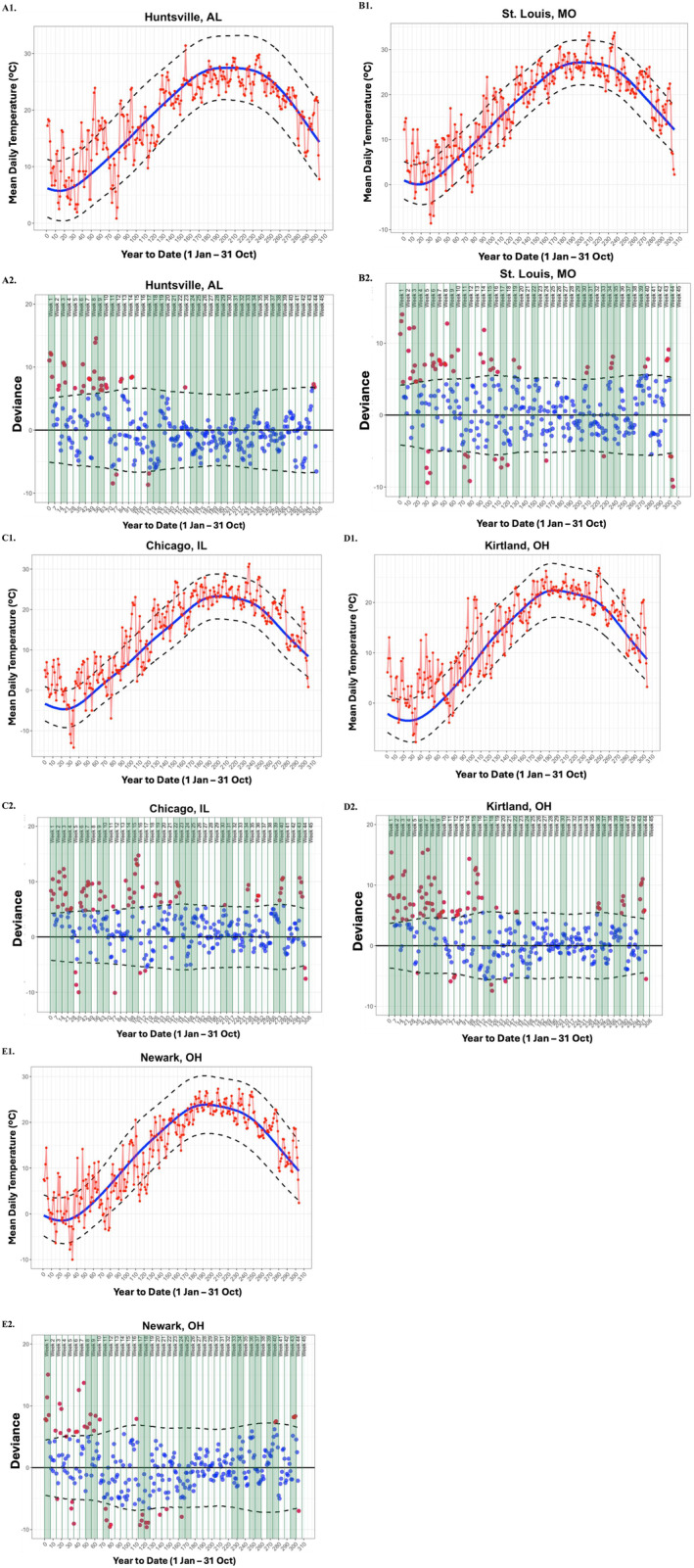
*Upper plots*. Red time series indicates mean daily temperature (°C) of A1. Huntsville, AL (Huntsville Botanical Garden), B1. St. Louis, MO (Missouri Botanical Garden), C1. Chicago, IL (Chicago Botanic Garden), D1. Kirtland, OH (Holden Arboretum), and E1. Newark, OH (Dawes Arboretum) from Jan. 1 to Oct. 31, 2023. The solid blue line indicates the 30‐year historic mean daily temperature (°C) averaged across 1991–2020 for each location. Black dashed lines indicate 30‐year historic mean daily maximum and minimum temperatures (°C) for each location. *Lower plots*. Dots indicate the deviance of mean daily temperature values ((2023 mean daily temperature [°C]) – (1991–2020 daily normals [°C])) for A2. Huntsville, AL (Huntsville Botanical Garden), B2. St. Louis, MO (Missouri Botanical Garden), C2. Chicago, IL (Chicago Botanic Garden), D2. Kirtland, OH (Holden Arboretum), and E2. Newark, OH (Dawes Arboretum) from Jan. 1 to Oct. 31, 2023 around the 30‐year historic daily mean (solid black line, set to 0) for each location. Dashed lines indicate the range of 30‐year historic mean daily maximum and minimum temperature values surrounding the 30‐year mean daily temperature for each location. Blue dots indicate days in 2023 that fell within the historical maximum–minimum temperature range; red dots indicate days in 2023 that fell outside of the historical maximum–minimum temperature range. Solid vertical green bars indicate the year (2023)‐ broken into Weeks 1–44; green shaded areas indicate weeks with 2023 mean daily temperatures that deviated significantly (*p* < 0.05) from the 30‐year historic mean (see Supporting Information Table 1).

Thirty‐year historic mean daily temperatures (°C; averaged across 1991–2020) for each of the cities in which the gardens were located (Glencoe, IL, USA; Kirtland, OH, USA; Newark, OH, USA; St. Louis, MO, USA; Huntsville, AL, USA) were retrieved from the NOAA National Centers for Environmental Information website (NOAA 2023; Figure S1). To characterize temperature conditions in the study year, we obtained mean daily temperatures (°C) from January 1 to October 31, 2023, for each location from Weather Underground (Weather Underground 1995; Figure S2). We then calculated the deviance (i.e., difference) between 2023 mean daily temperatures and the 30‐year historic daily mean temperatures for every day between 1 January and 31 October at each of the five locations (Figure 3). The use of city‐level data allowed us to pair the 2023 data with historic temperature data in ways that were not possible at the garden level.

### 2.5 Statistical Analyses

All statistical analyses were performed using the statistical software RStudio (version 2023.06.0 + 421; Posit Team 2023). To quantitatively compare the deviations in mean daily temperatures in 2023 with historical temperature conditions at each of the five gardens, we used one‐sample *t*‐tests to determine whether 2023 mean daily temperatures for each garden were significantly different from the 30‐year historic daily mean temperatures for each week of the year. To do so, we used the deviation in 2023 daily mean temperature from the 30‐year historic daily mean temperature for each garden as calculated above. Data were grouped by garden and day of year (DOY), and then temperatures were averaged over stretches of 7 consecutive days to yield an average deviance in mean daily temperature per week per garden, from January 1, 2023, to October 31, 2023. Next, we applied one‐sample *t*‐tests to the deviations for each week and each garden, testing the null hypothesis of zero deviation from the 30‐year historic daily means. Results, including test statistics and *p*‐values, are included in Table S1.

To test whether larger deviations (positive and negative) between 2023 mean daily temperatures and 30‐year historic daily means related to shorter durations of understory herb phenophases, we ran independent generalized linear models (GLMs) for each phenophase (excluding final vegetative, flowering, and fruiting phenophases, as we were unable to calculate phenophase duration for these final phenophases), for a total of 13 models. Generalized linear models were selected for use because of their flexibility in handling nonlinear (i.e., nonnormal and noncontinuous) data. Response variables were mean duration (in days) of the phenophase for each species in each garden. For each model, we initially considered two possible error structures (Poisson and negative binomial) appropriate for modeling the response variables, which are skewed, discrete count data bounded on the lower side at 1. Neither Poisson nor negative binomial models are inherently linear tests; one major difference between a Gaussian linear regression (e.g., Normal GLM) and a Poisson or negative binomial GLM is that Gaussian linear regression uses ordinary least squares to minimize the residual sum of squares, whereas Poisson/negative binomial GLMs use maximum‐likelihood estimation (Zuur et al. 2009). Both Poisson and negative binomial models would appropriately accommodate the nonlinearity observed in the relationships between the explanatory and response variables in our dataset. To select between a Poisson and negative binomial error structure for each of our models, we determined model fit using Akaike's information criterion (AIC) scores. For all models, the negative binomial error structure was the best fit. This was also confirmed by checking that the residual deviance of the negative binomial models was of the same magnitude as the degrees of freedom, which indicates that the negative binomial model accounts appropriately for overdispersion in the count data (e.g., that it does not underestimate the variance, as is often the case with Poisson GLM; Warton et al. 2016).

The explanatory variable in each negative binomial GLM was the deviance (i.e., difference) in mean daily temperature in 2023 and the 30‐year historic mean daily temperature (1991–2020) during the time period in which the phenophase took place. Variable significance was assessed using Wald χ^2^ tests (function Anova in package car; Fox and Weisberg 2019) at α = 0.05. Mean durations of phenophases were pooled across species and across gardens within each model. We did not include interactions between individual species and/or gardens because there were not enough degrees of freedom to disentangle the large number of levels contained within each explanatory variable and combination of variables. However, our method of pooling the mean durations across species and gardens allowed us to capture overall trends while maintaining statistical power.

To test if there were differences in the mean duration of vegetative, flowering, and fruiting phenophases among spring wildflower species (independent of garden location), we ran separate GLMs for each phenophase, for a second set of 13 models (26 total GLMs in the study). As above, response variables were mean duration (in days) of the phenophase for each species at each garden—but in this case, the explanatory variables were the identity of the wildflower species (a categorical variable containing 14 levels). As above, for each GLM, we used AIC scores to determine which of two data distributions (Poisson or negative binomial) was a better fit for the response variable, and models with the lower AIC score were reported. In addition to AIC scores, we considered the dispersion of the models as above. Models with residual deviance scores that were of the same order of magnitude as the degrees of freedom were determined to be the best‐fit models and were reported (these always coincided with the lowest AIC score). Again, variable significance was assessed using Wald χ^2^ tests at α = 0.05. To assess pairwise differences in mean phenophase durations among wildflower species, Tukey post hoc tests were performed using the glht function in the R package multcomp (Hothorn, Bretz, and Westfall 2008).

## 3 Results

Results of the one‐sample *t*‐tests established that there were numerous instances in which 2023 weekly mean temperatures were significantly deviant (both higher and lower) than the 30‐year historic norms for all five gardens (Figure 3; Table S1). Temperature extremes across all gardens were typically characterized by conditions warmer than the 30‐year historic norms, with significantly cooler temperatures occurring less frequently and most often between mid‐June and late‐July (Tables S1 and S2). For example, at the Holden Arboretum in Kirtland, OH, there were 19 total weeks in 2023 that were either significantly warmer (13 weeks) or significantly cooler (6 weeks) than the baseline established by the 30‐year historical data. The bulk of these warmer weeks (9 weeks) occurred before April 15 (Figure 3D). The Huntsville Botanical Garden in Huntsville, AL, on the other hand, experienced 20 weeks in 2023 that were significantly deviant from the baseline (7 warmer and 13 cooler), and these significantly deviant weeks were more evenly spread throughout the growing season (Figure 3A).

### 3.1 Hypothesis 1

**FIGURE 4:**
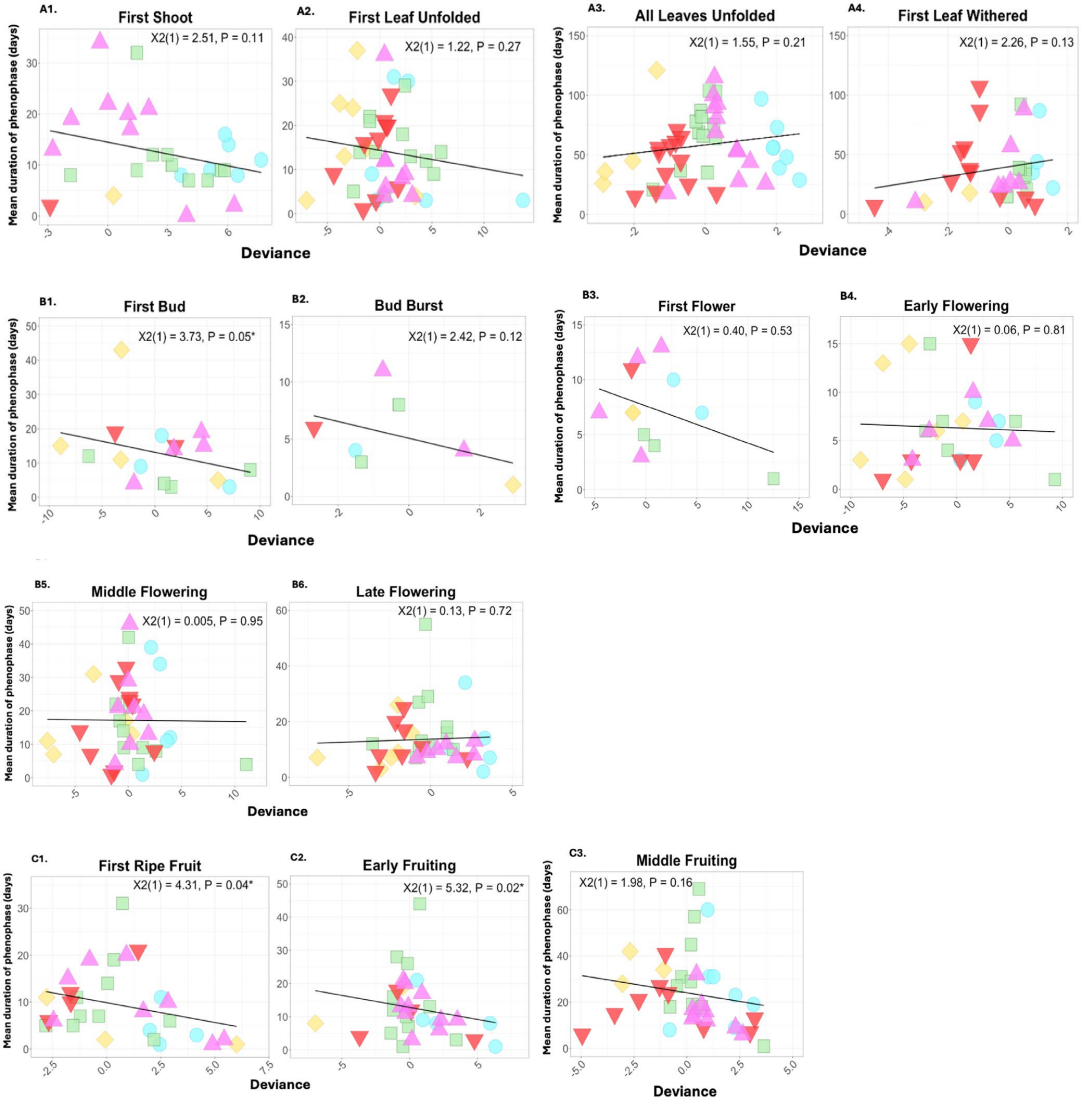
Scatterplots depicting the relationships between mean duration of each phenophase (A1–A4. Vegetative; B1–B6. Flowering; C1–C3. Fruiting) in days and the deviance of mean daily temperature values ((2023 mean daily temperature [°C]) – (1991–2020 daily normals [°C])) for each duration. Phenophase durations and temperature deviations are averaged across individuals of each species in each garden. Point colors and shapes correspond to the garden location (red triangles = Huntsville Botanical Garden; pink triangles = Missouri Botanical Garden; yellow diamonds = The Dawes Arboretum; green squares = The Holden Arboretum; blue circles = Chicago Botanic Garden). Linear regression trendlines (*for illustrative purposes only*) depict linear relationships between mean phenophase durations and mean differences in daily temperature from normals. Results of GLMs (either Poisson or negative binomial; Table 2) are included at the top of each plot and significance at *a* = 0.05 is indicated with an asterisk. Not depicted is the identity of the individual plant species in the dataset.

Mean durations of one of the flowering phenophases, “First Bud” (χ^2^(1) = 3.73, *p* = 0.05; overall mean = 12.39 ± 2.23 days), and two of the fruiting phenophases, “First Ripe Fruit” (χ^2^(1) = 4.31, *p* = 0.04; overall mean = 9.31 ± 1.36 days) and “Early Fruiting” (χ^2^(1) = 5.32, *p* = 0.02; overall mean = 12.09 ± 1.60 days), were significantly, inversely related to higher deviation in mean 2023 daily temperature from historic norms (Figure 4; Table 2). The overall mean deviation in daily temperature from historic norms during “First Bud” across all species and all gardens was 0.67°C ± 0.71°C; for “First Ripe Fruit” was 0.66°C ± 0.47°C; and for “Early Fruiting” was 0.72°C ± 0.50°C. These results suggest that positive deviance in mean daily 2023 temperatures from the 30‐year historic mean daily temperatures (e.g., conditions significantly warmer than average) resulted in significantly shorter durations of three early‐season, reproductive phenophases.

While none of the other models were significant at α = 0.05, several of the early‐season phenophases demonstrated similar inverse relationships between differences in mean daily temperature for 2023 from historic temperatures and phenophase durations. As the season progressed from spring to summer, many of the trends either flattened out (e.g., no relationship between distance from historic daily temperatures and phenophase duration) or even reversed (e.g., a positive relationship between deviance from historic daily temperatures and phenophase duration). This is especially evident for the last two vegetative phenophases (“All Leaves Unfolded” and “First Leaf Withered”) and the flowering phenophase (“Late Flowering”).

### 3.2 Hypothesis 2

**FIGURE 5:**
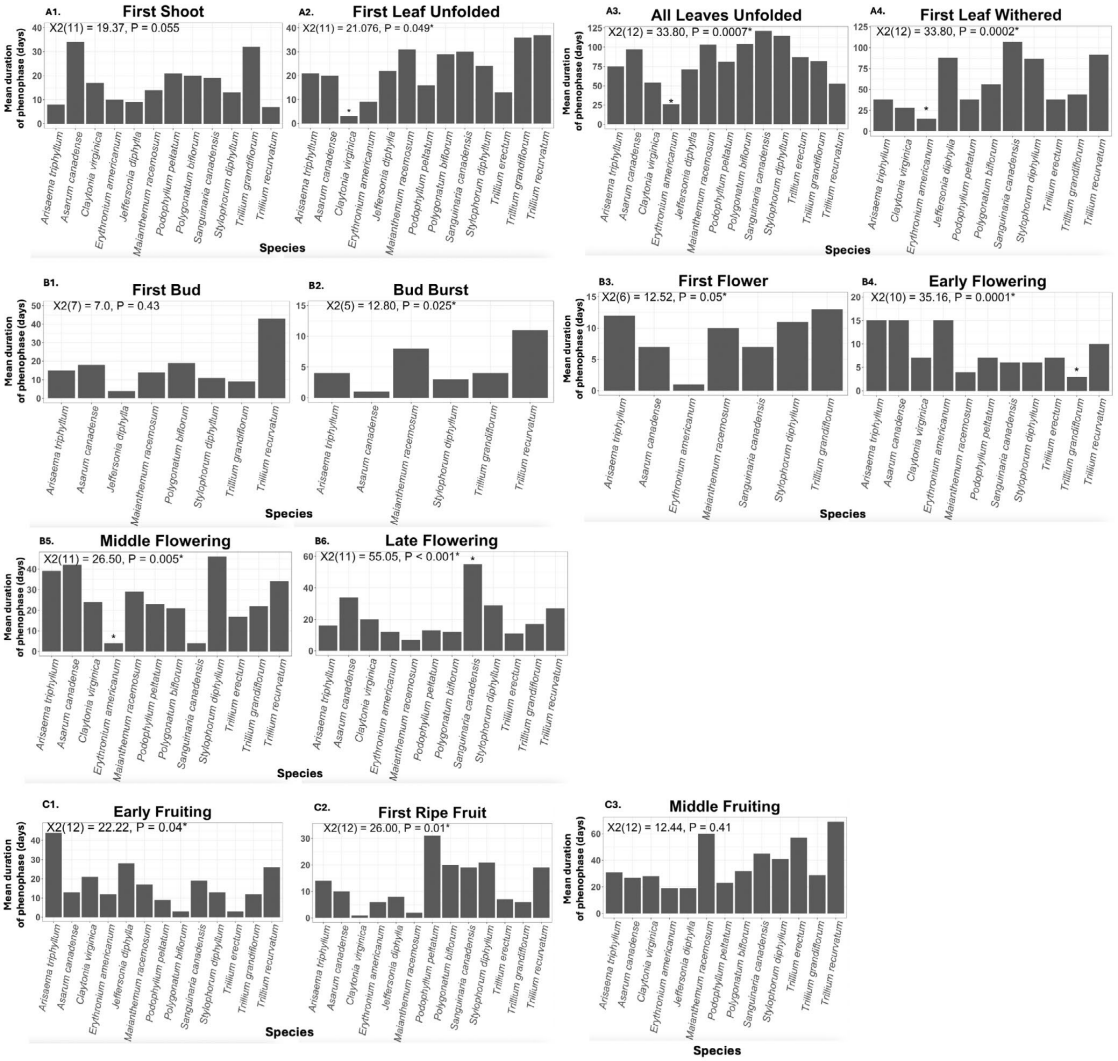
Barplots depicting mean duration of A1–A4: Vegetative; B1–B6: Flowering; and C1–C4: Fruiting phenophases in days for the plant species for which each phenophase was recorded across all gardens. Results of GLMs (either Poisson or negative binomial; Table 3) are included in each plot and significance at *a* = 0.05 is indicated with an asterisk. Tukey post hoc tests were performed for significant models, and species with significantly different mean phenophase durations are indicated with an asterisk above the bar.

Mean durations of 10 of the 13 phenophases were significantly different among at least 1 of the 14 spring wildflower species, although no particularly consistent trends emerged across the suite of phenophases (Figure 5; Table 3). However, when considering vegetative phenophases, for the three significant models (“First Leaf Unfolded”: χ^2^(11) = 21.076, *p* = 0.049; “All Leaves Unfolded”: χ^2^(12) = 33.80, *p* = 0.0007, “First Leaf Withered”: χ^2^(12) = 33.80, *p* = 0.0002), the “true” spring ephemeral species *Claytonia virginica* and *Erythronium americanum* had significantly shorter phenophase durations than the other species (Figure 5A2–A4).

**TABLE 3:** Results of negative binomial^1^ or Poisson^2^ generalized linear models (GLMs) testing whether the mean duration of vegetative, flowering, and fruiting phenophases differ among 14 plant species located across five public gardens in the midwest and southeastern United States. Significance at *a* = 0.05 of covariate estimates and overall models are indicated with asterisks.

Plant part	Mean duration of phenophase	Coeff.	GLM				Significance of overall model		
			Coeff. est.	SE	*z*	*p*	*X* ^2^	df	*p*
Vegetative	First Shoot^1^	*Arisaema triphyllum* (Intercept)	1.7918	0.4136	4.332	< 0.001*	19.37	11	0.06
		*Asarum canadense*	1.0415	0.4992	2.086	0.04*			
		*Claytonia virginica*	0.7340	0.5466	1.343	0.18			
		*Erythronium americanum*	0.5108	0.6682	0.764	0.44			
		*Jeffersonia diphylla*	0.4055	0.6765	0.599	0.55			
		*Maianthemum racemosum*	0.4418	0.5151	0.858	0.39			
		*Podophyllum peltatum*	0.7732	0.5052	1.531	0.13			
		*Polygonatum biflorum*	0.6061	0.5516	1.099	0.27			
		*Sanguinaria canadensis*	0.7985	0.5045	1.583	0.11			
		*Stylophorum diphyllum*	0.6061	0.5516	1.099	0.27			
		*Trillium grandiflorum*	1.3581	0.4938	2.750	0.006*			
		*Trillium recurvatum*	−0.5390	0.6338	−0.850	0.40			
	First Leaf Unfolded^1^	*Arisaema triphyllum* (Intercept)	2.52573	0.30131	8.382	< 0.001*	21.08	12	0.05*
		*Asarum canadense*	−0.10981	0.40654	−0.270	0.79			
		*Claytonia virginica*	−1.83258	0.69453	−2.639	0.008*			
		*Erythronium americanum*	−0.73397	0.56187	−1.306	0.19			
		*Jeffersonia diphylla*	0.22581	0.45437	0.497	0.62			
		*Maianthemum racemosum*	0.57661	0.41571	1.387	0.17			
		*Podophyllum peltatum*	−0.44629	0.47628	−0.937	0.35			
		*Polygonatum biflorum*	0.35066	0.41912	0.837	0.40			
	
		*Sanguinaria canadensis*	0.23111	0.42125	0.549	0.58		12	0.05*
		*Stylophorum diphyllum*	0.41871	0.50861	0.823	0.41			
		*Trillium erectum*	0.03922	0.67147	0.058	0.95			
		*Trillium grandiflorum*	0.06953	0.40292	0.173	0.86			
	*Trillium recurvatum*	0.36464	0.41889	0.871	0.38				
	All Leaves Unfolded^1^	*Arisaema triphyllum* (Intercept)	4.199705	0.220644	19.034	< 0.001*	33.80	12	0.0007*
		*Asarum canadense*	0.117783	0.311146	0.379	0.71			
		*Claytonia virginica*	−0.74181	0.298866	−2.482	0.01*			
		*Erythronium americanum*	−1.10866	0.327901	−3.381	0.0007*			
		*Jeffersonia diphylla*	−0.26787	0.314489	−0.852	0.40			
		*Maianthemum racemosum*	0.140848	0.291040	0.484	0.63			
		*Podophyllum peltatum*	−0.22189	0.280427	−0.791	0.43			
		*Polygonatum biflorum*	0.207014	0.310536	0.667	0.51			
		*Sanguinaria canadensis*	−0.00400	0.279117	−0.014	0.99			
		*Stylophorum diphyllum*	0.114444	0.291189	0.393	0.69			
		*Trillium erectum*	−0.26787	0.352156	−0.761	0.45			
		*Trillium grandiflorum*	−0.10120	0.292568	−0.346	0.73			
		*Trillium recurvatum*	−0.43850	0.295399	−1.484	0.14			
	First Leaf Withered^1^	*Arisaema triphyllum* (Intercept)	3.344039	0.279308	11.973	< 0.001*	33.83	10	0.0002*
		*Claytonia virginica*	−0.39960	0.451333	−0.885	0.38			
*Erythronium americanum*		−0.77909	0.412192	−1.890	0.06				
	First Leaf Withered
		*Jeffersonia diphylla*	0.704843	0.387395	1.819	0.07			
		*Podophyllum peltatum*	−0.03349	0.353640	−0.095	0.92			
		*Polygonatum biflorum*	0.681313	0.542784	1.255	0.21			
		*Sanguinaria canadensis*	0.850654	0.386376	2.202	0.03*			
		*Stylophorum diphyllum*	0.750306	0.430951	1.741	0.08			
		*Trillium erectum*	−0.23052	0.446775	−0.516	0.61			
		*Trillium grandiflorum*	0.005865	0.369420	0.016	0.99			
*Trillium recurvatum*	0.667829	0.363626	1.837	0.07					
Flowers	First Bud^1^	*Arisaema triphyllum* (Intercept)	2.7081	0.5698	4.753	< 0.001*	7.0	7	0.43
		*Asarum canadense*	−0.1823	0.6396	−0.285	0.78			
		*Jeffersonia diphylla*	−1.3218	0.9125	−1.449	0.15			
		*Maianthemum racemosum*	−0.5680	0.7159	−0.793	0.43			
		*Polygonatum biflorum*	0.2364	0.7970	0.297	0.77			
		*Stylophorum diphyllum*	−0.4568	0.7115	−0.642	0.52			
		*Trillium grandiflorum*	−0.6931	0.7213	−0.961	0.34			
		*Trillium recurvatum*	0.1133	0.6230	0.182	0.86			
	Bud Burst^1^	*Arisaema triphyllum* (Intercept)	1.37e+0	5.000e‐01	2.773	0.006*	12.80	5	0.03*
		*Asarum canadense*	−1.37e+0	1.118e+0	−1.240	0.22			
		*Maianthemum racemosum*	5.60e‐01	5.669e‐01	0.987	0.32			
		*Stylophorum diphyllum*	−2.90e‐01	7.638e‐01	−0.377	0.71			
		*Trillium grandiflorum*	−2.07e‐17	7.071e‐01	0.000	1.0			
	
	*Trillium recurvatum*	1.01e+00	5.839e‐01	1.733	0.08			
	First Flower^2^	*Arisaema triphyllum* (Intercept)	2.07944	0.25000	8.318	< 0.001*	12.52	6	0.05*
		*Asarum canadense*	−0.13353	0.36596	−0.365	0.72			
		*Erythronium americanum*	−2.07944	1.03078	−2.017	0.04*			
		*Maianthemum racemosum*	−0.06454	0.35940	−0.180	0.86			
		*Sanguinaria canadensis*	−0.13353	0.45316	−0.295	0.77			
		*Stylophorum diphyllum*	−0.47000	0.35940	−1.308	0.19			
		*Trillium grandiflorum*	0.22314	0.33541	0.665	0.51			
	Early Flowering^2^	*Arisaema triphyllum* (Intercept)	2.1972	0.2357	9.322	< 0.001*	35.16	10	0.0001*
		*Asarum canadense*	0.1382	0.2963	0.466	0.64			
		*Claytonia virginica*	−0.4055	0.3727	−1.088	0.28			
		*Erythronium americanum*	0.2007	0.3178	0.631	0.53			
		*Maianthemum racemosum*	−0.9445	0.4454	−2.120	0.03*			
		*Podophyllum peltatum*	−0.2513	0.4454	−0.564	0.57			
		*Sanguinaria canadensis*	−1.2164	0.4249	−2.863	0.004*			
		*Stylophorum diphyllum*	−0.5878	0.3249	−1.809	0.07			
		*Trillium erectum*	−0.2513	0.3563	−0.705	0.48			
		*Trillium grandiflorum*	−1.5041	0.5528	−2.721	0.007*			
		*Trillium recurvatum*	−0.2048	0.3178	−0.644	0.52			
Middle Flowering^1^	*Arisaema triphyllum* (Intercept)	2.756840	0.283419	9.727	< 0.001*	26.50	11	0.005*
	
		*Asarum canadense*	0.454003	0.374104	1.214	0.22			
		*Claytonia virginica*	−0.00531	0.433060	−0.012	0.99			
		*Erythronium americanum*	−1.65822	0.526665	−3.149	0.002*			
		*Maianthemum racemosum*	0.169899	0.429094	0.396	0.69			
		*Podophyllum peltatum*	−0.35895	0.443360	−0.810	0.42			
		*Polygonatum biflorum*	−0.11778	0.494922	−0.238	0.81			
		*Sanguinaria canadensis*	−1.37055	0.766903	−1.787	0.07			
		*Stylophorum diphyllum*	0.226313	0.396785	0.570	0.57			
		*Trillium erectum*	0.076373	0.630051	0.121	0.90			
		*Trillium grandiflorum*	0.064539	0.379202	0.170	0.86			
	*Trillium recurvatum*	0.538997	0.392479	1.373	0.17				
	Late Flowering^1^	*Arisaema triphyllum* (Intercept)	2.169054	0.228604	9.488	< 0.001*	55.05	11	< 0.001*
		*Asarum canadense*	0.948896	0.282948	3.354	0.0008*			
		*Claytonia virginica*	0.433636	0.369693	1.173	0.24			
		*Erythronium americanum*	0.166321	0.340737	0.488	0.63			
		*Maianthemum racemosum*	−0.41985	0.345581	−1.215	0.22			
		*Podophyllum peltatum*	−0.00957	0.349722	−0.027	0.98			
		*Polygonatum biflorum*	0.228842	0.380908	0.601	0.55			
		*Sanguinaria canadensis*	1.838279	0.406441	4.523	< 0.001*			
		*Stylophorum diphyllum*	0.702626	0.320487	2.192	0.03*			
*Trillium erectum*		0.228842	0.487772	0.469	0.64				
	
		*Trillium grandiflorum*	0.364643	0.295099	1.236	0.22			
*Trillium recurvatum*	0.281951	0.297408	0.948	0.34					
Fruits	First Ripe Fruit^1^	*Arisaema triphyllum* (Intercept)	1.98100	0.29782	6.652	< 0.001*	26	12	0.01*
		*Asarum canadense*	−0.18924	0.46534	−0.407	0.68			
		*Claytonia virginica*	−1.98100	1.14261	−1.734	0.08			
		*Erythronium americanum*	−0.51466	0.48776	−1.055	0.29			
		*Jeffersonia diphylla*	0.03390	0.51361	0.066	0.95			
		*Maianthemum racemosum*	−1.28785	0.89753	−1.435	0.15			
		*Podophyllum peltatum*	0.71024	0.39983	1.776	0.08			
		*Polygonatum biflorum*	1.01473	0.59629	1.702	0.09			
		*Sanguinaria canadensis*	0.21622	0.44500	0.486	0.63			
		*Stylophorum diphyllum*	0.68159	0.42923	1.588	0.11			
		*Trillium erectum*	−0.03509	0.66964	−0.052	0.96			
		*Trillium grandiflorum*	−0.18924	0.68719	−0.275	0.78			
		*Trillium recurvatum*	0.72705	0.48007	1.514	0.13			
	Early Fruiting^1^	*Arisaema triphyllum (Intercept)*	3.0603	0.3148	9.722	< 0.001*	22.22	12	0.04*
		*Asarum canadense*	−1.1144	0.5438	−2.049	0.04*			
		*Claytonia virginica*	−0.5754	0.5157	−1.116	0.26			
		*Erythronium americanum*	−1.2144	0.4850	−2.504	0.01*			
		*Jeffersonia diphylla*	−0.2076	0.4492	−0.462	0.64			
	
		*Maianthemum racemosum*	−0.4453	0.4549	−0.979	0.33			
		*Podophyllum peltatum*	−1.2685	0.4880	−2.600	0.009*			
		*Polygonatum biflorum*	−1.9617	0.8263	−2.374	0.018*			
		*Sanguinaria canadensis*	−0.4392	0.4241	−1.036	0.30			
		*Stylophorum diphyllum*	−0.8267	0.4672	−1.769	0.08			
		*Trillium erectum*	−1.9617	0.8263	−2.374	0.018*			
		*Trillium grandiflorum*	−0.6624	0.5194	−1.275	0.20			
	*Trillium recurvatum*	−0.1335	0.4477	−0.298	0.77				
	Middle Fruiting^1^	*Arisaema triphyllum (Intercept)*	3.09104	0.40306	7.669	< 0.001*	12.44	12	0.41
		*Asarum canadense*	0.01130	0.49352	0.023	0.98			
		*Claytonia virginica*	−0.18232	0.52326	−0.348	0.73			
		*Erythronium americanum*	−0.31845	0.57745	−0.551	0.58			
		*Jeffersonia diphylla*	−0.42845	0.52808	−0.811	0.42			
		*Maianthemum racemosum*	0.02985	0.51992	0.057	0.95			
		*Podophyllum peltatum*	−0.18688	0.49601	−0.377	0.71			
		*Polygonatum biflorum*	0.37469	0.68788	0.545	0.59			
		*Sanguinaria canadensis*	0.17472	0.47538	0.368	0.71			
		*Stylophorum diphyllum*	0.26760	0.49094	0.545	0.59			
		*Trillium erectum*	0.95201	0.67784	1.404	0.16			
		*Trillium grandiflorum*	−0.33951	0.52621	−0.645	0.52			
*Trillium recurvatum*		0.54654	0.51419	1.063	0.29				

For the flowering phenophases, *Trillium grandiflorum* had a significantly shorter mean duration of “Early Flowering” than the other species, with early flowering lasting only 2 days (χ^2^(10) = 35.16, *p* = 0.0001; Figure 5B4), while *Erythronium americanum* had a significantly shorter mean duration of “Middle Flowering” at 3 days (χ^2^(11) = 26.50, *p* = 0.005; Figure 5B5) and *Sanguinaria canadensis* had a significantly longer mean duration of “Late Flowering” at 55 days (χ^2^(11) = 55.05, *p* < 0.001; Figure 5B6). Finally, for fruiting phenophases, post hoc tests did not identify pairwise differences in mean durations of phenophases for any of the wildflower species, although two of the three models were significant (“First Ripe Fruit”: χ^2^(12) = 26.00, *p* = 0.01; “Early Fruiting”: χ^2^(12) = 22.22, *p* = 0.04; Figure 5C1–C2).

## 4 Discussion

In this study, we quantified the relationship between warmer‐than‐average days (as measured by deviation in daily mean 2023 temperatures from 30‐year historical means) and the duration of 13 distinct vegetative, flowering, and fruiting phenophases for a suite of spring ephemeral wildflowers across five public gardens in the midwestern and southeastern United States. Our findings can be distilled into three primary takeaways. First, we established that there were numerous instances (an average of 18.8 weeks across all five gardens) in which 2023 weekly mean temperatures deviated significantly from the 30‐year historic norms across the growing season and across all five gardens. Second, we found evidence that positive deviance in 2023 mean daily temperatures from 30‐year historical mean daily temperatures experienced during the phenophase (i.e., significantly warmer‐than‐average days) were related to shorter durations of wildflower phenophases, independent of garden location and plant species. This trend was more pronounced for the earliest phenophases measured. Specifically, this relationship was statistically significant for one flowering phenophase (“First Bud”) and two fruiting phenophases (“First Ripe Fruit” and “Early Fruiting”). Although nonsignificant, similar trends emerged for several other early‐season phenophases across the suite of species. Our pooling of phenophase durations across 14 species and 5 distinct locations in our models establishes these results as robust across herbaceous understory taxa and across regional geographic space within the midwestern and southeastern United States. Third, we identified significant differences in mean phenophase durations among wildflower species, although none of these differences were particularly consistent across broad phenophase categories (vegetative, flowering, and fruiting).

One possible mechanism potentially driving the weakening relationship between phenophase duration and high temperatures as the growing season progressed from spring to summer is the front loading of significantly deviant positive temperatures in 2023 for all five locations (with significantly warmer conditions occurring more frequently in the spring than later in the year for most garden locations; Figure 3; Figure S4; Table S2). While this is true from a numeric perspective (e.g., the raw number of days that were above or below the historic mean maximum or minimum daily temperature was higher for January, February, and March than any other months; Table S2), we found that month was not a significant predictor of the proportion of significantly positively deviant (e.g., “warm”; Binomial GLM; *X* ^2^(9) = 1.65, *p =* 0.99; Figure S5) or significantly negatively deviant (e.g., “cool”; binomial GLM; *X* ^2^(9) = 0.38, *p =* 1.0; Figure S6) days across all garden locations. In other words, while there were more warm days (as compared to the historical baseline) early in the year for all garden locations, this pattern was not statistically significant, and there were significant variations in mean weekly temperature from the historical mean throughout the entire 2023 growing season. Thus, from a statistical standpoint, the front loading of warm daily temperatures early in the spring did not influence our findings that the durations of early‐season phenophases were inversely related to higher deviations in mean 2023 daily temperatures from historic norms.

It makes biological sense that early spring phenophases, especially those related to reproduction (i.e., flowering and fruiting), would progress more quickly in response to high daily temperatures than later‐season vegetative phenophases. The species in our study are either “true” spring ephemerals (e.g., Yancy et al. 2023), or spring wildflowers, and are thus adapted to take advantage of relatively cool (but not freezing) temperatures and high levels of light prior to forest canopy development and closure. As a group, spring ephemeral herbs exhibit temperature‐ and light‐sensitive cues to increase fitness and survival, including avoiding frost damage and mortality by postponing emergence until a chilling requirement is met (Risser and Cottam 1967; Yoshie 2008) and capitalizing on intense spring light for carbon fixation and carbohydrate accumulation (Lapointe 2001; Augspurger and Salk 2017). Quicker completion of the reproductive portion of their phenology under high spring temperatures may be beneficial, especially since warmer temperatures can advance closure of the forest canopy (Fu et al. 2014; Dow et al. 2022). Plants that bloom early in the growing season tend to produce mature fruit earlier (Peñuelas, Filella, and Comas 2002; Sherry et al. 2007; Post et al. 2008), and our results reinforce this finding. However, it is impossible to determine from our study whether the shorter duration of fruiting phenophases affiliated with high daily temperatures early in the season is a result of the more rapid progression of flowering phenophases (i.e., reproduction is tightly integrated across the life cycle; Pigliucci 2003), or because fruit development and maturation are able to respond independently to environmental conditions (Haggerty and Galloway 2011).

Our finding that the early‐season reproductive phenophases progress quickly under high daily temperatures indicates that these phenophases may be more tightly linked to abiotic cues such as temperature than to vegetative phenophases. Early and rapid completion of flowering and fruiting may also confer competitive benefits for mutualist‐dependent services, such as early‐season pollination (Gezon, Inouye, and Irwin 2016) and seed dispersal, although only if mutualistic partners are also able to adjust their phenology in response to warm temperatures—an assumption for which there is conflicting evidence (Rafferty, CaraDonna, and Bronstein 2015; Kharouba et al. 2018). Multiple (potentially interactive) mechanisms could explain our finding that later‐season vegetative phenology was less sensitive to positive deviations from mean historical temperatures. The longer a perennial spring herb stays green, presumably the more carbon and nutrients the plant is able to sequester for the future. Since spring ephemerals have low nutrient absorption rates and high nutrient requirements, plants must stay green long enough to replenish their nutrient reserves, irrespective of external temperatures (Lapointe 2001). That is to say, there may be higher pressures (and benefits) driving the rapid advancement through early phenophases relative to later phenophases, ultimately facilitating carbon capture and reproductive output.

The finding that the duration of vegetative phenophases was significantly shorter for the true spring ephemerals *C. virginica* and *E. americanum* than any other species in the study is interesting from a natural history perspective. The inherently short duration of these phenophases may be concerning in light of climate change, as the already narrow window of time before canopy closure is expected to become even shorter (Heberling et al. 2019). Furthermore, that we detected interspecific differences in the durations of individual phenophases in gardens across a broad geographic extent supports the logic that there are drivers governing phenology beyond temperature alone. These likely include a combination of additional abiotic cues (precipitation, light availability, etc) and biotic underpinnings, such as adaptation (Wilczek et al. 2010) or phylogeny (Li et al. 2016). Donnelly et al. (2017) emphasized the importance of life history strategy and ecological niche in determining plant phenophase duration. The fact that the “true” spring ephemerals in our study were the two species with the shortest vegetative phenophases supports this to some extent. However, all of the species in our study overlap significantly in life history strategies and ecological niches, so the finding that durations of individual phenophases differed among species within this narrow functional group is interesting.

Our ability to extrapolate these results across broader suites of plants, geographies, and/or habitats is limited by our inclusion only of spring‐flowering understory forest herbs at five public gardens in the midwestern and southeastern United States. Furthermore, it is important to note that our dataset only includes one year's worth of phenology data, meaning we cannot definitively establish that phenophase duration for any of the study species in any of the study sites differed relative to a “normal” year. However, our statistical comparison of temperatures during the 2023 growing season relative to 30‐year historic means does allow us to identify unusually warm or cool days for each location. That there were consistent trends in the duration of phenophases related to the magnitude of deviation in mean daily temperature between Year 2023 to date and the 30‐year historic means—across both species and garden locations—provides compelling evidence that early‐season spring ephemeral phenophase durations may be truncated by higher than normal daily temperatures, especially in the spring. Future studies attempting to explore the relationship between extreme temperature events and plant phenophase duration across multiple years would help to confirm this trend. Finally, Körner and Hiltbrunner (2018) emphasize the importance of selecting the appropriate measures of temperature in order to describe habitat conditions and understand the mechanisms underpinning plant responses. Our use of temperature data collected at coarser geographic scales (aggregated to city level) could impact results as individual understory herbs respond to conditions at the microsite level. Here, we use air temperature data, rather than soil temperature, which may be particularly relevant for phenophases such as plant emergence. Future studies could remedy this by employing fine‐scale monitoring of both air and soil temperatures within the microsites inhabited by individual plants.

Scaling plant phenology from individual species to the landscape level is critical, yet implementation remains challenging (Piao et al. 2019). Despite rapid technological advances in remote sensing, continuous in situ monitoring of understory plant phenology, including the start and end of the carbon uptake process and the subtle transitions among both reproductive and vegetative phenophases, remains crucial (Donnelly et al. 2017). In this study, we demonstrate a viable and widely applicable community science approach capable of capturing understory plant phenology data at a fine temporal scale and across a large geographic region using a relatively unique combination of community science and public garden collections. Community science offers a rapidly growing platform to facilitate data collection at temporal and spatial scales unfeasible by the scientific community alone (Heberling et al. 2021). Such community science work has been greatly facilitated in recent years by the emergence of dedicated apps that streamline data collection by community scientists, including iNaturalist and the Budburst app (used here). Public gardens themselves offer powerful platforms for the study of climate change impacts (Donaldson 2009; Primack and Miller‐Rushing 2009; Primack et al. 2021), but can further facilitate the work of community scientists by offering easy access to and identification of plants for research in *de facto* common gardens.

In conclusion, the results of this study established that short periods of positive temperature deviations from the historic norm (i.e., warmer‐than‐average days) early in the growing season of 2023 were related to truncated interphase durations of reproductive phenophases for spring flowering herbs. Our study also established that two aspects of plant phenology related to reproduction (flowering and fruiting) were more sensitive to extreme daily temperatures than were vegetative phenophases and that these sensitivities were detected across the entire suite of spring wildflower species and gardens in the study. The pooling of data across 14 species and five geographically distinct locations establishes these results as robust for spring‐flowering herbaceous understory taxa across regional geographic space within the midwestern and southeastern United States, thus informing phenological responses to short‐lived weather cues and thus future conservation efforts for this group of plants.

## Author Contributions

**Chelsea N. Miller:** conceptualization (lead), data curation (lead), formal analysis (lead), funding acquisition (lead), investigation (lead), methodology (equal), project administration (lead), resources (equal), visualization (lead), writing – original draft (lead), writing – review and editing (equal). **Katharine L. Stuble:** conceptualization (supporting), data curation (supporting), formal analysis (supporting), funding acquisition (supporting), investigation (supporting), methodology (equal), project administration (supporting), resources (equal), visualization (supporting), writing – original draft (supporting).

## Conflicts of Interest

The authors declare no conflicts of interest.

## Supporting information


Data S1.

## Data Availability

All phenology data related to the article are free and publicly available on the Dryad Data Repository: http://datadryad.org/stash/share/gBWbyXDkAhobNV7xphqraW67bD24vI5_RTcoyO‐ohss.
