# Correction to “Warm Spring Days Are Related to Shorter Durations of Reproductive Phenophases for Understory Forest Herbs”

**DOI:** 10.1002/ece3.71219

**Published:** 2025-04-03

Miller, C. N., and K. L. Stuble. 2024. “Warm Spring Days Are Related to Shorter Durations of Reproductive Phenophases for Understory Forest Herbs.” Ecology and Evolution 14: e70700. https://doi.org/10.1002/ece3.70700.

In the published article, a temporary URL was included in the Data Availability Statement. The data can now be accessed at the permanent URL: https://doi.org/10.5061/dryad.jh9w0vtmh.

We apologize for this error.

