# Supplementary material for: Warm Spring Days are Related to Shorter Durations of Reproductive Phenophases for Understory Forest Herbs
Source: Ecol Evol. 2024 Dec 16;14(12):e70700. doi: 10.1002/ece3.70700 (PMC11650752; doi:10.1002/ece3.70700)
Supplement: Supplementary file 1 — Data S1. [file ECE3-14-e70700-s001.docx]

**Supplemental Materials**

**
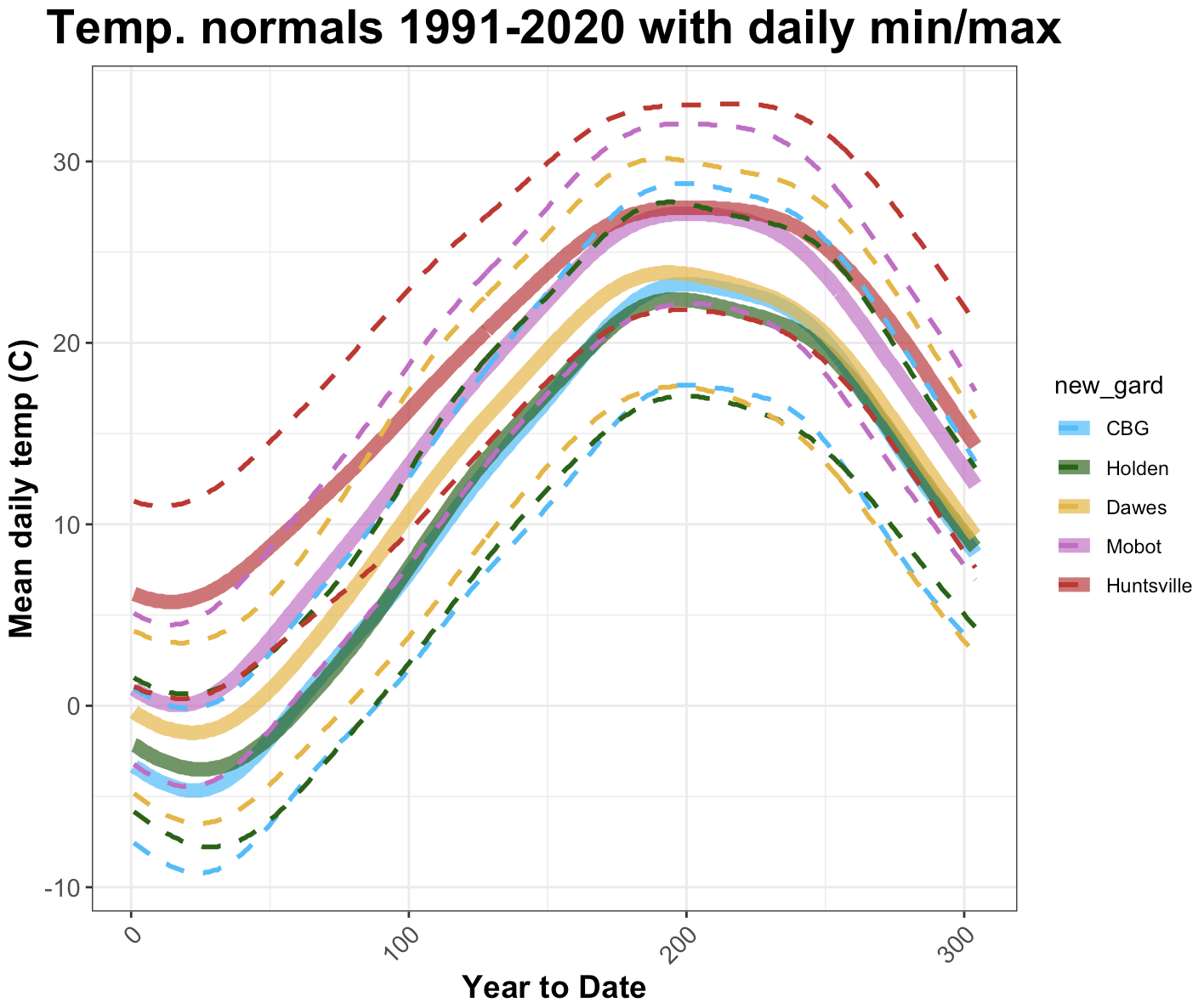
**

**Figure 1:** Solid lines indicate mean daily temperature (°C) averaged for 30 years (1991-2020) for each garden location from Jan. 1 – Oct. 31. Dashed lines indicate mean daily maximum and minimum temperatures (°C) averaged for 30 years (1991-2020) for each garden location. Blue = Chicago Botanic Garden, Green = Holden Arboretum, Yellow = Dawes Arboretum, Pink = Missouri Botanical Garden, Red = Huntsville Botanical Garden.


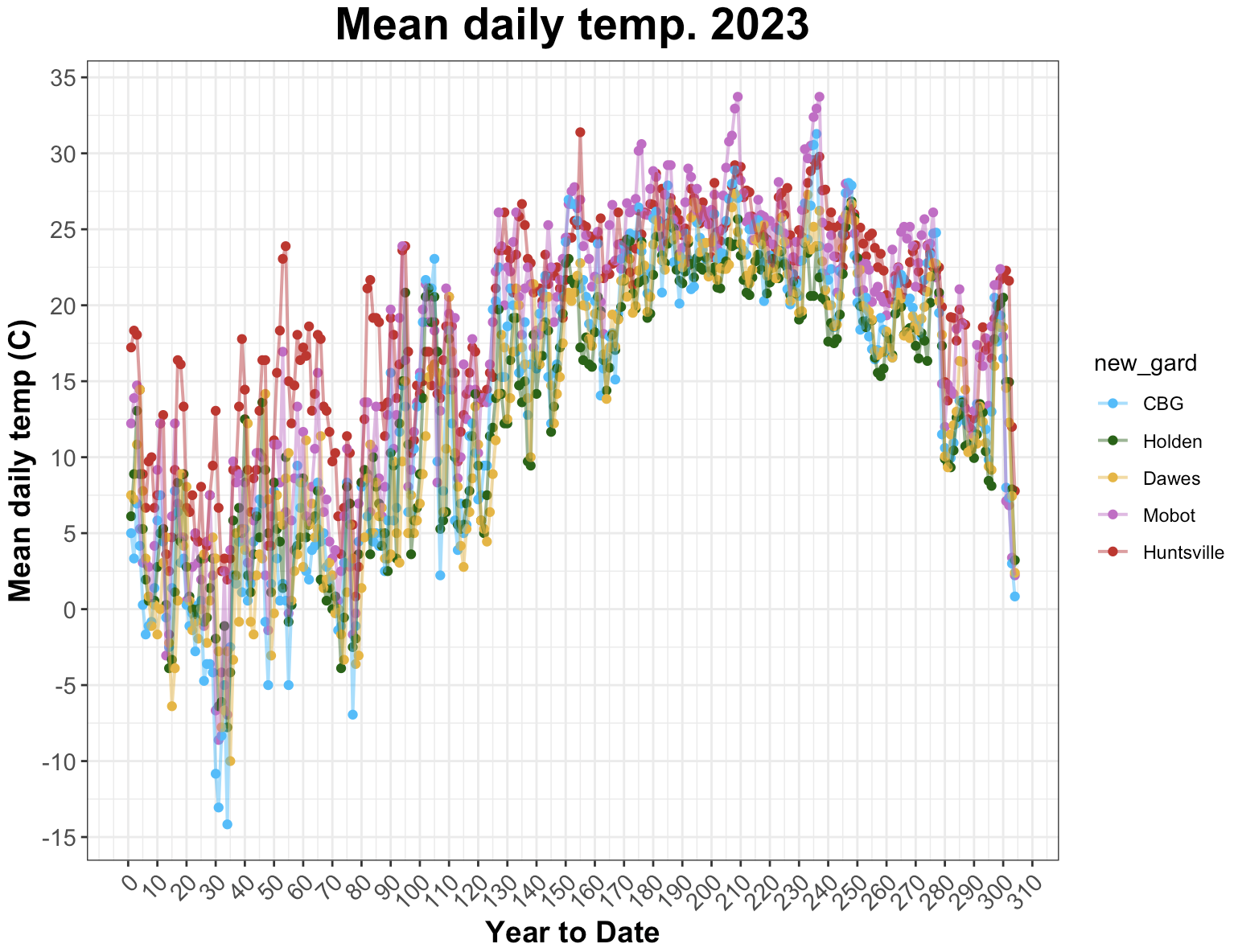


**Figure 2:** Time series indicating mean daily temperature (°C) of Huntsville, AL (Huntsville Botanical Garden; red), St. Louis, MO (Missouri Botanical Garden; pink), Newark, OH (Dawes Arboretum; yellow), Kirtland, OH (Holden Arboretum, green), and Chicago, IL (Chicago Botanic Garden, blue) from Jan. 1 – Oct. 31, 2023.


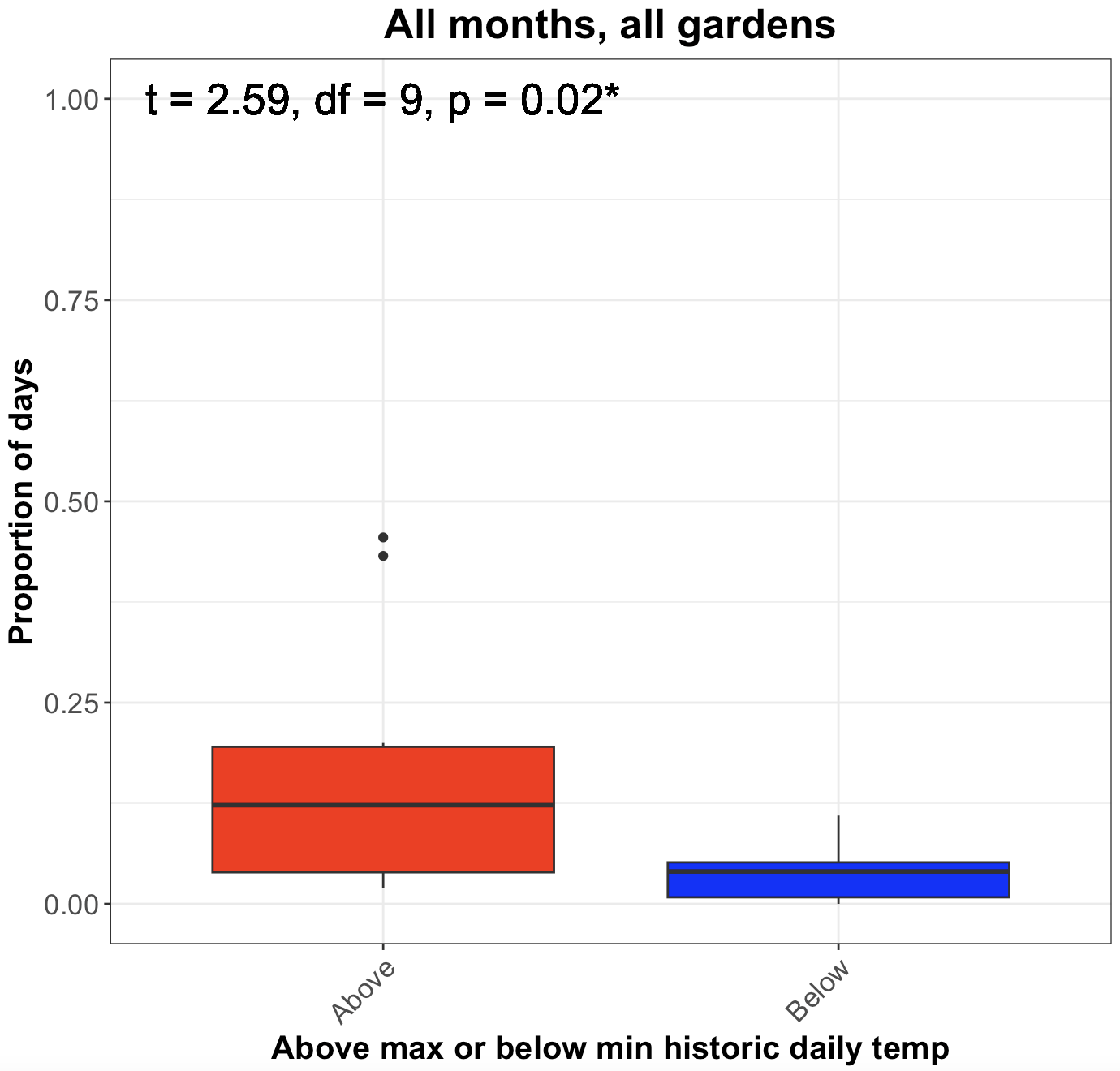


**Figure 3:** Boxplot illustrating the proportion of days during the 2023 growing season across all gardens for which the mean daily temperature (°C) exceeded the mean maximum historic daily temperature (“Above” - red) or was lower than the mean minimum historic daily temperature (“Below” - blue). A paired t-test revealed that there was a greater proportion of days above the mean maximum historic daily temperatures (median = 0.17) than days below mean minimum historic daily temperatures (median = 0.05) (t = 2.59, df = 9, *p* = 0.02).


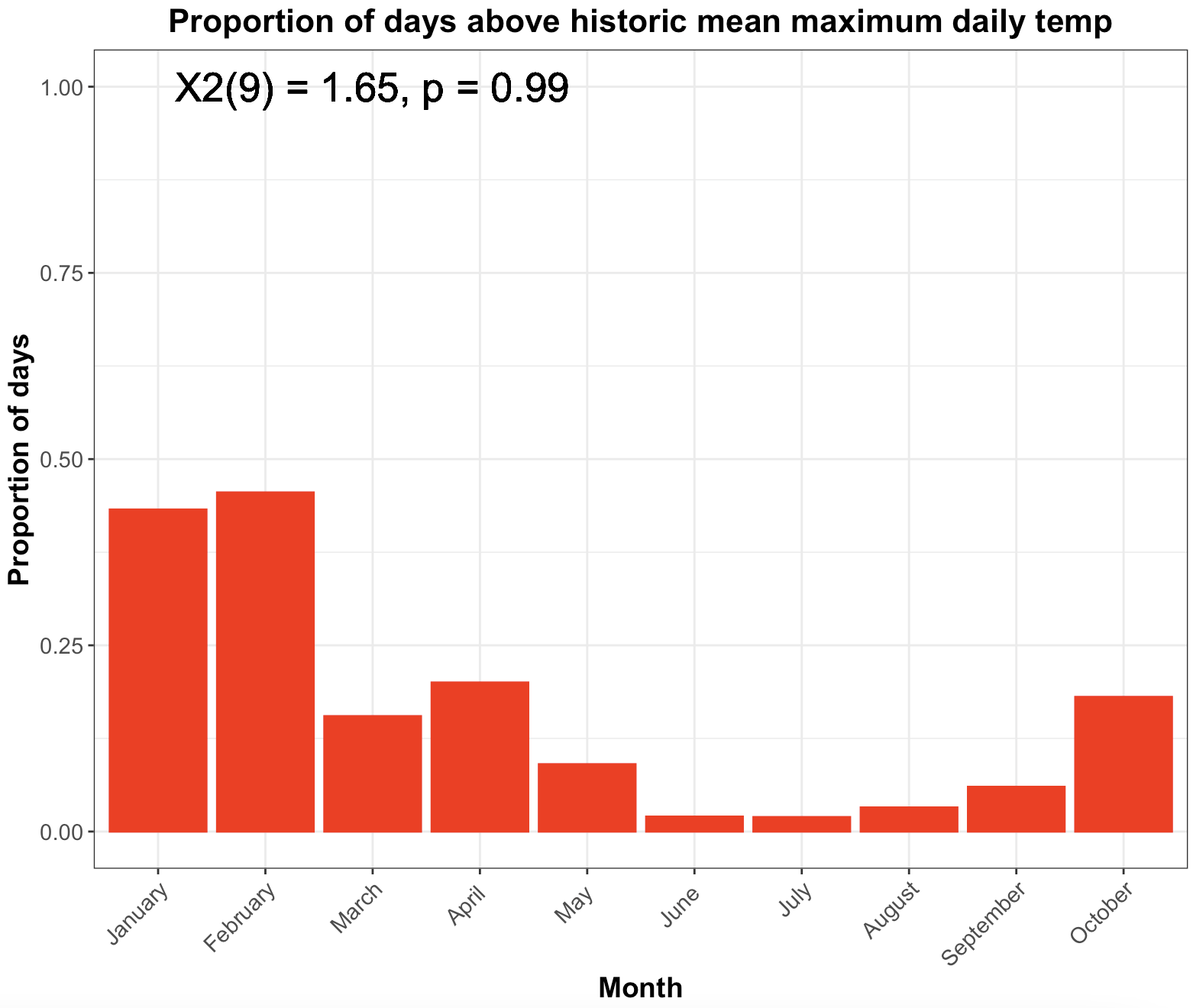


**Figure 4:** Barplot illustrating the proportion of days per month during the 2023 growing season across all gardens for which the mean daily temperature (°F) exceeded the mean maximum historic daily temperature (°C). A binomially-distributed GLM indicated that month (i.e., time of year) was not a significant predictor of the proportion of days exceeding historic mean maximum daily temperatures (*X*^2^(9) = 1.65, *p* = 0.99).


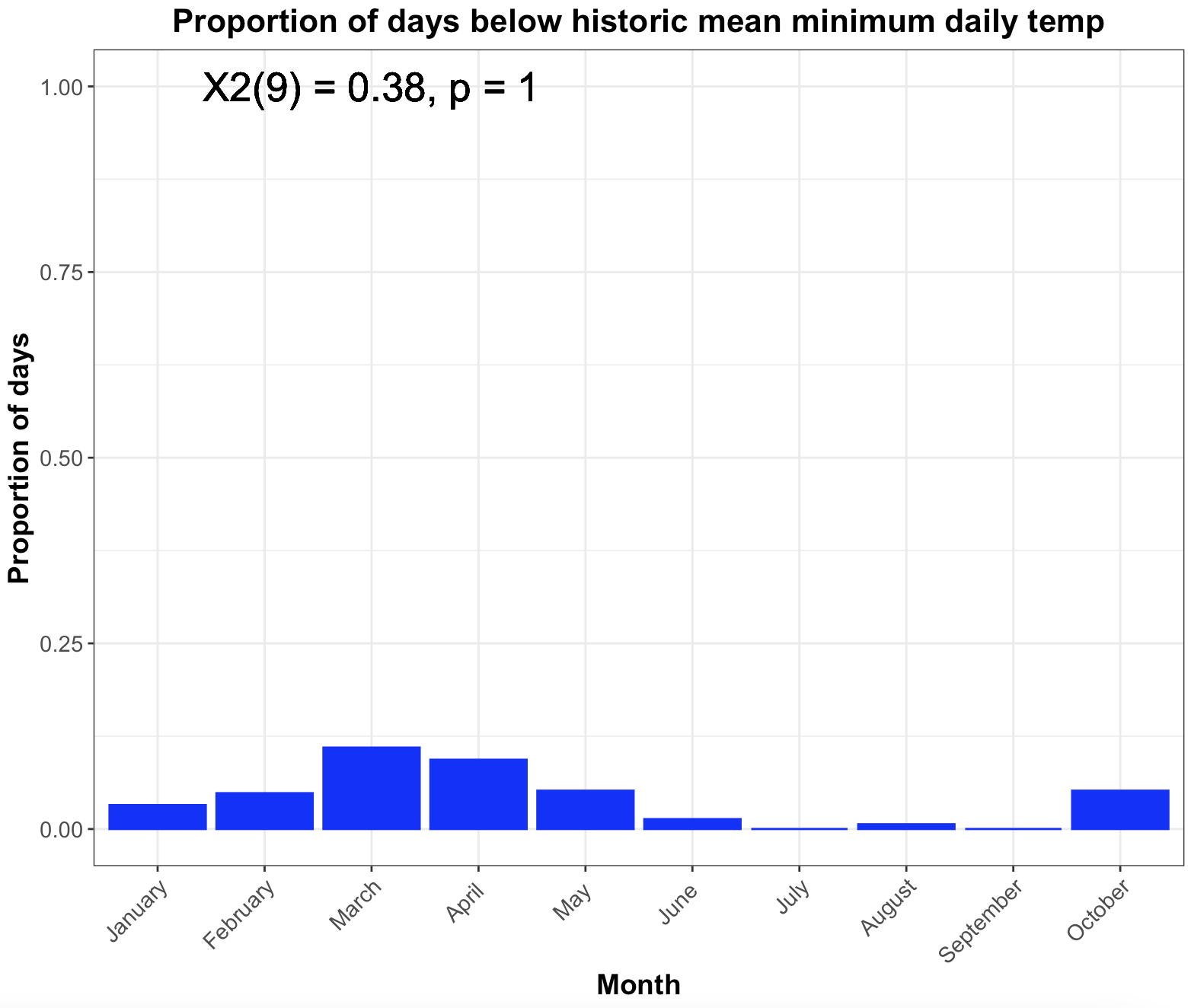


**Figure 5:** Barplot illustrating the proportion of days per month during the 2023 growing season across all gardens for which the mean daily temperature (°F) was lower than the mean minimum historic daily temperature (°C). A binomially-distributed GLM indicated that month (i.e., time of year) was not a significant predictor of the proportion of days below historic mean minimum daily temperatures (*X*^2^(9) = 0.38, *p* = 1.0).


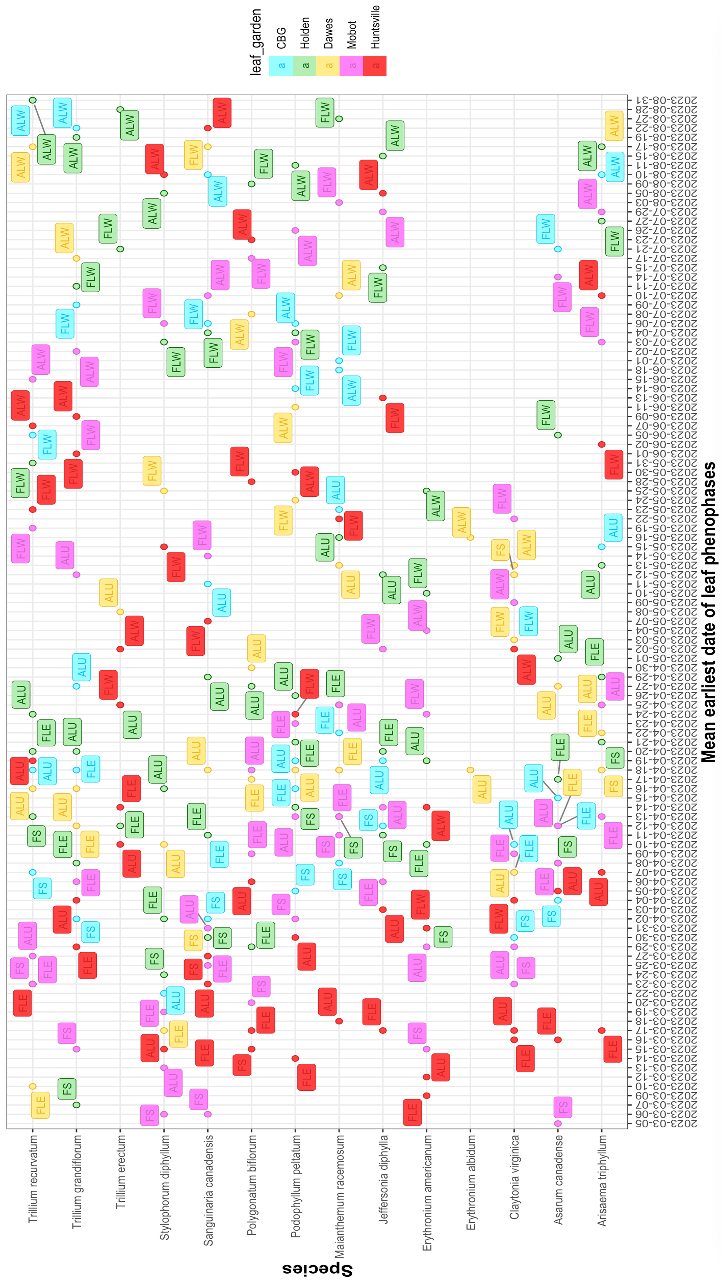


**Figure 6:** Mean earliest dates of leaf phenophases (FS = First Shoot, FLE = First Leaf Emerged, ALU = All Leaves Unfolded, FLW = First Leaf Withered, ALW = All Leaves Withered) for each species at each garden.


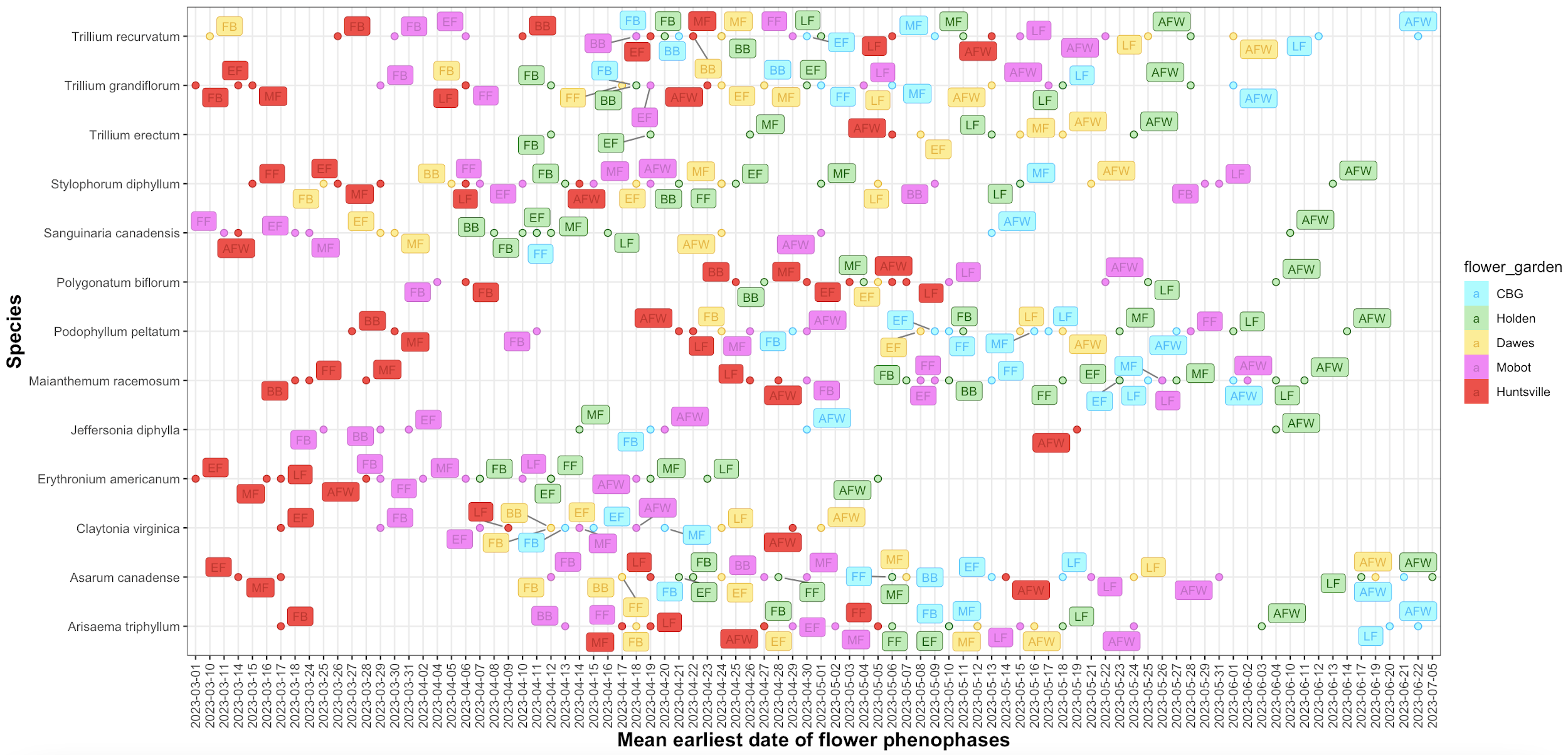


**Figure 7:** Mean earliest dates of flower phenophases (FB = First Bud, BB = Bud Burst, FF = First Flower, EF = Early Flowering, MF = Middle Flowering, LF = Late Flowering, AFW = All Flowers Withered) for each species at each garden.


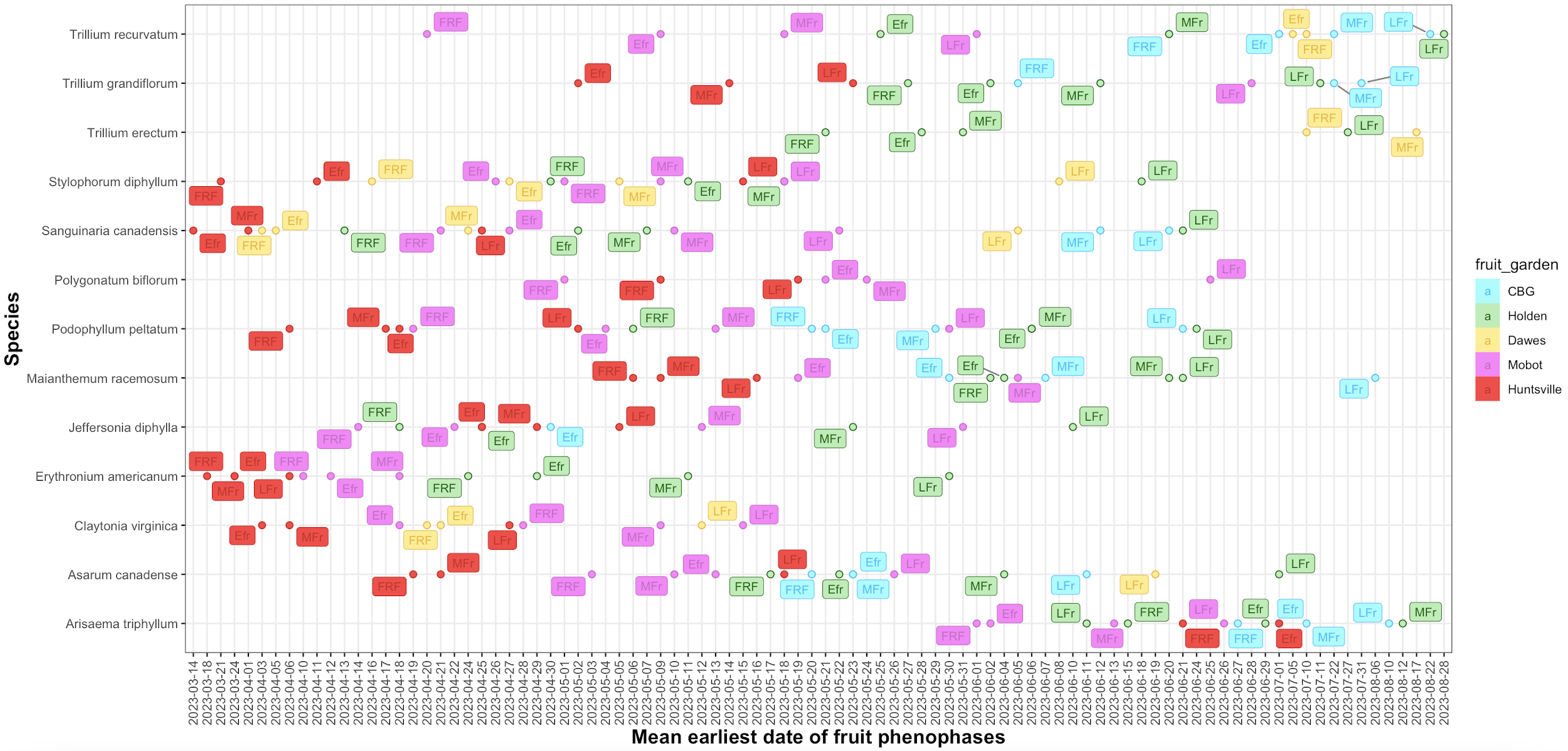


**Figure 8:** Mean earliest dates of fruit phenophases (FB = First Bud, BB = Bud Burst, FF = First Flower, EF = Early Flowering, MF = Middle Flowering, LF = Late Flowering, AFW = All Flowers Withered) for each species at each garden.

**Supplemental Tables**

| **Garden** | **Date** | **Year to Date (YTD)** | **30 year daily normals (ºC)** | **30 year daily max (ºC)** | **30 year daily min (ºC)** | **2023 mean daily temp (ºC)** | **Deviation (2023 daily temp - 30 year normals)** | **Week** | **T-statistic** | **P-value** |
| --- | --- | --- | --- | --- | --- | --- | --- | --- | --- | --- |
| Huntsville | 2024-01-01 | 1 | 6.17 | 11.28 | 1.06 | 1.70 | 11.06 | Week 1 |  |  |
| Huntsville | 2024-01-02 | 2 | 6.11 | 11.22 | 1.00 | 1.70 | 12.22 | Week 1 |  |  |
| Huntsville | 2024-01-03 | 3 | 6.06 | 11.17 | 0.94 | 1.70 | 12.00 | Week 1 |  |  |
| Huntsville | 2024-01-04 | 4 | 6.00 | 11.11 | 0.83 | 1.71 | 8.44 | Week 1 |  |  |
| Huntsville | 2024-01-05 | 5 | 5.94 | 11.11 | 0.78 | 1.72 | 2.94 | Week 1 |  |  |
| Huntsville | 2024-01-06 | 6 | 5.89 | 11.06 | 0.72 | 1.72 | 0.78 | Week 1 |  |  |
| Huntsville | 2024-01-07 | 7 | 5.89 | 11.06 | 0.72 | 1.72 | 3.83 | **Week 1** | **4.082** | **0.006** |
| Huntsville | 2024-01-08 | 8 | 5.83 | 11.00 | 0.67 | 1.72 | 4.17 | Week 2 |  |  |
| Huntsville | 2024-01-09 | 9 | 5.78 | 11.00 | 0.61 | 1.73 | 0.89 | Week 2 |  |  |
| Huntsville | 2024-01-10 | 10 | 5.78 | 11.00 | 0.56 | 1.74 | 1.72 | Week 2 |  |  |
| Huntsville | 2024-01-11 | 11 | 5.78 | 11.00 | 0.50 | 1.74 | 6.44 | Week 2 |  |  |
| Huntsville | 2024-01-12 | 12 | 5.72 | 11.00 | 0.50 | 1.74 | 7.06 | Week 2 |  |  |
| Huntsville | 2024-01-13 | 13 | 5.72 | 11.00 | 0.44 | 1.75 | -2.11 | Week 2 |  |  |
| Huntsville | 2024-01-14 | 14 | 5.72 | 11.00 | 0.44 | 1.75 | -3.22 | Week 2 | 1.417 | 0.206 |
| Huntsville | 2024-01-15 | 15 | 5.72 | 11.06 | 0.44 | 1.76 | -1.00 | Week 3 |  |  |
| Huntsville | 2024-01-16 | 16 | 5.72 | 11.06 | 0.39 | 1.77 | 3.44 | Week 3 |  |  |
| Huntsville | 2024-01-17 | 17 | 5.72 | 11.11 | 0.39 | 1.78 | 10.67 | Week 3 |  |  |
| Huntsville | 2024-01-18 | 18 | 5.78 | 11.11 | 0.39 | 1.78 | 10.33 | Week 3 |  |  |
| Huntsville | 2024-01-19 | 19 | 5.78 | 11.17 | 0.39 | 1.79 | 7.56 | Week 3 |  |  |
| Huntsville | 2024-01-20 | 20 | 5.83 | 11.22 | 0.39 | 1.80 | 0.83 | Week 3 |  |  |
| Huntsville | 2024-01-21 | 21 | 5.83 | 11.28 | 0.44 | 1.80 | 0.56 | **Week 3** | **2.520** | **0.045** |
| Huntsville | 2024-01-22 | 22 | 5.89 | 11.33 | 0.44 | 1.81 | 1.61 | Week 4 |  |  |
| Huntsville | 2024-01-23 | 23 | 5.94 | 11.39 | 0.44 | 1.82 | -1.22 | Week 4 |  |  |
| Huntsville | 2024-01-24 | 24 | 6.00 | 11.44 | 0.50 | 1.82 | -1.56 | Week 4 |  |  |
| Huntsville | 2024-01-25 | 25 | 6.06 | 11.56 | 0.56 | 1.83 | 2.00 | Week 4 |  |  |
| Huntsville | 2024-01-26 | 26 | 6.11 | 11.61 | 0.56 | 1.84 | -2.78 | Week 4 |  |  |
| Huntsville | 2024-01-27 | 27 | 6.17 | 11.67 | 0.61 | 1.84 | -2.00 | Week 4 |  |  |
| Huntsville | 2024-01-28 | 28 | 6.22 | 11.78 | 0.67 | 1.85 | 1.28 | Week 4 | -0.517 | 0.624 |
| Huntsville | 2024-01-29 | 29 | 6.33 | 11.89 | 0.72 | 1.86 | 3.11 | Week 5 |  |  |
| Huntsville | 2024-01-30 | 30 | 6.39 | 11.94 | 0.83 | 1.85 | 6.67 | Week 5 |  |  |
| Huntsville | 2024-01-31 | 31 | 6.50 | 12.06 | 0.89 | 1.86 | 0.17 | Week 5 |  |  |
| Huntsville | 2024-02-01 | 32 | 6.56 | 12.17 | 0.94 | 1.86 | -4.06 | Week 5 |  |  |
| Huntsville | 2024-02-02 | 33 | 6.67 | 12.28 | 1.06 | 1.86 | -3.33 | Week 5 |  |  |
| Huntsville | 2024-02-03 | 34 | 6.78 | 12.39 | 1.11 | 1.87 | -4.83 | Week 5 |  |  |
| Huntsville | 2024-02-04 | 35 | 6.89 | 12.50 | 1.22 | 1.87 | -3.56 | Week 5 | -0.508 | 0.629 |
| Huntsville | 2024-02-05 | 36 | 6.94 | 12.61 | 1.33 | 1.87 | 2.22 | Week 6 |  |  |
| Huntsville | 2024-02-06 | 37 | 7.06 | 12.72 | 1.44 | 1.87 | 2.11 | Week 6 |  |  |
| Huntsville | 2024-02-07 | 38 | 7.22 | 12.89 | 1.50 | 1.89 | 6.11 | Week 6 |  |  |
| Huntsville | 2024-02-08 | 39 | 7.33 | 13.00 | 1.61 | 1.89 | 10.44 | Week 6 |  |  |
| Huntsville | 2024-02-09 | 40 | 7.44 | 13.11 | 1.72 | 1.89 | 7.00 | Week 6 |  |  |
| Huntsville | 2024-02-10 | 41 | 7.56 | 13.28 | 1.83 | 1.90 | 1.61 | Week 6 |  |  |
| Huntsville | 2024-02-11 | 42 | 7.67 | 13.39 | 1.94 | 1.90 | -1.28 | **Week 6** | **2.677** | **0.037** |
| Huntsville | 2024-02-12 | 43 | 7.83 | 13.56 | 2.11 | 1.90 | 0.78 | Week 7 |  |  |
| Huntsville | 2024-02-13 | 44 | 7.94 | 13.67 | 2.22 | 1.90 | 1.22 | Week 7 |  |  |
| Huntsville | 2024-02-14 | 45 | 8.06 | 13.83 | 2.33 | 1.91 | 5.00 | Week 7 |  |  |
| Huntsville | 2024-02-15 | 46 | 8.22 | 13.94 | 2.44 | 1.91 | 8.17 | Week 7 |  |  |
| Huntsville | 2024-02-16 | 47 | 8.33 | 14.11 | 2.56 | 1.92 | 8.06 | Week 7 |  |  |
| Huntsville | 2024-02-17 | 48 | 8.50 | 14.22 | 2.72 | 1.91 | -4.33 | Week 7 |  |  |
| Huntsville | 2024-02-18 | 49 | 8.61 | 14.39 | 2.83 | 1.92 | -4.44 | Week 7 | 1.034 | 0.341 |
| Huntsville | 2024-02-19 | 50 | 8.78 | 14.56 | 2.94 | 1.93 | 2.33 | Week 8 |  |  |
| Huntsville | 2024-02-20 | 51 | 8.89 | 14.72 | 3.11 | 1.93 | 6.67 | Week 8 |  |  |
| Huntsville | 2024-02-21 | 52 | 9.06 | 14.83 | 3.22 | 1.93 | 9.28 | Week 8 |  |  |
| Huntsville | 2024-02-22 | 53 | 9.17 | 15.00 | 3.33 | 1.94 | 13.89 | Week 8 |  |  |
| Huntsville | 2024-02-23 | 54 | 9.33 | 15.17 | 3.50 | 1.94 | 14.56 | Week 8 |  |  |
| Huntsville | 2024-02-24 | 55 | 9.44 | 15.33 | 3.61 | 1.95 | 5.56 | Week 8 |  |  |
| Huntsville | 2024-02-25 | 56 | 9.61 | 15.44 | 3.72 | 1.95 | 2.61 | **Week 8** | **4.177** | **0.006** |
| Huntsville | 2024-02-26 | 57 | 9.72 | 15.61 | 3.89 | 1.95 | 5.00 | Week 9 |  |  |
| Huntsville | 2024-02-27 | 58 | 9.89 | 15.78 | 4.00 | 1.96 | 8.17 | Week 9 |  |  |
| Huntsville | 2024-02-28 | 59 | 10.00 | 15.94 | 4.11 | 1.97 | 6.39 | Week 9 |  |  |
| Huntsville | 2024-03-01 | 60 | 10.17 | 16.11 | 4.22 | 1.98 | 7.06 | Week 9 |  |  |
| Huntsville | 2024-03-02 | 61 | 10.33 | 16.28 | 4.39 | 1.98 | 6.33 | Week 9 |  |  |
| Huntsville | 2024-03-03 | 62 | 10.44 | 16.39 | 4.50 | 1.98 | 8.17 | Week 9 |  |  |
| Huntsville | 2024-03-04 | 63 | 10.61 | 16.56 | 4.61 | 1.98 | 2.44 | **Week 9** | **8.221** | **<0.001** |
| Huntsville | 2024-03-05 | 64 | 10.72 | 16.72 | 4.72 | 1.99 | 3.44 | Week 10 |  |  |
| Huntsville | 2024-03-06 | 65 | 10.89 | 16.89 | 4.89 | 1.99 | 7.17 | Week 10 |  |  |
| Huntsville | 2024-03-07 | 66 | 11.06 | 17.06 | 5.00 | 2.00 | 6.72 | Week 10 |  |  |
| Huntsville | 2024-03-08 | 67 | 11.17 | 17.22 | 5.11 | 2.01 | 2.17 | Week 10 |  |  |
| Huntsville | 2024-03-09 | 68 | 11.33 | 17.39 | 5.22 | 2.02 | 1.72 | Week 10 |  |  |
| Huntsville | 2024-03-10 | 69 | 11.44 | 17.56 | 5.33 | 2.03 | 0.22 | Week 10 |  |  |
| Huntsville | 2024-03-11 | 70 | 11.61 | 17.72 | 5.50 | 2.03 | -1.89 | Week 10 | 2.245 | 0.066 |
| Huntsville | 2024-03-12 | 71 | 11.72 | 17.89 | 5.61 | 2.04 | -1.44 | Week 11 |  |  |
| Huntsville | 2024-03-13 | 72 | 11.89 | 18.06 | 5.72 | 2.05 | -5.78 | Week 11 |  |  |
| Huntsville | 2024-03-14 | 73 | 12.06 | 18.22 | 5.83 | 2.06 | -8.44 | Week 11 |  |  |
| Huntsville | 2024-03-15 | 74 | 12.17 | 18.39 | 5.94 | 2.07 | -5.50 | Week 11 |  |  |
| Huntsville | 2024-03-16 | 75 | 12.33 | 18.56 | 6.11 | 2.07 | -0.94 | Week 11 |  |  |
| Huntsville | 2024-03-17 | 76 | 12.50 | 18.78 | 6.22 | 2.09 | -2.22 | Week 11 |  |  |
| Huntsville | 2024-03-18 | 77 | 12.61 | 18.94 | 6.33 | 2.10 | -7.06 | **Week 11** | **-4.034** | **0.007** |
| Huntsville | 2024-03-19 | 78 | 12.78 | 19.11 | 6.44 | 2.10 | -11.94 | Week 12 |  |  |
| Huntsville | 2024-03-20 | 79 | 12.94 | 19.28 | 6.56 | 2.11 | -10.17 | Week 12 |  |  |
| Huntsville | 2024-03-21 | 80 | 13.06 | 19.44 | 6.72 | 2.11 | -4.72 | Week 12 |  |  |
| Huntsville | 2024-03-22 | 81 | 13.22 | 19.61 | 6.83 | 2.12 | -0.72 | Week 12 |  |  |
| Huntsville | 2024-03-23 | 82 | 13.39 | 19.83 | 6.94 | 2.14 | 7.72 | Week 12 |  |  |
| Huntsville | 2024-03-24 | 83 | 13.56 | 20.00 | 7.11 | 2.14 | 8.11 | Week 12 |  |  |
| Huntsville | 2024-03-25 | 84 | 13.72 | 20.17 | 7.22 | 2.15 | 5.44 | Week 12 | -0.284 | 0.786 |
| Huntsville | 2024-03-26 | 85 | 13.83 | 20.33 | 7.39 | 2.15 | 5.33 | Week 13 |  |  |
| Huntsville | 2024-03-27 | 86 | 14.00 | 20.50 | 7.50 | 2.16 | 4.89 | Week 13 |  |  |
| Huntsville | 2024-03-28 | 87 | 14.17 | 20.72 | 7.61 | 2.18 | -0.83 | Week 13 |  |  |
| Huntsville | 2024-03-29 | 88 | 14.33 | 20.89 | 7.78 | 2.18 | -2.94 | Week 13 |  |  |
| Huntsville | 2024-03-30 | 89 | 14.50 | 21.06 | 7.94 | 2.18 | -0.89 | Week 13 |  |  |
| Huntsville | 2024-03-31 | 90 | 14.67 | 21.22 | 8.06 | 2.19 | 4.50 | Week 13 |  |  |
| Huntsville | 2024-04-01 | 91 | 14.78 | 21.39 | 8.22 | 2.19 | 3.28 | Week 13 | 1.496 | 0.185 |
| Huntsville | 2024-04-02 | 92 | 14.94 | 21.56 | 8.33 | 2.20 | -1.06 | Week 14 |  |  |
| Huntsville | 2024-04-03 | 93 | 15.11 | 21.78 | 8.50 | 2.21 | 1.00 | Week 14 |  |  |
| Huntsville | 2024-04-04 | 94 | 15.28 | 21.94 | 8.67 | 2.21 | 8.33 | Week 14 |  |  |
| Huntsville | 2024-04-05 | 95 | 15.44 | 22.11 | 8.83 | 2.21 | 8.44 | Week 14 |  |  |
| Huntsville | 2024-04-06 | 96 | 15.61 | 22.28 | 8.94 | 2.22 | 1.33 | Week 14 |  |  |
| Huntsville | 2024-04-07 | 97 | 15.78 | 22.44 | 9.11 | 2.22 | -5.50 | Week 14 |  |  |
| Huntsville | 2024-04-08 | 98 | 15.94 | 22.61 | 9.28 | 2.22 | -4.83 | Week 14 | 0.519 | 0.622 |
| Huntsville | 2024-04-09 | 99 | 16.11 | 22.78 | 9.44 | 2.22 | -1.39 | Week 15 |  |  |
| Huntsville | 2024-04-10 | 100 | 16.28 | 22.94 | 9.61 | 2.22 | -2.11 | Week 15 |  |  |
| Huntsville | 2024-04-11 | 101 | 16.44 | 23.11 | 9.78 | 2.22 | -1.44 | Week 15 |  |  |
| Huntsville | 2024-04-12 | 102 | 16.61 | 23.28 | 9.94 | 2.22 | 0.33 | Week 15 |  |  |
| Huntsville | 2024-04-13 | 103 | 16.78 | 23.44 | 10.11 | 2.22 | 0.17 | Week 15 |  |  |
| Huntsville | 2024-04-14 | 104 | 16.94 | 23.61 | 10.28 | 2.22 | -2.22 | Week 15 |  |  |
| Huntsville | 2024-04-15 | 105 | 17.11 | 23.78 | 10.44 | 2.22 | 1.78 | Week 15 | -1.241 | 0.261 |
| Huntsville | 2024-04-16 | 106 | 17.28 | 23.94 | 10.61 | 2.22 | -3.11 | Week 16 |  |  |
| Huntsville | 2024-04-17 | 107 | 17.44 | 24.06 | 10.78 | 2.21 | -3.56 | Week 16 |  |  |
| Huntsville | 2024-04-18 | 108 | 17.61 | 24.22 | 10.94 | 2.21 | -1.22 | Week 16 |  |  |
| Huntsville | 2024-04-19 | 109 | 17.78 | 24.39 | 11.11 | 2.21 | 0.83 | Week 16 |  |  |
| Huntsville | 2024-04-20 | 110 | 17.89 | 24.56 | 11.28 | 2.21 | 2.67 | Week 16 |  |  |
| Huntsville | 2024-04-21 | 111 | 18.06 | 24.67 | 11.44 | 2.20 | 0.56 | Week 16 |  |  |
| Huntsville | 2024-04-22 | 112 | 18.22 | 24.83 | 11.67 | 2.19 | -2.67 | Week 16 | -1.047 | 0.335 |
| Huntsville | 2024-04-23 | 113 | 18.39 | 24.94 | 11.83 | 2.18 | -8.67 | Week 17 |  |  |
| Huntsville | 2024-04-24 | 114 | 18.56 | 25.11 | 12.00 | 2.18 | -6.89 | Week 17 |  |  |
| Huntsville | 2024-04-25 | 115 | 18.72 | 25.28 | 12.17 | 2.18 | -5.94 | Week 17 |  |  |
| Huntsville | 2024-04-26 | 116 | 18.89 | 25.39 | 12.33 | 2.17 | -4.72 | Week 17 |  |  |
| Huntsville | 2024-04-27 | 117 | 19.00 | 25.56 | 12.50 | 2.17 | -3.44 | Week 17 |  |  |
| Huntsville | 2024-04-28 | 118 | 19.17 | 25.67 | 12.67 | 2.16 | -1.94 | Week 17 |  |  |
| Huntsville | 2024-04-29 | 119 | 19.33 | 25.78 | 12.83 | 2.15 | -2.39 | **Week 17** | **-5.216** | **0.002** |
| Huntsville | 2024-04-30 | 120 | 19.50 | 25.94 | 13.00 | 2.15 | -5.33 | Week 18 |  |  |
| Huntsville | 2024-05-01 | 121 | 19.61 | 26.06 | 13.22 | 2.13 | -6.00 | Week 18 |  |  |
| Huntsville | 2024-05-02 | 122 | 19.78 | 26.22 | 13.39 | 2.13 | -5.89 | Week 18 |  |  |
| Huntsville | 2024-05-03 | 123 | 19.94 | 26.33 | 13.56 | 2.12 | -5.50 | Week 18 |  |  |
| Huntsville | 2024-05-04 | 124 | 20.11 | 26.44 | 13.72 | 2.11 | -4.56 | Week 18 |  |  |
| Huntsville | 2024-05-05 | 125 | 20.22 | 26.61 | 13.89 | 2.11 | -4.94 | Week 18 |  |  |
| Huntsville | 2024-05-06 | 126 | 20.39 | 26.72 | 14.06 | 2.10 | 0.72 | **Week 18** | **-5.050** | **0.002** |
| Huntsville | 2024-05-07 | 127 | 20.56 | 26.89 | 14.22 | 2.10 | 3.06 | Week 19 |  |  |
| Huntsville | 2024-05-08 | 128 | 20.67 | 27.00 | 14.39 | 2.10 | 2.94 | Week 19 |  |  |
| Huntsville | 2024-05-09 | 129 | 20.83 | 27.11 | 14.56 | 2.09 | 5.28 | Week 19 |  |  |
| Huntsville | 2024-05-10 | 130 | 21.00 | 27.28 | 14.72 | 2.09 | 2.61 | Week 19 |  |  |
| Huntsville | 2024-05-11 | 131 | 21.11 | 27.39 | 14.89 | 2.08 | 1.94 | Week 19 |  |  |
| Huntsville | 2024-05-12 | 132 | 21.28 | 27.50 | 15.06 | 2.07 | -0.44 | Week 19 |  |  |
| Huntsville | 2024-05-13 | 133 | 21.44 | 27.67 | 15.22 | 2.07 | 1.89 | **Week 19** | **3.815** | **0.009** |
| Huntsville | 2024-05-14 | 134 | 21.56 | 27.78 | 15.39 | 2.06 | 4.28 | Week 20 |  |  |
| Huntsville | 2024-05-15 | 135 | 21.72 | 27.89 | 15.56 | 2.05 | 4.94 | Week 20 |  |  |
| Huntsville | 2024-05-16 | 136 | 21.89 | 28.06 | 15.67 | 2.06 | 3.39 | Week 20 |  |  |
| Huntsville | 2024-05-17 | 137 | 22.00 | 28.17 | 15.83 | 2.05 | 1.06 | Week 20 |  |  |
| Huntsville | 2024-05-18 | 138 | 22.17 | 28.33 | 16.00 | 2.05 | 0.61 | Week 20 |  |  |
| Huntsville | 2024-05-19 | 139 | 22.33 | 28.44 | 16.17 | 2.04 | -1.50 | Week 20 |  |  |
| Huntsville | 2024-05-20 | 140 | 22.44 | 28.56 | 16.33 | 2.03 | -1.33 | Week 20 | 1.654 | 0.149 |
| Huntsville | 2024-05-21 | 141 | 22.61 | 28.72 | 16.50 | 2.03 | -1.50 | Week 21 |  |  |
| Huntsville | 2024-05-22 | 142 | 22.72 | 28.83 | 16.67 | 2.02 | -2.44 | Week 21 |  |  |
| Huntsville | 2024-05-23 | 143 | 22.89 | 29.00 | 16.83 | 2.02 | 0.44 | Week 21 |  |  |
| Huntsville | 2024-05-24 | 144 | 23.06 | 29.11 | 16.94 | 2.02 | -1.67 | Week 21 |  |  |
| Huntsville | 2024-05-25 | 145 | 23.17 | 29.28 | 17.11 | 2.02 | -1.22 | Week 21 |  |  |
| Huntsville | 2024-05-26 | 146 | 23.33 | 29.39 | 17.28 | 2.01 | -1.39 | Week 21 |  |  |
| Huntsville | 2024-05-27 | 147 | 23.50 | 29.50 | 17.44 | 2.00 | -1.00 | **Week 21** | **-3.782** | **0.009** |
| Huntsville | 2024-05-28 | 148 | 23.61 | 29.67 | 17.61 | 2.00 | -2.50 | Week 22 |  |  |
| Huntsville | 2024-05-29 | 149 | 23.78 | 29.78 | 17.72 | 2.00 | -4.61 | Week 22 |  |  |
| Huntsville | 2024-05-30 | 150 | 23.89 | 29.94 | 17.89 | 2.00 | -2.22 | Week 22 |  |  |
| Huntsville | 2024-05-31 | 151 | 24.06 | 30.06 | 18.06 | 1.99 | 0.39 | Week 22 |  |  |
| Huntsville | 2024-06-01 | 152 | 24.17 | 30.17 | 18.17 | 1.99 | 0.28 | Week 22 |  |  |
| Huntsville | 2024-06-02 | 153 | 24.33 | 30.33 | 18.33 | 1.99 | 1.22 | Week 22 |  |  |
| Huntsville | 2024-06-03 | 154 | 24.44 | 30.44 | 18.50 | 1.98 | 0.83 | Week 22 | -1.144 | 0.296 |
| Huntsville | 2024-06-04 | 155 | 24.61 | 30.56 | 18.61 | 1.98 | 6.78 | Week 23 |  |  |
| Huntsville | 2024-06-05 | 156 | 24.72 | 30.72 | 18.78 | 1.98 | 0.56 | Week 23 |  |  |
| Huntsville | 2024-06-06 | 157 | 24.89 | 30.83 | 18.89 | 1.98 | 0.28 | Week 23 |  |  |
| Huntsville | 2024-06-07 | 158 | 25.00 | 30.94 | 19.06 | 1.98 | -0.44 | Week 23 |  |  |
| Huntsville | 2024-06-08 | 159 | 25.11 | 31.06 | 19.17 | 1.98 | -0.67 | Week 23 |  |  |
| Huntsville | 2024-06-09 | 160 | 25.22 | 31.17 | 19.33 | 1.97 | -0.67 | Week 23 |  |  |
| Huntsville | 2024-06-10 | 161 | 25.39 | 31.28 | 19.44 | 1.97 | -1.00 | Week 23 | 0.667 | 0.530 |
| Huntsville | 2024-06-11 | 162 | 25.50 | 31.39 | 19.56 | 1.97 | 0.22 | Week 24 |  |  |
| Huntsville | 2024-06-12 | 163 | 25.61 | 31.50 | 19.72 | 1.96 | -3.83 | Week 24 |  |  |
| Huntsville | 2024-06-13 | 164 | 25.72 | 31.61 | 19.83 | 1.96 | -3.50 | Week 24 |  |  |
| Huntsville | 2024-06-14 | 165 | 25.83 | 31.72 | 19.94 | 1.96 | -3.78 | Week 24 |  |  |
| Huntsville | 2024-06-15 | 166 | 25.94 | 31.83 | 20.06 | 1.96 | -3.22 | Week 24 |  |  |
| Huntsville | 2024-06-16 | 167 | 26.06 | 31.89 | 20.17 | 1.95 | -3.22 | Week 24 |  |  |
| Huntsville | 2024-06-17 | 168 | 26.17 | 32.00 | 20.28 | 1.95 | -0.06 | **Week 24** | **-3.709** | **0.010** |
| Huntsville | 2024-06-18 | 169 | 26.22 | 32.06 | 20.39 | 1.94 | -2.17 | Week 25 |  |  |
| Huntsville | 2024-06-19 | 170 | 26.33 | 32.17 | 20.50 | 1.94 | -2.33 | Week 25 |  |  |
| Huntsville | 2024-06-20 | 171 | 26.44 | 32.22 | 20.61 | 1.93 | -2.67 | Week 25 |  |  |
| Huntsville | 2024-06-21 | 172 | 26.50 | 32.33 | 20.72 | 1.93 | -4.33 | Week 25 |  |  |
| Huntsville | 2024-06-22 | 173 | 26.61 | 32.39 | 20.78 | 1.93 | -5.28 | Week 25 |  |  |
| Huntsville | 2024-06-23 | 174 | 26.67 | 32.44 | 20.89 | 1.92 | -2.94 | Week 25 |  |  |
| Huntsville | 2024-06-24 | 175 | 26.72 | 32.50 | 20.94 | 1.92 | -0.50 | **Week 25** | **-4.937** | **0.003** |
| Huntsville | 2024-06-25 | 176 | 26.78 | 32.56 | 21.06 | 1.91 | -2.11 | Week 26 |  |  |
| Huntsville | 2024-06-26 | 177 | 26.89 | 32.61 | 21.11 | 1.91 | -0.78 | Week 26 |  |  |
| Huntsville | 2024-06-27 | 178 | 26.94 | 32.67 | 21.17 | 1.91 | -0.94 | Week 26 |  |  |
| Huntsville | 2024-06-28 | 179 | 27.00 | 32.72 | 21.28 | 1.90 | -0.33 | Week 26 |  |  |
| Huntsville | 2024-06-29 | 180 | 27.06 | 32.78 | 21.33 | 1.90 | -0.44 | Week 26 |  |  |
| Huntsville | 2024-06-30 | 181 | 27.11 | 32.78 | 21.39 | 1.89 | 1.56 | Week 26 |  |  |
| Huntsville | 2024-07-01 | 182 | 27.11 | 32.83 | 21.44 | 1.89 | -2.11 | Week 26 | -1.566 | 0.168 |
| Huntsville | 2024-07-02 | 183 | 27.17 | 32.89 | 21.50 | 1.89 | 0.50 | Week 27 |  |  |
| Huntsville | 2024-07-03 | 184 | 27.22 | 32.89 | 21.56 | 1.88 | -3.22 | Week 27 |  |  |
| Huntsville | 2024-07-04 | 185 | 27.22 | 32.94 | 21.56 | 1.89 | -1.83 | Week 27 |  |  |
| Huntsville | 2024-07-05 | 186 | 27.28 | 32.94 | 21.61 | 1.88 | -0.22 | Week 27 |  |  |
| Huntsville | 2024-07-06 | 187 | 27.33 | 32.94 | 21.67 | 1.87 | -1.00 | Week 27 |  |  |
| Huntsville | 2024-07-07 | 188 | 27.33 | 33.00 | 21.67 | 1.88 | -1.22 | Week 27 |  |  |
| Huntsville | 2024-07-08 | 189 | 27.33 | 33.00 | 21.72 | 1.87 | -1.72 | **Week 27** | **-2.748** | **0.033** |
| Huntsville | 2024-07-09 | 190 | 27.39 | 33.00 | 21.72 | 1.87 | -2.78 | Week 28 |  |  |
| Huntsville | 2024-07-10 | 191 | 27.39 | 33.06 | 21.78 | 1.87 | -2.33 | Week 28 |  |  |
| Huntsville | 2024-07-11 | 192 | 27.39 | 33.06 | 21.78 | 1.87 | -4.78 | Week 28 |  |  |
| Huntsville | 2024-07-12 | 193 | 27.44 | 33.06 | 21.78 | 1.87 | 0.22 | Week 28 |  |  |
| Huntsville | 2024-07-13 | 194 | 27.44 | 33.06 | 21.78 | 1.87 | -0.39 | Week 28 |  |  |
| Huntsville | 2024-07-14 | 195 | 27.44 | 33.06 | 21.83 | 1.86 | -1.44 | Week 28 |  |  |
| Huntsville | 2024-07-15 | 196 | 27.44 | 33.06 | 21.83 | 1.86 | -2.06 | **Week 28** | **-3.113** | **0.021** |
| Huntsville | 2024-07-16 | 197 | 27.44 | 33.11 | 21.83 | 1.87 | -0.67 | Week 29 |  |  |
| Huntsville | 2024-07-17 | 198 | 27.44 | 33.11 | 21.83 | 1.87 | -1.56 | Week 29 |  |  |
| Huntsville | 2024-07-18 | 199 | 27.44 | 33.11 | 21.83 | 1.87 | -1.94 | Week 29 |  |  |
| Huntsville | 2024-07-19 | 200 | 27.44 | 33.11 | 21.83 | 1.87 | -4.28 | Week 29 |  |  |
| Huntsville | 2024-07-20 | 201 | 27.44 | 33.11 | 21.83 | 1.87 | 0.61 | Week 29 |  |  |
| Huntsville | 2024-07-21 | 202 | 27.44 | 33.11 | 21.83 | 1.87 | -2.61 | Week 29 |  |  |
| Huntsville | 2024-07-22 | 203 | 27.44 | 33.11 | 21.78 | 1.88 | -3.22 | **Week 29** | **-3.180** | **0.019** |
| Huntsville | 2024-07-23 | 204 | 27.44 | 33.11 | 21.78 | 1.88 | -2.39 | Week 30 |  |  |
| Huntsville | 2024-07-24 | 205 | 27.44 | 33.11 | 21.78 | 1.88 | -2.06 | Week 30 |  |  |
| Huntsville | 2024-07-25 | 206 | 27.44 | 33.11 | 21.78 | 1.88 | -0.44 | Week 30 |  |  |
| Huntsville | 2024-07-26 | 207 | 27.44 | 33.11 | 21.72 | 1.89 | 0.39 | Week 30 |  |  |
| Huntsville | 2024-07-27 | 208 | 27.44 | 33.11 | 21.72 | 1.89 | 1.78 | Week 30 |  |  |
| Huntsville | 2024-07-28 | 209 | 27.44 | 33.17 | 21.72 | 1.90 | 1.06 | Week 30 |  |  |
| Huntsville | 2024-07-29 | 210 | 27.44 | 33.17 | 21.72 | 1.90 | 1.67 | Week 30 | <0.001 | 1.000 |
| Huntsville | 2024-07-30 | 211 | 27.39 | 33.17 | 21.67 | 1.91 | -0.28 | Week 31 |  |  |
| Huntsville | 2024-07-31 | 212 | 27.39 | 33.17 | 21.67 | 1.91 | 0.17 | Week 31 |  |  |
| Huntsville | 2024-08-01 | 213 | 27.39 | 33.17 | 21.61 | 1.92 | 0.06 | Week 31 |  |  |
| Huntsville | 2024-08-02 | 214 | 27.39 | 33.17 | 21.61 | 1.92 | -1.56 | Week 31 |  |  |
| Huntsville | 2024-08-03 | 215 | 27.39 | 33.17 | 21.56 | 1.93 | -2.39 | Week 31 |  |  |
| Huntsville | 2024-08-04 | 216 | 27.33 | 33.17 | 21.56 | 1.93 | -3.11 | Week 31 |  |  |
| Huntsville | 2024-08-05 | 217 | 27.33 | 33.17 | 21.50 | 1.94 | -2.61 | **Week 31** | **-2.686** | **0.036** |
| Huntsville | 2024-08-06 | 218 | 27.33 | 33.17 | 21.50 | 1.94 | -1.94 | Week 32 |  |  |
| Huntsville | 2024-08-07 | 219 | 27.33 | 33.17 | 21.44 | 1.95 | -3.00 | Week 32 |  |  |
| Huntsville | 2024-08-08 | 220 | 27.28 | 33.17 | 21.44 | 1.95 | -4.50 | Week 32 |  |  |
| Huntsville | 2024-08-09 | 221 | 27.28 | 33.17 | 21.39 | 1.96 | -4.72 | Week 32 |  |  |
| Huntsville | 2024-08-10 | 222 | 27.22 | 33.17 | 21.33 | 1.97 | -3.50 | Week 32 |  |  |
| Huntsville | 2024-08-11 | 223 | 27.22 | 33.17 | 21.28 | 1.98 | -0.11 | Week 32 |  |  |
| Huntsville | 2024-08-12 | 224 | 27.22 | 33.11 | 21.28 | 1.97 | 0.17 | **Week 32** | **-3.377** | **0.015** |
| Huntsville | 2024-08-13 | 225 | 27.17 | 33.11 | 21.22 | 1.98 | -1.22 | Week 33 |  |  |
| Huntsville | 2024-08-14 | 226 | 27.11 | 33.11 | 21.17 | 1.98 | 0.61 | Week 33 |  |  |
| Huntsville | 2024-08-15 | 227 | 27.11 | 33.11 | 21.11 | 1.99 | -2.50 | Week 33 |  |  |
| Huntsville | 2024-08-16 | 228 | 27.06 | 33.06 | 21.06 | 1.99 | -4.11 | Week 33 |  |  |
| Huntsville | 2024-08-17 | 229 | 27.00 | 33.06 | 21.00 | 2.00 | -5.33 | Week 33 |  |  |
| Huntsville | 2024-08-18 | 230 | 27.00 | 33.00 | 20.94 | 2.00 | -2.06 | Week 33 |  |  |
| Huntsville | 2024-08-19 | 231 | 26.94 | 33.00 | 20.89 | 2.01 | -2.44 | **Week 33** | **-3.358** | **0.015** |
| Huntsville | 2024-08-20 | 232 | 26.89 | 32.94 | 20.83 | 2.01 | -0.56 | Week 34 |  |  |
| Huntsville | 2024-08-21 | 233 | 26.83 | 32.89 | 20.72 | 2.02 | 1.22 | Week 34 |  |  |
| Huntsville | 2024-08-22 | 234 | 26.78 | 32.89 | 20.67 | 2.03 | 2.06 | Week 34 |  |  |
| Huntsville | 2024-08-23 | 235 | 26.72 | 32.83 | 20.61 | 2.03 | 2.83 | Week 34 |  |  |
| Huntsville | 2024-08-24 | 236 | 26.67 | 32.78 | 20.50 | 2.04 | 2.56 | Week 34 |  |  |
| Huntsville | 2024-08-25 | 237 | 26.56 | 32.72 | 20.44 | 2.04 | 3.22 | Week 34 |  |  |
| Huntsville | 2024-08-26 | 238 | 26.50 | 32.67 | 20.33 | 2.05 | 1.06 | **Week 34** | **3.599** | **0.011** |
| Huntsville | 2024-08-27 | 239 | 26.44 | 32.61 | 20.22 | 2.06 | 1.17 | Week 35 |  |  |
| Huntsville | 2024-08-28 | 240 | 26.33 | 32.50 | 20.17 | 2.05 | -1.11 | Week 35 |  |  |
| Huntsville | 2024-08-29 | 241 | 26.28 | 32.44 | 20.06 | 2.06 | -0.17 | Week 35 |  |  |
| Huntsville | 2024-08-30 | 242 | 26.17 | 32.39 | 19.94 | 2.07 | -1.06 | Week 35 |  |  |
| Huntsville | 2024-08-31 | 243 | 26.06 | 32.28 | 19.83 | 2.07 | -2.89 | Week 35 |  |  |
| Huntsville | 2024-09-01 | 244 | 25.94 | 32.22 | 19.72 | 2.08 | -0.67 | Week 35 |  |  |
| Huntsville | 2024-09-02 | 245 | 25.83 | 32.11 | 19.61 | 2.08 | -3.33 | Week 35 | -1.968 | 0.097 |
| Huntsville | 2024-09-03 | 246 | 25.72 | 32.00 | 19.50 | 2.08 | -1.11 | Week 36 |  |  |
| Huntsville | 2024-09-04 | 247 | 25.61 | 31.89 | 19.33 | 2.09 | 0.44 | Week 36 |  |  |
| Huntsville | 2024-09-05 | 248 | 25.50 | 31.78 | 19.22 | 2.09 | 1.06 | Week 36 |  |  |
| Huntsville | 2024-09-06 | 249 | 25.39 | 31.67 | 19.11 | 2.09 | 0.61 | Week 36 |  |  |
| Huntsville | 2024-09-07 | 250 | 25.28 | 31.56 | 18.94 | 2.10 | -2.33 | Week 36 |  |  |
| Huntsville | 2024-09-08 | 251 | 25.11 | 31.44 | 18.78 | 2.10 | 0.00 | Week 36 |  |  |
| Huntsville | 2024-09-09 | 252 | 25.00 | 31.33 | 18.67 | 2.10 | -0.94 | Week 36 | -0.723 | 0.497 |
| Huntsville | 2024-09-10 | 253 | 24.83 | 31.22 | 18.50 | 2.11 | -1.61 | Week 37 |  |  |
| Huntsville | 2024-09-11 | 254 | 24.72 | 31.06 | 18.33 | 2.11 | -0.17 | Week 37 |  |  |
| Huntsville | 2024-09-12 | 255 | 24.56 | 30.94 | 18.17 | 2.12 | 0.17 | Week 37 |  |  |
| Huntsville | 2024-09-13 | 256 | 24.39 | 30.78 | 18.00 | 2.12 | -0.61 | Week 37 |  |  |
| Huntsville | 2024-09-14 | 257 | 24.22 | 30.67 | 17.83 | 2.13 | -1.72 | Week 37 |  |  |
| Huntsville | 2024-09-15 | 258 | 24.06 | 30.50 | 17.67 | 2.13 | -0.67 | Week 37 |  |  |
| Huntsville | 2024-09-16 | 259 | 23.94 | 30.33 | 17.50 | 2.13 | -1.67 | **Week 37** | **-3.071** | **0.022** |
| Huntsville | 2024-09-17 | 260 | 23.72 | 30.17 | 17.33 | 2.13 | -3.06 | Week 38 |  |  |
| Huntsville | 2024-09-18 | 261 | 23.56 | 30.00 | 17.11 | 2.14 | -3.39 | Week 38 |  |  |
| Huntsville | 2024-09-19 | 262 | 23.39 | 29.83 | 16.94 | 2.14 | -3.22 | Week 38 |  |  |
| Huntsville | 2024-09-20 | 263 | 23.22 | 29.67 | 16.72 | 2.15 | -1.28 | Week 38 |  |  |
| Huntsville | 2024-09-21 | 264 | 23.06 | 29.50 | 16.56 | 2.15 | -0.56 | Week 38 |  |  |
| Huntsville | 2024-09-22 | 265 | 22.83 | 29.33 | 16.33 | 2.16 | -1.17 | Week 38 |  |  |
| Huntsville | 2024-09-23 | 266 | 22.67 | 29.17 | 16.17 | 2.16 | -2.17 | **Week 38** | **-4.927** | **0.003** |
| Huntsville | 2024-09-24 | 267 | 22.44 | 29.00 | 15.94 | 2.17 | -1.00 | Week 39 |  |  |
| Huntsville | 2024-09-25 | 268 | 22.28 | 28.83 | 15.72 | 2.18 | 0.56 | Week 39 |  |  |
| Huntsville | 2024-09-26 | 269 | 22.06 | 28.61 | 15.56 | 2.17 | 1.50 | Week 39 |  |  |
| Huntsville | 2024-09-27 | 270 | 21.89 | 28.44 | 15.33 | 2.18 | 2.06 | Week 39 |  |  |
| Huntsville | 2024-09-28 | 271 | 21.67 | 28.22 | 15.11 | 2.18 | 0.56 | Week 39 |  |  |
| Huntsville | 2024-09-29 | 272 | 21.44 | 28.06 | 14.89 | 2.19 | -0.33 | Week 39 |  |  |
| Huntsville | 2024-09-30 | 273 | 21.28 | 27.83 | 14.67 | 2.19 | -0.33 | Week 39 |  |  |
| Huntsville | 2024-10-01 | 274 | 21.06 | 27.67 | 14.44 | 2.20 | 2.89 | Week 40 |  |  |
| Huntsville | 2024-10-02 | 275 | 20.83 | 27.44 | 14.22 | 2.20 | 2.67 | Week 40 |  |  |
| Huntsville | 2024-10-03 | 276 | 20.61 | 27.28 | 14.00 | 2.21 | 1.89 | Week 40 |  |  |
| Huntsville | 2024-10-04 | 277 | 20.39 | 27.06 | 13.78 | 2.21 | 1.83 | Week 40 |  |  |
| Huntsville | 2024-10-05 | 278 | 20.22 | 26.83 | 13.56 | 2.21 | 2.28 | Week 40 |  |  |
| Huntsville | 2024-10-06 | 279 | 20.00 | 26.67 | 13.33 | 2.22 | -0.11 | Week 40 |  |  |
| Huntsville | 2024-10-07 | 280 | 19.78 | 26.44 | 13.06 | 2.22 | -4.83 | Week 40 | 0.915 | 0.395 |
| Huntsville | 2024-10-08 | 281 | 19.56 | 26.22 | 12.83 | 2.22 | -5.83 | Week 41 |  |  |
| Huntsville | 2024-10-09 | 282 | 19.33 | 26.00 | 12.61 | 2.22 | -0.11 | Week 41 |  |  |
| Huntsville | 2024-10-10 | 283 | 19.11 | 25.78 | 12.39 | 2.22 | 0.06 | Week 41 |  |  |
| Huntsville | 2024-10-11 | 284 | 18.89 | 25.61 | 12.17 | 2.23 | -1.22 | Week 41 |  |  |
| Huntsville | 2024-10-12 | 285 | 18.67 | 25.39 | 11.89 | 2.24 | 1.06 | Week 41 |  |  |
| Huntsville | 2024-10-13 | 286 | 18.44 | 25.17 | 11.67 | 2.24 | 0.39 | Week 41 |  |  |
| Huntsville | 2024-10-14 | 287 | 18.17 | 24.94 | 11.44 | 2.24 | 0.56 | Week 41 | -0.819 | 0.444 |
| Huntsville | 2024-10-15 | 288 | 17.94 | 24.72 | 11.22 | 2.24 | -5.44 | Week 42 |  |  |
| Huntsville | 2024-10-16 | 289 | 17.72 | 24.50 | 11.00 | 2.24 | -5.89 | Week 42 |  |  |
| Huntsville | 2024-10-17 | 290 | 17.50 | 24.28 | 10.72 | 2.25 | -4.94 | Week 42 |  |  |
| Huntsville | 2024-10-18 | 291 | 17.28 | 24.06 | 10.50 | 2.25 | -4.50 | Week 42 |  |  |
| Huntsville | 2024-10-19 | 292 | 17.06 | 23.83 | 10.28 | 2.25 | -0.50 | Week 42 |  |  |
| Huntsville | 2024-10-20 | 293 | 16.83 | 23.61 | 10.06 | 2.25 | 1.72 | Week 42 |  |  |
| Huntsville | 2024-10-21 | 294 | 16.61 | 23.39 | 9.83 | 2.25 | 0.44 | Week 42 | -2.279 | 0.063 |
| Huntsville | 2024-10-22 | 295 | 16.39 | 23.11 | 9.61 | 2.24 | 1.17 | Week 43 |  |  |
| Huntsville | 2024-10-23 | 296 | 16.11 | 22.89 | 9.39 | 2.24 | 0.39 | Week 43 |  |  |
| Huntsville | 2024-10-24 | 297 | 15.89 | 22.67 | 9.17 | 2.24 | 3.22 | Week 43 |  |  |
| Huntsville | 2024-10-25 | 298 | 15.67 | 22.44 | 8.94 | 2.24 | 3.78 | Week 43 |  |  |
| Huntsville | 2024-10-26 | 299 | 15.44 | 22.22 | 8.72 | 2.24 | 6.22 | Week 43 |  |  |
| Huntsville | 2024-10-27 | 300 | 15.22 | 22.00 | 8.50 | 2.24 | 6.72 | Week 43 |  |  |
| Huntsville | 2024-10-28 | 301 | 15.00 | 21.78 | 8.28 | 2.24 | 7.28 | **Week 43** | **3.984** | **0.007** |
| Huntsville | 2024-10-29 | 302 | 14.78 | 21.50 | 8.06 | 2.23 | 6.83 | Week 44 |  |  |
| Huntsville | 2024-10-30 | 303 | 14.56 | 21.28 | 7.83 | 2.23 | -2.56 | Week 44 |  |  |
| Huntsville | 2024-10-31 | 304 | 14.33 | 21.06 | 7.61 | 2.23 | -6.56 | Week 44 | -0.191 | 0.866 |
| Holden | 2024-01-01 | 1 | -2.17 | 1.56 | -5.83 | 6.11 | 8.28 | Week 1 |  |  |
| Holden | 2024-01-02 | 2 | -2.22 | 1.44 | -5.94 | 8.89 | 11.11 | Week 1 |  |  |
| Holden | 2024-01-03 | 3 | -2.33 | 1.39 | -6.06 | 13.06 | 15.39 | Week 1 |  |  |
| Holden | 2024-01-04 | 4 | -2.44 | 1.28 | -6.17 | 8.89 | 11.33 | Week 1 |  |  |
| Holden | 2024-01-05 | 5 | -2.56 | 1.22 | -6.28 | 5.28 | 7.83 | Week 1 |  |  |
| Holden | 2024-01-06 | 6 | -2.61 | 1.17 | -6.39 | 1.94 | 4.56 | Week 1 |  |  |
| Holden | 2024-01-07 | 7 | -2.72 | 1.06 | -6.5 | 0.56 | 3.28 | **Week 1** | **5.585** | **0.001** |
| Holden | 2024-01-08 | 8 | -2.78 | 1 | -6.61 | 0.56 | 3.33 | Week 2 |  |  |
| Holden | 2024-01-09 | 9 | -2.89 | 0.94 | -6.67 | 0.56 | 3.44 | Week 2 |  |  |
| Holden | 2024-01-10 | 10 | -2.94 | 0.89 | -6.78 | 2.78 | 5.72 | Week 2 |  |  |
| Holden | 2024-01-11 | 11 | -3 | 0.89 | -6.89 | 5 | 8 | Week 2 |  |  |
| Holden | 2024-01-12 | 12 | -3.06 | 0.83 | -6.94 | 5.28 | 8.33 | Week 2 |  |  |
| Holden | 2024-01-13 | 13 | -3.11 | 0.78 | -7.06 | 0.28 | 3.39 | Week 2 |  |  |
| Holden | 2024-01-14 | 14 | -3.17 | 0.72 | -7.11 | -3.89 | -0.72 | **Week 2** | **3.783** | **0.009** |
| Holden | 2024-01-15 | 15 | -3.22 | 0.72 | -7.22 | -3.33 | -0.11 | Week 3 |  |  |
| Holden | 2024-01-16 | 16 | -3.28 | 0.67 | -7.28 | 1.11 | 4.39 | Week 3 |  |  |
| Holden | 2024-01-17 | 17 | -3.33 | 0.67 | -7.33 | 8.33 | 11.67 | Week 3 |  |  |
| Holden | 2024-01-18 | 18 | -3.39 | 0.67 | -7.44 | 4.44 | 7.83 | Week 3 |  |  |
| Holden | 2024-01-19 | 19 | -3.39 | 0.67 | -7.5 | 8.89 | 12.28 | Week 3 |  |  |
| Holden | 2024-01-20 | 20 | -3.44 | 0.67 | -7.56 | 2.78 | 6.22 | Week 3 |  |  |
| Holden | 2024-01-21 | 21 | -3.44 | 0.67 | -7.56 | 0.83 | 4.28 | **Week 3** | **4.021** | **0.007** |
| Holden | 2024-01-22 | 22 | -3.5 | 0.67 | -7.61 | 0 | 3.5 | Week 4 |  |  |
| Holden | 2024-01-23 | 23 | -3.5 | 0.67 | -7.67 | -0.56 | 2.94 | Week 4 |  |  |
| Holden | 2024-01-24 | 24 | -3.5 | 0.67 | -7.72 | 0 | 3.5 | Week 4 |  |  |
| Holden | 2024-01-25 | 25 | -3.5 | 0.72 | -7.72 | 3.33 | 6.83 | Week 4 |  |  |
| Holden | 2024-01-26 | 26 | -3.5 | 0.72 | -7.78 | -0.83 | 2.67 | Week 4 |  |  |
| Holden | 2024-01-27 | 27 | -3.5 | 0.78 | -7.78 | -0.56 | 2.94 | Week 4 |  |  |
| Holden | 2024-01-28 | 28 | -3.5 | 0.78 | -7.78 | 1.39 | 4.89 | **Week 4** | **6.938** | **<0.001** |
| Holden | 2024-01-29 | 29 | -3.44 | 0.83 | -7.78 | 3.33 | 6.78 | Week 5 |  |  |
| Holden | 2024-01-30 | 30 | -3.44 | 0.89 | -7.78 | -1.94 | 1.5 | Week 5 |  |  |
| Holden | 2024-01-31 | 31 | -3.39 | 0.94 | -7.78 | -6.39 | -3 | Week 5 |  |  |
| Holden | 2024-02-01 | 32 | -3.39 | 1 | -7.72 | -6.11 | -2.72 | Week 5 |  |  |
| Holden | 2024-02-02 | 33 | -3.33 | 1.06 | -7.72 | -1.11 | 2.22 | Week 5 |  |  |
| Holden | 2024-02-03 | 34 | -3.28 | 1.17 | -7.67 | -7.78 | -4.5 | Week 5 |  |  |
| Holden | 2024-02-04 | 35 | -3.22 | 1.22 | -7.67 | -4.17 | -0.94 | Week 5 | -0.065 | 0.95 |
| Holden | 2024-02-05 | 36 | -3.17 | 1.28 | -7.61 | 5.83 | 9 | Week 6 |  |  |
| Holden | 2024-02-06 | 37 | -3.11 | 1.39 | -7.56 | 2.22 | 5.33 | Week 6 |  |  |
| Holden | 2024-02-07 | 38 | -3 | 1.44 | -7.5 | 6.67 | 9.67 | Week 6 |  |  |
| Holden | 2024-02-08 | 39 | -2.94 | 1.56 | -7.44 | 5 | 7.94 | Week 6 |  |  |
| Holden | 2024-02-09 | 40 | -2.83 | 1.67 | -7.33 | 12.5 | 15.33 | Week 6 |  |  |
| Holden | 2024-02-10 | 41 | -2.78 | 1.78 | -7.28 | 2.22 | 5 | Week 6 |  |  |
| Holden | 2024-02-11 | 42 | -2.67 | 1.89 | -7.17 | 1.11 | 3.78 | **Week 6** | **5.426** | **0.002** |
| Holden | 2024-02-12 | 43 | -2.56 | 2 | -7.11 | 3.61 | 6.17 | Week 7 |  |  |
| Holden | 2024-02-13 | 44 | -2.44 | 2.11 | -7 | 6.11 | 8.56 | Week 7 |  |  |
| Holden | 2024-02-14 | 45 | -2.33 | 2.22 | -6.89 | 4.72 | 7.06 | Week 7 |  |  |
| Holden | 2024-02-15 | 46 | -2.22 | 2.33 | -6.78 | 13.61 | 15.83 | Week 7 |  |  |
| Holden | 2024-02-16 | 47 | -2.11 | 2.44 | -6.67 | 9.17 | 11.28 | Week 7 |  |  |
| Holden | 2024-02-17 | 48 | -2 | 2.61 | -6.56 | 5 | 7 | Week 7 |  |  |
| Holden | 2024-02-18 | 49 | -1.83 | 2.72 | -6.44 | 1.11 | 2.94 | **Week 7** | **5.391** | **0.002** |
| Holden | 2024-02-19 | 50 | -1.72 | 2.83 | -6.28 | 8.33 | 10.06 | Week 8 |  |  |
| Holden | 2024-02-20 | 51 | -1.61 | 3 | -6.17 | 5.28 | 6.89 | Week 8 |  |  |
| Holden | 2024-02-21 | 52 | -1.44 | 3.11 | -6.06 | 4.44 | 5.89 | Week 8 |  |  |
| Holden | 2024-02-22 | 53 | -1.33 | 3.28 | -5.89 | 1.39 | 2.72 | Week 8 |  |  |
| Holden | 2024-02-23 | 54 | -1.17 | 3.44 | -5.72 | 10 | 11.17 | Week 8 |  |  |
| Holden | 2024-02-24 | 55 | -1 | 3.56 | -5.61 | -0.83 | 0.17 | Week 8 |  |  |
| Holden | 2024-02-25 | 56 | -0.89 | 3.72 | -5.44 | 0.28 | 1.17 | **Week 8** | **3.362** | **0.015** |
| Holden | 2024-02-26 | 57 | -0.72 | 3.89 | -5.28 | 3.89 | 4.61 | Week 9 |  |  |
| Holden | 2024-02-27 | 58 | -0.56 | 4 | -5.11 | 4.17 | 4.72 | Week 9 |  |  |
| Holden | 2024-02-28 | 59 | -0.39 | 4.17 | -4.94 | 4.72 | 5.11 | Week 9 |  |  |
| Holden | 2024-03-01 | 60 | -0.22 | 4.33 | -4.78 | 8.61 | 8.83 | Week 9 |  |  |
| Holden | 2024-03-02 | 61 | -0.06 | 4.5 | -4.67 | 4.72 | 4.78 | Week 9 |  |  |
| Holden | 2024-03-03 | 62 | 0.11 | 4.67 | -4.5 | 5.56 | 5.44 | Week 9 |  |  |
| Holden | 2024-03-04 | 63 | 0.28 | 4.83 | -4.33 | 5.83 | 5.56 | **Week 9** | **9.975** | **<0.001** |
| Holden | 2024-03-05 | 64 | 0.44 | 5 | -4.17 | 6.11 | 5.67 | Week 10 |  |  |
| Holden | 2024-03-06 | 65 | 0.61 | 5.17 | -3.94 | 7.78 | 7.17 | Week 10 |  |  |
| Holden | 2024-03-07 | 66 | 0.78 | 5.33 | -3.78 | 1.94 | 1.17 | Week 10 |  |  |
| Holden | 2024-03-08 | 67 | 0.94 | 5.5 | -3.61 | 1.67 | 0.72 | Week 10 |  |  |
| Holden | 2024-03-09 | 68 | 1.11 | 5.67 | -3.44 | 0.56 | -0.56 | Week 10 |  |  |
| Holden | 2024-03-10 | 69 | 1.28 | 5.83 | -3.28 | 1.39 | 0.11 | Week 10 |  |  |
| Holden | 2024-03-11 | 70 | 1.44 | 6.06 | -3.11 | 0 | -1.44 | Week 10 | 1.482 | 0.189 |
| Holden | 2024-03-12 | 71 | 1.67 | 6.22 | -2.94 | 0.83 | -0.83 | Week 11 |  |  |
| Holden | 2024-03-13 | 72 | 1.83 | 6.39 | -2.78 | 0 | -1.83 | Week 11 |  |  |
| Holden | 2024-03-14 | 73 | 2 | 6.61 | -2.61 | -3.89 | -5.89 | Week 11 |  |  |
| Holden | 2024-03-15 | 74 | 2.17 | 6.78 | -2.39 | -0.56 | -2.72 | Week 11 |  |  |
| Holden | 2024-03-16 | 75 | 2.39 | 7 | -2.22 | 8.06 | 5.67 | Week 11 |  |  |
| Holden | 2024-03-17 | 76 | 2.56 | 7.17 | -2.06 | 6.94 | 4.39 | Week 11 |  |  |
| Holden | 2024-03-18 | 77 | 2.78 | 7.39 | -1.89 | -2.5 | -5.28 | Week 11 | -0.551 | 0.602 |
| Holden | 2024-03-19 | 78 | 2.94 | 7.61 | -1.72 | -1.94 | -4.89 | Week 12 |  |  |
| Holden | 2024-03-20 | 79 | 3.11 | 7.78 | -1.56 | 3.61 | 0.5 | Week 12 |  |  |
| Holden | 2024-03-21 | 80 | 3.33 | 8 | -1.39 | 8.33 | 5 | Week 12 |  |  |
| Holden | 2024-03-22 | 81 | 3.5 | 8.22 | -1.17 | 9.17 | 5.67 | Week 12 |  |  |
| Holden | 2024-03-23 | 82 | 3.72 | 8.44 | -1 | 8.89 | 5.17 | Week 12 |  |  |
| Holden | 2024-03-24 | 83 | 3.94 | 8.67 | -0.83 | 3.61 | -0.33 | Week 12 |  |  |
| Holden | 2024-03-25 | 84 | 4.11 | 8.89 | -0.67 | 10 | 5.89 | Week 12 | 1.563 | 0.169 |
| Holden | 2024-03-26 | 85 | 4.33 | 9.11 | -0.44 | 8.06 | 3.72 | Week 13 |  |  |
| Holden | 2024-03-27 | 86 | 4.56 | 9.33 | -0.28 | 6.94 | 2.39 | Week 13 |  |  |
| Holden | 2024-03-28 | 87 | 4.72 | 9.61 | -0.11 | 4.17 | -0.56 | Week 13 |  |  |
| Holden | 2024-03-29 | 88 | 4.94 | 9.83 | 0.06 | 5 | 0.06 | Week 13 |  |  |
| Holden | 2024-03-30 | 89 | 5.17 | 10.06 | 0.28 | 2.5 | -2.67 | Week 13 |  |  |
| Holden | 2024-03-31 | 90 | 5.39 | 10.33 | 0.44 | 10.28 | 4.89 | Week 13 |  |  |
| Holden | 2024-04-01 | 91 | 5.61 | 10.56 | 0.61 | 9.44 | 3.83 | Week 13 | 1.59 | 0.163 |
| Holden | 2024-04-02 | 92 | 5.83 | 10.83 | 0.83 | 3.33 | -2.5 | Week 14 |  |  |
| Holden | 2024-04-03 | 93 | 6.06 | 11.06 | 1 | 12.22 | 6.17 | Week 14 |  |  |
| Holden | 2024-04-04 | 94 | 6.28 | 11.33 | 1.17 | 15 | 8.72 | Week 14 |  |  |
| Holden | 2024-04-05 | 95 | 6.5 | 11.56 | 1.39 | 20.83 | 14.33 | Week 14 |  |  |
| Holden | 2024-04-06 | 96 | 6.72 | 11.83 | 1.56 | 10.83 | 4.11 | Week 14 |  |  |
| Holden | 2024-04-07 | 97 | 6.94 | 12.11 | 1.78 | 3.61 | -3.33 | Week 14 |  |  |
| Holden | 2024-04-08 | 98 | 7.17 | 12.33 | 1.94 | 5.83 | -1.33 | Week 14 | 1.509 | 0.182 |
| Holden | 2024-04-09 | 99 | 7.39 | 12.61 | 2.17 | 6.67 | -0.72 | Week 15 |  |  |
| Holden | 2024-04-10 | 100 | 7.61 | 12.89 | 2.33 | 8.89 | 1.28 | Week 15 |  |  |
| Holden | 2024-04-11 | 101 | 7.83 | 13.11 | 2.56 | 13.89 | 6.06 | Week 15 |  |  |
| Holden | 2024-04-12 | 102 | 8.06 | 13.39 | 2.78 | 20.56 | 12.5 | Week 15 |  |  |
| Holden | 2024-04-13 | 103 | 8.28 | 13.67 | 2.94 | 21.11 | 12.83 | Week 15 |  |  |
| Holden | 2024-04-14 | 104 | 8.56 | 13.89 | 3.17 | 18.89 | 10.33 | Week 15 |  |  |
| Holden | 2024-04-15 | 105 | 8.78 | 14.17 | 3.39 | 20.56 | 11.78 | **Week 15** | **3.652** | **0.011** |
| Holden | 2024-04-16 | 106 | 9 | 14.39 | 3.56 | 16.94 | 7.94 | Week 16 |  |  |
| Holden | 2024-04-17 | 107 | 9.22 | 14.67 | 3.78 | 5.28 | -3.94 | Week 16 |  |  |
| Holden | 2024-04-18 | 108 | 9.44 | 14.94 | 4 | 5.83 | -3.61 | Week 16 |  |  |
| Holden | 2024-04-19 | 109 | 9.67 | 15.17 | 4.17 | 6.39 | -3.28 | Week 16 |  |  |
| Holden | 2024-04-20 | 110 | 9.89 | 15.39 | 4.39 | 17.78 | 7.89 | Week 16 |  |  |
| Holden | 2024-04-21 | 111 | 10.11 | 15.67 | 4.61 | 15.56 | 5.44 | Week 16 |  |  |
| Holden | 2024-04-22 | 112 | 10.33 | 15.89 | 4.83 | 10 | -0.33 | Week 16 | 0.698 | 0.511 |
| Holden | 2024-04-23 | 113 | 10.56 | 16.11 | 5 | 5.56 | -5 | Week 17 |  |  |
| Holden | 2024-04-24 | 114 | 10.78 | 16.39 | 5.22 | 5.28 | -5.5 | Week 17 |  |  |
| Holden | 2024-04-25 | 115 | 11 | 16.61 | 5.44 | 5.56 | -5.44 | Week 17 |  |  |
| Holden | 2024-04-26 | 116 | 11.22 | 16.83 | 5.67 | 6.94 | -4.28 | Week 17 |  |  |
| Holden | 2024-04-27 | 117 | 11.44 | 17.06 | 5.83 | 7.78 | -3.67 | Week 17 |  |  |
| Holden | 2024-04-28 | 118 | 11.67 | 17.22 | 6.06 | 11.39 | -0.28 | Week 17 |  |  |
| Holden | 2024-04-29 | 119 | 11.89 | 17.44 | 6.28 | 14.17 | 2.28 | **Week 17** | **-2.772** | **0.032** |
| Holden | 2024-04-30 | 120 | 12.06 | 17.67 | 6.5 | 9.44 | -2.61 | Week 18 |  |  |
| Holden | 2024-05-01 | 121 | 12.28 | 17.89 | 6.67 | 5.83 | -6.44 | Week 18 |  |  |
| Holden | 2024-05-02 | 122 | 12.44 | 18.06 | 6.89 | 5 | -7.44 | Week 18 |  |  |
| Holden | 2024-05-03 | 123 | 12.67 | 18.28 | 7.06 | 7.5 | -5.17 | Week 18 |  |  |
| Holden | 2024-05-04 | 124 | 12.89 | 18.44 | 7.28 | 11.39 | -1.5 | Week 18 |  |  |
| Holden | 2024-05-05 | 125 | 13.06 | 18.61 | 7.5 | 11.94 | -1.11 | Week 18 |  |  |
| Holden | 2024-05-06 | 126 | 13.22 | 18.83 | 7.67 | 13.89 | 0.67 | **Week 18** | **-2.954** | **0.025** |
| Holden | 2024-05-07 | 127 | 13.44 | 19 | 7.89 | 19.72 | 6.28 | Week 19 |  |  |
| Holden | 2024-05-08 | 128 | 13.61 | 19.17 | 8.06 | 14.17 | 0.56 | Week 19 |  |  |
| Holden | 2024-05-09 | 129 | 13.78 | 19.33 | 8.28 | 12.22 | -1.56 | Week 19 |  |  |
| Holden | 2024-05-10 | 130 | 13.94 | 19.5 | 8.44 | 12.22 | -1.72 | Week 19 |  |  |
| Holden | 2024-05-11 | 131 | 14.17 | 19.67 | 8.61 | 16.39 | 2.22 | Week 19 |  |  |
| Holden | 2024-05-12 | 132 | 14.33 | 19.83 | 8.83 | 19.17 | 4.83 | Week 19 |  |  |
| Holden | 2024-05-13 | 133 | 14.5 | 20 | 9 | 19.17 | 4.67 | Week 19 | 1.799 | 0.122 |
| Holden | 2024-05-14 | 134 | 14.67 | 20.11 | 9.22 | 14.72 | 0.06 | Week 20 |  |  |
| Holden | 2024-05-15 | 135 | 14.83 | 20.28 | 9.39 | 13.61 | -1.22 | Week 20 |  |  |
| Holden | 2024-05-16 | 136 | 15 | 20.44 | 9.56 | 15 | 0 | Week 20 |  |  |
| Holden | 2024-05-17 | 137 | 15.17 | 20.61 | 9.72 | 9.72 | -5.44 | Week 20 |  |  |
| Holden | 2024-05-18 | 138 | 15.33 | 20.72 | 9.89 | 9.44 | -5.89 | Week 20 |  |  |
| Holden | 2024-05-19 | 139 | 15.5 | 20.89 | 10.11 | 18.06 | 2.56 | Week 20 |  |  |
| Holden | 2024-05-20 | 140 | 15.67 | 21.06 | 10.28 | 14.17 | -1.5 | Week 20 | -1.417 | 0.206 |
| Holden | 2024-05-21 | 141 | 15.83 | 21.17 | 10.44 | 16.11 | 0.28 | Week 21 |  |  |
| Holden | 2024-05-22 | 142 | 16 | 21.33 | 10.61 | 16.67 | 0.67 | Week 21 |  |  |
| Holden | 2024-05-23 | 143 | 16.11 | 21.5 | 10.78 | 18.89 | 2.78 | Week 21 |  |  |
| Holden | 2024-05-24 | 144 | 16.28 | 21.67 | 10.94 | 18.33 | 2.06 | Week 21 |  |  |
| Holden | 2024-05-25 | 145 | 16.44 | 21.78 | 11.11 | 11.67 | -4.78 | Week 21 |  |  |
| Holden | 2024-05-26 | 146 | 16.61 | 21.94 | 11.28 | 13.33 | -3.28 | Week 21 |  |  |
| Holden | 2024-05-27 | 147 | 16.78 | 22.11 | 11.44 | 15.83 | -0.94 | Week 21 | -0.443 | 0.674 |
| Holden | 2024-05-28 | 148 | 16.94 | 22.28 | 11.61 | 17.22 | 0.28 | Week 22 |  |  |
| Holden | 2024-05-29 | 149 | 17.11 | 22.39 | 11.78 | 19.44 | 2.33 | Week 22 |  |  |
| Holden | 2024-05-30 | 150 | 17.28 | 22.56 | 11.94 | 22.78 | 5.5 | Week 22 |  |  |
| Holden | 2024-05-31 | 151 | 17.44 | 22.72 | 12.11 | 23.06 | 5.61 | Week 22 |  |  |
| Holden | 2024-06-01 | 152 | 17.61 | 22.89 | 12.28 | 21.67 | 4.06 | Week 22 |  |  |
| Holden | 2024-06-02 | 153 | 17.78 | 23.06 | 12.44 | 21.39 | 3.61 | Week 22 |  |  |
| Holden | 2024-06-03 | 154 | 17.89 | 23.22 | 12.61 | 21.67 | 3.78 | **Week 22** | **5.142** | **0.002** |
| Holden | 2024-06-04 | 155 | 18.06 | 23.39 | 12.78 | 17.22 | -0.83 | Week 23 |  |  |
| Holden | 2024-06-05 | 156 | 18.22 | 23.56 | 12.94 | 16.39 | -1.83 | Week 23 |  |  |
| Holden | 2024-06-06 | 157 | 18.39 | 23.72 | 13.11 | 17.89 | -0.5 | Week 23 |  |  |
| Holden | 2024-06-07 | 158 | 18.56 | 23.89 | 13.22 | 16.11 | -2.44 | Week 23 |  |  |
| Holden | 2024-06-08 | 159 | 18.72 | 24.06 | 13.39 | 15.94 | -2.78 | Week 23 |  |  |
| Holden | 2024-06-09 | 160 | 18.89 | 24.22 | 13.56 | 18.22 | -0.67 | Week 23 |  |  |
| Holden | 2024-06-10 | 161 | 19.06 | 24.39 | 13.72 | 20.56 | 1.5 | Week 23 | -1.976 | 0.096 |
| Holden | 2024-06-11 | 162 | 19.22 | 24.56 | 13.89 | 19.61 | 0.39 | Week 24 |  |  |
| Holden | 2024-06-12 | 163 | 19.39 | 24.72 | 14 | 16.39 | -3 | Week 24 |  |  |
| Holden | 2024-06-13 | 164 | 19.56 | 24.89 | 14.17 | 14.39 | -5.17 | Week 24 |  |  |
| Holden | 2024-06-14 | 165 | 19.67 | 25.06 | 14.33 | 15.89 | -3.78 | Week 24 |  |  |
| Holden | 2024-06-15 | 166 | 19.83 | 25.22 | 14.44 | 18.06 | -1.78 | Week 24 |  |  |
| Holden | 2024-06-16 | 167 | 20 | 25.39 | 14.61 | 17.06 | -2.94 | Week 24 |  |  |
| Holden | 2024-06-17 | 168 | 20.17 | 25.56 | 14.72 | 19.11 | -1.06 | **Week 24** | **-3.576** | **0.012** |
| Holden | 2024-06-18 | 169 | 20.28 | 25.72 | 14.89 | 19.89 | -0.39 | Week 25 |  |  |
| Holden | 2024-06-19 | 170 | 20.44 | 25.89 | 15 | 21.61 | 1.17 | Week 25 |  |  |
| Holden | 2024-06-20 | 171 | 20.56 | 26 | 15.17 | 24.33 | 3.78 | Week 25 |  |  |
| Holden | 2024-06-21 | 172 | 20.72 | 26.17 | 15.28 | 24.22 | 3.5 | Week 25 |  |  |
| Holden | 2024-06-22 | 173 | 20.83 | 26.28 | 15.39 | 20.78 | -0.06 | Week 25 |  |  |
| Holden | 2024-06-23 | 174 | 21 | 26.44 | 15.56 | 19.78 | -1.22 | Week 25 |  |  |
| Holden | 2024-06-24 | 175 | 21.11 | 26.56 | 15.67 | 21.5 | 0.39 | Week 25 | 1.404 | 0.21 |
| Holden | 2024-06-25 | 176 | 21.22 | 26.67 | 15.78 | 24.17 | 2.94 | Week 26 |  |  |
| Holden | 2024-06-26 | 177 | 21.33 | 26.78 | 15.89 | 21.67 | 0.33 | Week 26 |  |  |
| Holden | 2024-06-27 | 178 | 21.44 | 26.89 | 16 | 19.17 | -2.28 | Week 26 |  |  |
| Holden | 2024-06-28 | 179 | 21.56 | 27 | 16.06 | 19.44 | -2.11 | Week 26 |  |  |
| Holden | 2024-06-29 | 180 | 21.67 | 27.11 | 16.17 | 22 | 0.33 | Week 26 |  |  |
| Holden | 2024-06-30 | 181 | 21.72 | 27.22 | 16.28 | 24.5 | 2.78 | Week 26 |  |  |
| Holden | 2024-07-01 | 182 | 21.83 | 27.28 | 16.33 | 24.61 | 2.78 | Week 26 | 0.798 | 0.455 |
| Holden | 2024-07-02 | 183 | 21.89 | 27.39 | 16.44 | 23.28 | 1.39 | Week 27 |  |  |
| Holden | 2024-07-03 | 184 | 22 | 27.44 | 16.5 | 22.78 | 0.78 | Week 27 |  |  |
| Holden | 2024-07-04 | 185 | 22.06 | 27.5 | 16.61 | 24.06 | 2 | Week 27 |  |  |
| Holden | 2024-07-05 | 186 | 22.11 | 27.56 | 16.67 | 26.28 | 4.17 | Week 27 |  |  |
| Holden | 2024-07-06 | 187 | 22.17 | 27.61 | 16.72 | 25.06 | 2.89 | Week 27 |  |  |
| Holden | 2024-07-07 | 188 | 22.22 | 27.67 | 16.78 | 22.33 | 0.11 | Week 27 |  |  |
| Holden | 2024-07-08 | 189 | 22.28 | 27.67 | 16.83 | 21.61 | -0.67 | Week 27 | 2.435 | 0.051 |
| Holden | 2024-07-09 | 190 | 22.28 | 27.72 | 16.89 | 21.44 | -0.83 | Week 28 |  |  |
| Holden | 2024-07-10 | 191 | 22.33 | 27.72 | 16.89 | 22.22 | -0.11 | Week 28 |  |  |
| Holden | 2024-07-11 | 192 | 22.33 | 27.72 | 16.94 | 24.11 | 1.78 | Week 28 |  |  |
| Holden | 2024-07-12 | 193 | 22.39 | 27.78 | 17 | 22.72 | 0.33 | Week 28 |  |  |
| Holden | 2024-07-13 | 194 | 22.39 | 27.78 | 17 | 21.83 | -0.56 | Week 28 |  |  |
| Holden | 2024-07-14 | 195 | 22.39 | 27.78 | 17.06 | 23.11 | 0.72 | Week 28 |  |  |
| Holden | 2024-07-15 | 196 | 22.39 | 27.72 | 17.06 | 22.5 | 0.11 | Week 28 | 0.629 | 0.553 |
| Holden | 2024-07-16 | 197 | 22.39 | 27.72 | 17.06 | 22.33 | -0.06 | Week 29 |  |  |
| Holden | 2024-07-17 | 198 | 22.39 | 27.72 | 17.06 | 23.17 | 0.78 | Week 29 |  |  |
| Holden | 2024-07-18 | 199 | 22.39 | 27.67 | 17.06 | 22.22 | -0.17 | Week 29 |  |  |
| Holden | 2024-07-19 | 200 | 22.39 | 27.67 | 17.06 | 22.06 | -0.33 | Week 29 |  |  |
| Holden | 2024-07-20 | 201 | 22.33 | 27.61 | 17.06 | 22.5 | 0.17 | Week 29 |  |  |
| Holden | 2024-07-21 | 202 | 22.33 | 27.61 | 17.06 | 21.17 | -1.17 | Week 29 |  |  |
| Holden | 2024-07-22 | 203 | 22.33 | 27.56 | 17.06 | 21.11 | -1.22 | Week 29 | -1.059 | 0.33 |
| Holden | 2024-07-23 | 204 | 22.28 | 27.56 | 17.06 | 22.56 | 0.28 | Week 30 |  |  |
| Holden | 2024-07-24 | 205 | 22.28 | 27.5 | 17 | 22.89 | 0.61 | Week 30 |  |  |
| Holden | 2024-07-25 | 206 | 22.22 | 27.44 | 17 | 24.17 | 1.94 | Week 30 |  |  |
| Holden | 2024-07-26 | 207 | 22.22 | 27.44 | 16.94 | 23.89 | 1.67 | Week 30 |  |  |
| Holden | 2024-07-27 | 208 | 22.17 | 27.39 | 16.94 | 24 | 1.83 | Week 30 |  |  |
| Holden | 2024-07-28 | 209 | 22.11 | 27.33 | 16.94 | 25.67 | 3.56 | Week 30 |  |  |
| Holden | 2024-07-29 | 210 | 22.11 | 27.28 | 16.89 | 23.28 | 1.17 | **Week 30** | **3.893** | **0.008** |
| Holden | 2024-07-30 | 211 | 22.06 | 27.28 | 16.83 | 21.67 | -0.39 | Week 31 |  |  |
| Holden | 2024-07-31 | 212 | 22 | 27.22 | 16.83 | 20.83 | -1.17 | Week 31 |  |  |
| Holden | 2024-08-01 | 213 | 22 | 27.17 | 16.78 | 20.67 | -1.33 | Week 31 |  |  |
| Holden | 2024-08-02 | 214 | 21.94 | 27.11 | 16.72 | 21.83 | -0.11 | Week 31 |  |  |
| Holden | 2024-08-03 | 215 | 21.89 | 27.11 | 16.72 | 22.89 | 1 | Week 31 |  |  |
| Holden | 2024-08-04 | 216 | 21.89 | 27.06 | 16.67 | 23.33 | 1.44 | Week 31 |  |  |
| Holden | 2024-08-05 | 217 | 21.83 | 27 | 16.61 | 22.33 | 0.5 | Week 31 | -0.02 | 0.985 |
| Holden | 2024-08-06 | 218 | 21.78 | 27 | 16.61 | 24.5 | 2.72 | Week 32 |  |  |
| Holden | 2024-08-07 | 219 | 21.72 | 26.94 | 16.56 | 20.78 | -0.94 | Week 32 |  |  |
| Holden | 2024-08-08 | 220 | 21.72 | 26.94 | 16.5 | 21.5 | -0.22 | Week 32 |  |  |
| Holden | 2024-08-09 | 221 | 21.67 | 26.89 | 16.44 | 23.06 | 1.39 | Week 32 |  |  |
| Holden | 2024-08-10 | 222 | 21.61 | 26.83 | 16.39 | 22.67 | 1.06 | Week 32 |  |  |
| Holden | 2024-08-11 | 223 | 21.61 | 26.83 | 16.33 | 21.78 | 0.17 | Week 32 |  |  |
| Holden | 2024-08-12 | 224 | 21.56 | 26.78 | 16.28 | 23.56 | 2 | Week 32 | 1.806 | 0.121 |
| Holden | 2024-08-13 | 225 | 21.5 | 26.78 | 16.22 | 22.72 | 1.22 | Week 33 |  |  |
| Holden | 2024-08-14 | 226 | 21.44 | 26.72 | 16.17 | 22.44 | 1 | Week 33 |  |  |
| Holden | 2024-08-15 | 227 | 21.44 | 26.72 | 16.11 | 20.67 | -0.78 | Week 33 |  |  |
| Holden | 2024-08-16 | 228 | 21.39 | 26.67 | 16.06 | 21.56 | 0.17 | Week 33 |  |  |
| Holden | 2024-08-17 | 229 | 21.33 | 26.61 | 16 | 20.78 | -0.56 | Week 33 |  |  |
| Holden | 2024-08-18 | 230 | 21.28 | 26.61 | 15.94 | 19.06 | -2.22 | Week 33 |  |  |
| Holden | 2024-08-19 | 231 | 21.22 | 26.56 | 15.89 | 19.33 | -1.89 | Week 33 | -0.869 | 0.419 |
| Holden | 2024-08-20 | 232 | 21.17 | 26.5 | 15.83 | 24.06 | 2.89 | Week 34 |  |  |
| Holden | 2024-08-21 | 233 | 21.11 | 26.5 | 15.78 | 23.44 | 2.33 | Week 34 |  |  |
| Holden | 2024-08-22 | 234 | 21.06 | 26.44 | 15.67 | 20.61 | -0.44 | Week 34 |  |  |
| Holden | 2024-08-23 | 235 | 21 | 26.39 | 15.61 | 20.61 | -0.39 | Week 34 |  |  |
| Holden | 2024-08-24 | 236 | 20.94 | 26.33 | 15.56 | 23.89 | 2.94 | Week 34 |  |  |
| Holden | 2024-08-25 | 237 | 20.89 | 26.28 | 15.44 | 21.83 | 0.94 | Week 34 |  |  |
| Holden | 2024-08-26 | 238 | 20.78 | 26.22 | 15.39 | 20.5 | -0.28 | Week 34 | 1.936 | 0.101 |
| Holden | 2024-08-27 | 239 | 20.72 | 26.17 | 15.28 | 20.33 | -0.39 | Week 35 |  |  |
| Holden | 2024-08-28 | 240 | 20.67 | 26.06 | 15.22 | 17.61 | -3.06 | Week 35 |  |  |
| Holden | 2024-08-29 | 241 | 20.56 | 26 | 15.11 | 18.83 | -1.72 | Week 35 |  |  |
| Holden | 2024-08-30 | 242 | 20.44 | 25.94 | 15 | 17.5 | -2.94 | Week 35 |  |  |
| Holden | 2024-08-31 | 243 | 20.39 | 25.83 | 14.89 | 17.78 | -2.61 | Week 35 |  |  |
| Holden | 2024-09-01 | 244 | 20.28 | 25.72 | 14.83 | 19.39 | -0.89 | Week 35 |  |  |
| Holden | 2024-09-02 | 245 | 20.17 | 25.67 | 14.72 | 22.06 | 1.89 | Week 35 | -2.076 | 0.083 |
| Holden | 2024-09-03 | 246 | 20.06 | 25.56 | 14.56 | 25.17 | 5.11 | Week 36 |  |  |
| Holden | 2024-09-04 | 247 | 19.94 | 25.44 | 14.44 | 26.28 | 6.33 | Week 36 |  |  |
| Holden | 2024-09-05 | 248 | 19.83 | 25.28 | 14.33 | 26.83 | 7 | Week 36 |  |  |
| Holden | 2024-09-06 | 249 | 19.67 | 25.17 | 14.22 | 25.78 | 6.11 | Week 36 |  |  |
| Holden | 2024-09-07 | 250 | 19.56 | 25.06 | 14.06 | 23.11 | 3.56 | Week 36 |  |  |
| Holden | 2024-09-08 | 251 | 19.44 | 24.89 | 13.94 | 19.94 | 0.5 | Week 36 |  |  |
| Holden | 2024-09-09 | 252 | 19.28 | 24.72 | 13.78 | 18.94 | -0.33 | **Week 36** | **3.653** | **0.011** |
| Holden | 2024-09-10 | 253 | 19.11 | 24.56 | 13.67 | 18.56 | -0.56 | Week 37 |  |  |
| Holden | 2024-09-11 | 254 | 18.94 | 24.44 | 13.5 | 19.06 | 0.11 | Week 37 |  |  |
| Holden | 2024-09-12 | 255 | 18.78 | 24.22 | 13.33 | 19 | 0.22 | Week 37 |  |  |
| Holden | 2024-09-13 | 256 | 18.61 | 24.06 | 13.22 | 16.56 | -2.06 | Week 37 |  |  |
| Holden | 2024-09-14 | 257 | 18.44 | 23.89 | 13.06 | 15.56 | -2.89 | Week 37 |  |  |
| Holden | 2024-09-15 | 258 | 18.28 | 23.72 | 12.89 | 15.33 | -2.94 | Week 37 |  |  |
| Holden | 2024-09-16 | 259 | 18.11 | 23.5 | 12.72 | 15.83 | -2.28 | **Week 37** | **-2.851** | **0.029** |
| Holden | 2024-09-17 | 260 | 17.94 | 23.28 | 12.56 | 18.17 | 0.22 | Week 38 |  |  |
| Holden | 2024-09-18 | 261 | 17.72 | 23.11 | 12.33 | 17.78 | 0.06 | Week 38 |  |  |
| Holden | 2024-09-19 | 262 | 17.56 | 22.89 | 12.17 | 16.72 | -0.83 | Week 38 |  |  |
| Holden | 2024-09-20 | 263 | 17.33 | 22.67 | 12 | 19.44 | 2.11 | Week 38 |  |  |
| Holden | 2024-09-21 | 264 | 17.11 | 22.44 | 11.83 | 20.28 | 3.17 | Week 38 |  |  |
| Holden | 2024-09-22 | 265 | 16.94 | 22.22 | 11.61 | 19.89 | 2.94 | Week 38 |  |  |
| Holden | 2024-09-23 | 266 | 16.72 | 22 | 11.44 | 18.06 | 1.33 | Week 38 | 2.218 | 0.068 |
| Holden | 2024-09-24 | 267 | 16.5 | 21.78 | 11.28 | 18.33 | 1.83 | Week 39 |  |  |
| Holden | 2024-09-25 | 268 | 16.28 | 21.5 | 11.06 | 18.5 | 2.22 | Week 39 |  |  |
| Holden | 2024-09-26 | 269 | 16.06 | 21.28 | 10.89 | 18.56 | 2.5 | Week 39 |  |  |
| Holden | 2024-09-27 | 270 | 15.83 | 21.06 | 10.67 | 17.33 | 1.5 | Week 39 |  |  |
| Holden | 2024-09-28 | 271 | 15.67 | 20.78 | 10.5 | 16.5 | 0.83 | Week 39 |  |  |
| Holden | 2024-09-29 | 272 | 15.44 | 20.56 | 10.28 | 18.17 | 2.72 | Week 39 |  |  |
| Holden | 2024-09-30 | 273 | 15.22 | 20.33 | 10.11 | 17.67 | 2.44 | **Week 39** | **7.983** | **<0.001** |
| Holden | 2024-10-01 | 274 | 15 | 20.06 | 9.89 | 16.33 | 1.33 | Week 40 |  |  |
| Holden | 2024-10-02 | 275 | 14.78 | 19.83 | 9.72 | 20.17 | 5.39 | Week 40 |  |  |
| Holden | 2024-10-03 | 276 | 14.56 | 19.56 | 9.5 | 21.94 | 7.39 | Week 40 |  |  |
| Holden | 2024-10-04 | 277 | 14.33 | 19.33 | 9.33 | 22.5 | 8.17 | Week 40 |  |  |
| Holden | 2024-10-05 | 278 | 14.11 | 19.06 | 9.11 | 20.83 | 6.72 | Week 40 |  |  |
| Holden | 2024-10-06 | 279 | 13.89 | 18.83 | 8.94 | 17.33 | 3.44 | Week 40 |  |  |
| Holden | 2024-10-07 | 280 | 13.67 | 18.61 | 8.72 | 9.89 | -3.78 | **Week 40** | **2.577** | **0.042** |
| Holden | 2024-10-08 | 281 | 13.44 | 18.33 | 8.56 | 9.56 | -3.89 | Week 41 |  |  |
| Holden | 2024-10-09 | 282 | 13.22 | 18.11 | 8.33 | 9.33 | -3.89 | Week 41 |  |  |
| Holden | 2024-10-10 | 283 | 13 | 17.89 | 8.17 | 10.44 | -2.56 | Week 41 |  |  |
| Holden | 2024-10-11 | 284 | 12.78 | 17.61 | 7.94 | 12.61 | -0.17 | Week 41 |  |  |
| Holden | 2024-10-12 | 285 | 12.56 | 17.39 | 7.78 | 12.78 | 0.22 | Week 41 |  |  |
| Holden | 2024-10-13 | 286 | 12.39 | 17.17 | 7.56 | 13.61 | 1.22 | Week 41 |  |  |
| Holden | 2024-10-14 | 287 | 12.17 | 16.89 | 7.39 | 10.72 | -1.44 | Week 41 | -1.957 | 0.098 |
| Holden | 2024-10-15 | 288 | 11.94 | 16.67 | 7.22 | 10.83 | -1.11 | Week 42 |  |  |
| Holden | 2024-10-16 | 289 | 11.72 | 16.44 | 7 | 10.89 | -0.83 | Week 42 |  |  |
| Holden | 2024-10-17 | 290 | 11.5 | 16.22 | 6.83 | 9.94 | -1.56 | Week 42 |  |  |
| Holden | 2024-10-18 | 291 | 11.33 | 16 | 6.67 | 11.5 | 0.17 | Week 42 |  |  |
| Holden | 2024-10-19 | 292 | 11.11 | 15.78 | 6.44 | 13.5 | 2.39 | Week 42 |  |  |
| Holden | 2024-10-20 | 293 | 10.89 | 15.56 | 6.28 | 12.94 | 2.06 | Week 42 |  |  |
| Holden | 2024-10-21 | 294 | 10.72 | 15.33 | 6.11 | 10.39 | -0.33 | Week 42 | 0.19 | 0.855 |
| Holden | 2024-10-22 | 295 | 10.5 | 15.11 | 5.94 | 8.44 | -2.06 | Week 43 |  |  |
| Holden | 2024-10-23 | 296 | 10.33 | 14.89 | 5.72 | 8.11 | -2.22 | Week 43 |  |  |
| Holden | 2024-10-24 | 297 | 10.11 | 14.67 | 5.56 | 17.78 | 7.67 | Week 43 |  |  |
| Holden | 2024-10-25 | 298 | 9.89 | 14.44 | 5.39 | 19.94 | 10.06 | Week 43 |  |  |
| Holden | 2024-10-26 | 299 | 9.72 | 14.22 | 5.22 | 20.11 | 10.39 | Week 43 |  |  |
| Holden | 2024-10-27 | 300 | 9.5 | 14 | 5.06 | 20.5 | 11 | Week 43 |  |  |
| Holden | 2024-10-28 | 301 | 9.33 | 13.78 | 4.83 | 14.94 | 5.61 | **Week 43** | **2.676** | **0.037** |
| Holden | 2024-10-29 | 302 | 9.11 | 13.56 | 4.67 | 14.94 | 5.83 | Week 44 |  |  |
| Holden | 2024-10-30 | 303 | 8.89 | 13.33 | 4.5 | 7.89 | -1 | Week 44 |  |  |
| Holden | 2024-10-31 | 304 | 8.72 | 13.11 | 4.33 | 3.22 | -5.5 | Week 44 | -0.067 | 0.952 |
| CBG | 2024-01-01 | 1 | -3.33 | 0.94 | -7.56 | 5.00 | 8.33 | Week 1 |  |  |
| CBG | 2024-01-02 | 2 | -3.44 | 0.83 | -7.67 | 3.33 | 6.78 | Week 1 |  |  |
| CBG | 2024-01-03 | 3 | -3.50 | 0.78 | -7.78 | 6.94 | 10.44 | Week 1 |  |  |
| CBG | 2024-01-04 | 4 | -3.61 | 0.67 | -7.89 | 4.17 | 7.78 | Week 1 |  |  |
| CBG | 2024-01-05 | 5 | -3.72 | 0.61 | -8.00 | 0.28 | 4.00 | Week 1 |  |  |
| CBG | 2024-01-06 | 6 | -3.83 | 0.50 | -8.11 | -1.67 | 2.17 | Week 1 |  |  |
| CBG | 2024-01-07 | 7 | -3.89 | 0.44 | -8.22 | -1.11 | 2.78 | **Week 1** | **5.138** | **0.002** |
| CBG | 2024-01-08 | 8 | -4.00 | 0.33 | -8.33 | -0.83 | 3.17 | Week 2 |  |  |
| CBG | 2024-01-09 | 9 | -4.06 | 0.28 | -8.39 | 1.39 | 5.44 | Week 2 |  |  |
| CBG | 2024-01-10 | 10 | -4.17 | 0.22 | -8.50 | 5.83 | 10.00 | Week 2 |  |  |
| CBG | 2024-01-11 | 11 | -4.22 | 0.17 | -8.61 | 7.50 | 11.72 | Week 2 |  |  |
| CBG | 2024-01-12 | 12 | -4.28 | 0.11 | -8.67 | 4.44 | 8.72 | Week 2 |  |  |
| CBG | 2024-01-13 | 13 | -4.33 | 0.06 | -8.72 | -0.56 | 3.78 | Week 2 |  |  |
| CBG | 2024-01-14 | 14 | -4.39 | 0.00 | -8.83 | -2.50 | 1.89 | **Week 2** | **4.484** | **0.004** |
| CBG | 2024-01-15 | 15 | -4.44 | -0.06 | -8.89 | 1.39 | 5.83 | Week 3 |  |  |
| CBG | 2024-01-16 | 16 | -4.50 | -0.06 | -8.94 | 7.78 | 12.28 | Week 3 |  |  |
| CBG | 2024-01-17 | 17 | -4.56 | -0.11 | -9.00 | 6.39 | 10.94 | Week 3 |  |  |
| CBG | 2024-01-18 | 18 | -4.61 | -0.11 | -9.06 | 3.06 | 7.67 | Week 3 |  |  |
| CBG | 2024-01-19 | 19 | -4.61 | -0.11 | -9.11 | 3.33 | 7.94 | Week 3 |  |  |
| CBG | 2024-01-20 | 20 | -4.67 | -0.17 | -9.17 | 0.28 | 4.94 | Week 3 |  |  |
| CBG | 2024-01-21 | 21 | -4.67 | -0.17 | -9.17 | -1.11 | 3.56 | **Week 3** | **6.369** | **0.001** |
| CBG | 2024-01-22 | 22 | -4.67 | -0.17 | -9.22 | -0.28 | 4.39 | Week 4 |  |  |
| CBG | 2024-01-23 | 23 | -4.67 | -0.11 | -9.22 | -2.78 | 1.89 | Week 4 |  |  |
| CBG | 2024-01-24 | 24 | -4.67 | -0.11 | -9.22 | 0.28 | 4.94 | Week 4 |  |  |
| CBG | 2024-01-25 | 25 | -4.67 | -0.11 | -9.22 | 0.56 | 5.22 | Week 4 |  |  |
| CBG | 2024-01-26 | 26 | -4.61 | -0.06 | -9.22 | -4.72 | -0.11 | Week 4 |  |  |
| CBG | 2024-01-27 | 27 | -4.61 | 0.00 | -9.17 | -3.61 | 1.00 | Week 4 |  |  |
| CBG | 2024-01-28 | 28 | -4.56 | 0.06 | -9.17 | -3.61 | 0.94 | **Week 4** | **3.157** | **0.020** |
| CBG | 2024-01-29 | 29 | -4.50 | 0.11 | -9.11 | -4.17 | 0.33 | Week 5 |  |  |
| CBG | 2024-01-30 | 30 | -4.44 | 0.17 | -9.06 | -10.83 | -6.39 | Week 5 |  |  |
| CBG | 2024-01-31 | 31 | -4.39 | 0.22 | -9.06 | -13.06 | -8.67 | Week 5 |  |  |
| CBG | 2024-02-01 | 32 | -4.33 | 0.33 | -8.94 | -8.33 | -4.00 | Week 5 |  |  |
| CBG | 2024-02-02 | 33 | -4.22 | 0.39 | -8.89 | -5.00 | -0.78 | Week 5 |  |  |
| CBG | 2024-02-03 | 34 | -4.17 | 0.50 | -8.83 | -14.17 | -10.00 | Week 5 |  |  |
| CBG | 2024-02-04 | 35 | -4.06 | 0.61 | -8.72 | -2.50 | 1.56 | Week 5 | -2.328 | 0.059 |
| CBG | 2024-02-05 | 36 | -3.94 | 0.72 | -8.61 | 2.22 | 6.17 | Week 6 |  |  |
| CBG | 2024-02-06 | 37 | -3.83 | 0.83 | -8.50 | 1.67 | 5.50 | Week 6 |  |  |
| CBG | 2024-02-07 | 38 | -3.72 | 0.94 | -8.39 | 4.44 | 8.17 | Week 6 |  |  |
| CBG | 2024-02-08 | 39 | -3.61 | 1.11 | -8.28 | 1.11 | 4.72 | Week 6 |  |  |
| CBG | 2024-02-09 | 40 | -3.44 | 1.22 | -8.17 | 5.28 | 8.72 | Week 6 |  |  |
| CBG | 2024-02-10 | 41 | -3.33 | 1.39 | -8.00 | 0.56 | 3.89 | Week 6 |  |  |
| CBG | 2024-02-11 | 42 | -3.17 | 1.50 | -7.89 | 1.67 | 4.83 | **Week 6** | **8.731** | **<0.001** |
| CBG | 2024-02-12 | 43 | -3.00 | 1.67 | -7.72 | 4.44 | 7.44 | Week 7 |  |  |
| CBG | 2024-02-13 | 44 | -2.89 | 1.83 | -7.56 | 6.39 | 9.28 | Week 7 |  |  |
| CBG | 2024-02-14 | 45 | -2.72 | 2.00 | -7.39 | 7.22 | 9.94 | Week 7 |  |  |
| CBG | 2024-02-15 | 46 | -2.56 | 2.17 | -7.22 | 6.94 | 9.50 | Week 7 |  |  |
| CBG | 2024-02-16 | 47 | -2.39 | 2.33 | -7.06 | -0.83 | 1.56 | Week 7 |  |  |
| CBG | 2024-02-17 | 48 | -2.17 | 2.50 | -6.89 | -5.00 | -2.83 | Week 7 |  |  |
| CBG | 2024-02-18 | 49 | -2.00 | 2.72 | -6.72 | 1.67 | 3.67 | **Week 7** | **2.994** | **0.024** |
| CBG | 2024-02-19 | 50 | -1.83 | 2.89 | -6.56 | 7.78 | 9.61 | Week 8 |  |  |
| CBG | 2024-02-20 | 51 | -1.61 | 3.06 | -6.33 | 3.33 | 4.94 | Week 8 |  |  |
| CBG | 2024-02-21 | 52 | -1.44 | 3.28 | -6.17 | 0.56 | 2.00 | Week 8 |  |  |
| CBG | 2024-02-22 | 53 | -1.28 | 3.44 | -5.94 | 1.67 | 2.94 | Week 8 |  |  |
| CBG | 2024-02-23 | 54 | -1.06 | 3.67 | -5.78 | 0.56 | 1.61 | Week 8 |  |  |
| CBG | 2024-02-24 | 55 | -0.89 | 3.83 | -5.61 | -5.00 | -4.11 | Week 8 |  |  |
| CBG | 2024-02-25 | 56 | -0.67 | 4.06 | -5.39 | 0.28 | 0.94 | Week 8 | 1.630 | 0.154 |
| CBG | 2024-02-26 | 57 | -0.50 | 4.22 | -5.22 | 3.89 | 4.39 | Week 9 |  |  |
| CBG | 2024-02-27 | 58 | -0.28 | 4.44 | -5.00 | 9.44 | 9.72 | Week 9 |  |  |
| CBG | 2024-02-28 | 59 | -0.11 | 4.61 | -4.83 | 6.67 | 6.78 | Week 9 |  |  |
| CBG | 2024-03-01 | 60 | 0.11 | 4.83 | -4.61 | 8.33 | 8.22 | Week 9 |  |  |
| CBG | 2024-03-02 | 61 | 0.28 | 5.00 | -4.44 | 2.50 | 2.22 | Week 9 |  |  |
| CBG | 2024-03-03 | 62 | 0.44 | 5.22 | -4.28 | 1.94 | 1.50 | Week 9 |  |  |
| CBG | 2024-03-04 | 63 | 0.67 | 5.39 | -4.06 | 3.89 | 3.22 | **Week 9** | **4.339** | **0.005** |
| CBG | 2024-03-05 | 64 | 0.83 | 5.61 | -3.89 | 4.17 | 3.33 | Week 10 |  |  |
| CBG | 2024-03-06 | 65 | 1.06 | 5.78 | -3.72 | 8.33 | 7.28 | Week 10 |  |  |
| CBG | 2024-03-07 | 66 | 1.22 | 6.00 | -3.56 | 4.44 | 3.22 | Week 10 |  |  |
| CBG | 2024-03-08 | 67 | 1.39 | 6.17 | -3.39 | 5.00 | 3.61 | Week 10 |  |  |
| CBG | 2024-03-09 | 68 | 1.56 | 6.39 | -3.22 | 2.78 | 1.22 | Week 10 |  |  |
| CBG | 2024-03-10 | 69 | 1.78 | 6.56 | -3.06 | 1.67 | -0.11 | Week 10 |  |  |
| CBG | 2024-03-11 | 70 | 1.94 | 6.72 | -2.89 | 2.22 | 0.28 | **Week 10** | **2.813** | **0.031** |
| CBG | 2024-03-12 | 71 | 2.11 | 6.94 | -2.72 | 0.83 | -1.28 | Week 11 |  |  |
| CBG | 2024-03-13 | 72 | 2.28 | 7.11 | -2.56 | -1.39 | -3.67 | Week 11 |  |  |
| CBG | 2024-03-14 | 73 | 2.44 | 7.28 | -2.39 | -1.11 | -3.56 | Week 11 |  |  |
| CBG | 2024-03-15 | 74 | 2.61 | 7.50 | -2.22 | 3.06 | 0.44 | Week 11 |  |  |
| CBG | 2024-03-16 | 75 | 2.83 | 7.67 | -2.06 | 8.33 | 5.50 | Week 11 |  |  |
| CBG | 2024-03-17 | 76 | 3.00 | 7.83 | -1.89 | 3.06 | 0.06 | Week 11 |  |  |
| CBG | 2024-03-18 | 77 | 3.17 | 8.06 | -1.78 | -6.94 | -10.11 | Week 11 | -0.995 | 0.358 |
| CBG | 2024-03-19 | 78 | 3.33 | 8.22 | -1.61 | -1.11 | -4.44 | Week 12 |  |  |
| CBG | 2024-03-20 | 79 | 3.50 | 8.39 | -1.44 | 4.44 | 0.94 | Week 12 |  |  |
| CBG | 2024-03-21 | 80 | 3.67 | 8.61 | -1.28 | 8.06 | 4.39 | Week 12 |  |  |
| CBG | 2024-03-22 | 81 | 3.83 | 8.78 | -1.17 | 8.33 | 4.50 | Week 12 |  |  |
| CBG | 2024-03-23 | 82 | 4.00 | 8.94 | -1.00 | 6.11 | 2.11 | Week 12 |  |  |
| CBG | 2024-03-24 | 83 | 4.17 | 9.17 | -0.83 | 5.00 | 0.83 | Week 12 |  |  |
| CBG | 2024-03-25 | 84 | 4.33 | 9.33 | -0.67 | 4.44 | 0.11 | Week 12 | 1.053 | 0.333 |
| CBG | 2024-03-26 | 85 | 4.50 | 9.56 | -0.56 | 5.00 | 0.50 | Week 13 |  |  |
| CBG | 2024-03-27 | 86 | 4.67 | 9.72 | -0.39 | 4.17 | -0.50 | Week 13 |  |  |
| CBG | 2024-03-28 | 87 | 4.83 | 9.89 | -0.22 | 5.00 | 0.17 | Week 13 |  |  |
| CBG | 2024-03-29 | 88 | 5.00 | 10.11 | -0.06 | 2.50 | -2.50 | Week 13 |  |  |
| CBG | 2024-03-30 | 89 | 5.22 | 10.28 | 0.11 | 5.83 | 0.61 | Week 13 |  |  |
| CBG | 2024-03-31 | 90 | 5.39 | 10.50 | 0.28 | 15.56 | 10.17 | Week 13 |  |  |
| CBG | 2024-04-01 | 91 | 5.56 | 10.72 | 0.44 | 5.83 | 0.28 | Week 13 | 0.809 | 0.450 |
| CBG | 2024-04-02 | 92 | 5.72 | 10.89 | 0.61 | 6.67 | 0.94 | Week 14 |  |  |
| CBG | 2024-04-03 | 93 | 5.94 | 11.11 | 0.78 | 11.67 | 5.72 | Week 14 |  |  |
| CBG | 2024-04-04 | 94 | 6.11 | 11.28 | 0.94 | 14.72 | 8.61 | Week 14 |  |  |
| CBG | 2024-04-05 | 95 | 6.28 | 11.50 | 1.11 | 13.33 | 7.06 | Week 14 |  |  |
| CBG | 2024-04-06 | 96 | 6.50 | 11.72 | 1.28 | 6.39 | -0.11 | Week 14 |  |  |
| CBG | 2024-04-07 | 97 | 6.67 | 11.94 | 1.44 | 6.39 | -0.28 | Week 14 |  |  |
| CBG | 2024-04-08 | 98 | 6.89 | 12.11 | 1.61 | 10.56 | 3.67 | **Week 14** | **2.695** | **0.036** |
| CBG | 2024-04-09 | 99 | 7.06 | 12.33 | 1.83 | 13.33 | 6.28 | Week 15 |  |  |
| CBG | 2024-04-10 | 100 | 7.28 | 12.56 | 2.00 | 15.28 | 8.00 | Week 15 |  |  |
| CBG | 2024-04-11 | 101 | 7.50 | 12.78 | 2.22 | 18.89 | 11.39 | Week 15 |  |  |
| CBG | 2024-04-12 | 102 | 7.67 | 13.00 | 2.39 | 21.67 | 14.00 | Week 15 |  |  |
| CBG | 2024-04-13 | 103 | 7.89 | 13.22 | 2.56 | 21.11 | 13.22 | Week 15 |  |  |
| CBG | 2024-04-14 | 104 | 8.11 | 13.44 | 2.78 | 21.11 | 13.00 | Week 15 |  |  |
| CBG | 2024-04-15 | 105 | 8.33 | 13.67 | 3.00 | 23.06 | 14.72 | **Week 15** | **9.526** | **<0.001** |
| CBG | 2024-04-16 | 106 | 8.50 | 13.89 | 3.17 | 9.72 | 1.22 | Week 16 |  |  |
| CBG | 2024-04-17 | 107 | 8.72 | 14.11 | 3.39 | 2.22 | -6.50 | Week 16 |  |  |
| CBG | 2024-04-18 | 108 | 8.94 | 14.33 | 3.56 | 7.78 | -1.17 | Week 16 |  |  |
| CBG | 2024-04-19 | 109 | 9.17 | 14.56 | 3.78 | 14.44 | 5.28 | Week 16 |  |  |
| CBG | 2024-04-20 | 110 | 9.33 | 14.78 | 3.94 | 18.33 | 9.00 | Week 16 |  |  |
| CBG | 2024-04-21 | 111 | 9.56 | 15.00 | 4.17 | 12.22 | 2.67 | Week 16 |  |  |
| CBG | 2024-04-22 | 112 | 9.78 | 15.22 | 4.39 | 5.83 | -3.94 | Week 16 | 0.465 | 0.658 |
| CBG | 2024-04-23 | 113 | 10.00 | 15.39 | 4.56 | 3.89 | -6.11 | Week 17 |  |  |
| CBG | 2024-04-24 | 114 | 10.22 | 15.61 | 4.78 | 6.94 | -3.28 | Week 17 |  |  |
| CBG | 2024-04-25 | 115 | 10.39 | 15.83 | 4.94 | 5.00 | -5.39 | Week 17 |  |  |
| CBG | 2024-04-26 | 116 | 10.61 | 16.06 | 5.17 | 5.56 | -5.06 | Week 17 |  |  |
| CBG | 2024-04-27 | 117 | 10.83 | 16.28 | 5.33 | 11.39 | 0.56 | Week 17 |  |  |
| CBG | 2024-04-28 | 118 | 11.06 | 16.50 | 5.56 | 12.22 | 1.17 | Week 17 |  |  |
| CBG | 2024-04-29 | 119 | 11.22 | 16.72 | 5.72 | 10.28 | -0.94 | Week 17 | -2.415 | 0.052 |
| CBG | 2024-04-30 | 120 | 11.44 | 16.94 | 5.94 | 7.22 | -4.22 | Week 18 |  |  |
| CBG | 2024-05-01 | 121 | 11.61 | 17.17 | 6.11 | 7.50 | -4.11 | Week 18 |  |  |
| CBG | 2024-05-02 | 122 | 11.83 | 17.33 | 6.33 | 9.44 | -2.39 | Week 18 |  |  |
| CBG | 2024-05-03 | 123 | 12.06 | 17.56 | 6.50 | 9.44 | -2.61 | Week 18 |  |  |
| CBG | 2024-05-04 | 124 | 12.22 | 17.78 | 6.67 | 13.89 | 1.67 | Week 18 |  |  |
| CBG | 2024-05-05 | 125 | 12.39 | 18.00 | 6.83 | 19.72 | 7.33 | Week 18 |  |  |
| CBG | 2024-05-06 | 126 | 12.61 | 18.17 | 7.00 | 20.28 | 7.67 | Week 18 | 0.243 | 0.816 |
| CBG | 2024-05-07 | 127 | 12.78 | 18.39 | 7.22 | 22.50 | 9.72 | Week 19 |  |  |
| CBG | 2024-05-08 | 128 | 13.00 | 18.56 | 7.39 | 15.28 | 2.28 | Week 19 |  |  |
| CBG | 2024-05-09 | 129 | 13.17 | 18.78 | 7.56 | 15.28 | 2.11 | Week 19 |  |  |
| CBG | 2024-05-10 | 130 | 13.33 | 19.00 | 7.72 | 18.61 | 5.28 | Week 19 |  |  |
| CBG | 2024-05-11 | 131 | 13.50 | 19.17 | 7.89 | 21.11 | 7.61 | Week 19 |  |  |
| CBG | 2024-05-12 | 132 | 13.72 | 19.39 | 8.06 | 20.00 | 6.28 | Week 19 |  |  |
| CBG | 2024-05-13 | 133 | 13.89 | 19.56 | 8.22 | 16.94 | 3.06 | **Week 19** | **4.752** | **0.003** |
| CBG | 2024-05-14 | 134 | 14.06 | 19.78 | 8.39 | 15.56 | 1.50 | Week 20 |  |  |
| CBG | 2024-05-15 | 135 | 14.22 | 19.94 | 8.50 | 15.83 | 1.61 | Week 20 |  |  |
| CBG | 2024-05-16 | 136 | 14.39 | 20.11 | 8.67 | 18.89 | 4.50 | Week 20 |  |  |
| CBG | 2024-05-17 | 137 | 14.61 | 20.33 | 8.83 | 12.78 | -1.83 | Week 20 |  |  |
| CBG | 2024-05-18 | 138 | 14.78 | 20.50 | 9.00 | 16.11 | 1.33 | Week 20 |  |  |
| CBG | 2024-05-19 | 139 | 14.94 | 20.72 | 9.17 | 18.06 | 3.11 | Week 20 |  |  |
| CBG | 2024-05-20 | 140 | 15.11 | 20.89 | 9.33 | 15.83 | 0.72 | Week 20 | 2.098 | 0.081 |
| CBG | 2024-05-21 | 141 | 15.28 | 21.06 | 9.50 | 19.44 | 4.17 | Week 21 |  |  |
| CBG | 2024-05-22 | 142 | 15.44 | 21.28 | 9.67 | 20.83 | 5.39 | Week 21 |  |  |
| CBG | 2024-05-23 | 143 | 15.61 | 21.44 | 9.78 | 21.94 | 6.33 | Week 21 |  |  |
| CBG | 2024-05-24 | 144 | 15.83 | 21.67 | 9.94 | 15.28 | -0.56 | Week 21 |  |  |
| CBG | 2024-05-25 | 145 | 16.00 | 21.83 | 10.11 | 12.22 | -3.78 | Week 21 |  |  |
| CBG | 2024-05-26 | 146 | 16.17 | 22.06 | 10.28 | 15.56 | -0.61 | Week 21 |  |  |
| CBG | 2024-05-27 | 147 | 16.33 | 22.22 | 10.44 | 16.11 | -0.22 | Week 21 | 1.076 | 0.323 |
| CBG | 2024-05-28 | 148 | 16.50 | 22.39 | 10.61 | 19.72 | 3.22 | Week 22 |  |  |
| CBG | 2024-05-29 | 149 | 16.72 | 22.61 | 10.83 | 20.56 | 3.83 | Week 22 |  |  |
| CBG | 2024-05-30 | 150 | 16.89 | 22.78 | 11.00 | 24.17 | 7.28 | Week 22 |  |  |
| CBG | 2024-05-31 | 151 | 17.06 | 23.00 | 11.17 | 26.94 | 9.89 | Week 22 |  |  |
| CBG | 2024-06-01 | 152 | 17.28 | 23.17 | 11.33 | 26.67 | 9.39 | Week 22 |  |  |
| CBG | 2024-06-02 | 153 | 17.44 | 23.39 | 11.50 | 26.67 | 9.22 | Week 22 |  |  |
| CBG | 2024-06-03 | 154 | 17.61 | 23.56 | 11.72 | 25.56 | 7.94 | **Week 22** | **7.103** | **<0.001** |
| CBG | 2024-06-04 | 155 | 17.83 | 23.72 | 11.89 | 21.11 | 3.28 | Week 23 |  |  |
| CBG | 2024-06-05 | 156 | 18.00 | 23.94 | 12.06 | 21.67 | 3.67 | Week 23 |  |  |
| CBG | 2024-06-06 | 157 | 18.22 | 24.11 | 12.28 | 20.56 | 2.33 | Week 23 |  |  |
| CBG | 2024-06-07 | 158 | 18.39 | 24.33 | 12.44 | 18.78 | 0.39 | Week 23 |  |  |
| CBG | 2024-06-08 | 159 | 18.56 | 24.50 | 12.67 | 18.50 | -0.06 | Week 23 |  |  |
| CBG | 2024-06-09 | 160 | 18.78 | 24.72 | 12.83 | 20.28 | 1.50 | Week 23 |  |  |
| CBG | 2024-06-10 | 161 | 18.94 | 24.89 | 13.06 | 24.06 | 5.11 | **Week 23** | **3.312** | **0.016** |
| CBG | 2024-06-11 | 162 | 19.17 | 25.06 | 13.22 | 14.06 | -5.11 | Week 24 |  |  |
| CBG | 2024-06-12 | 163 | 19.33 | 25.28 | 13.44 | 15.83 | -3.50 | Week 24 |  |  |
| CBG | 2024-06-13 | 164 | 19.56 | 25.44 | 13.61 | 16.56 | -3.00 | Week 24 |  |  |
| CBG | 2024-06-14 | 165 | 19.72 | 25.61 | 13.83 | 17.94 | -1.78 | Week 24 |  |  |
| CBG | 2024-06-15 | 166 | 19.89 | 25.78 | 14.00 | 18.17 | -1.72 | Week 24 |  |  |
| CBG | 2024-06-16 | 167 | 20.11 | 26.00 | 14.22 | 15.11 | -5.00 | Week 24 |  |  |
| CBG | 2024-06-17 | 168 | 20.28 | 26.17 | 14.39 | 19.44 | -0.83 | **Week 24** | **-4.770** | **0.003** |
| CBG | 2024-06-18 | 169 | 20.44 | 26.33 | 14.61 | 23.06 | 2.61 | Week 25 |  |  |
| CBG | 2024-06-19 | 170 | 20.61 | 26.50 | 14.78 | 24.00 | 3.39 | Week 25 |  |  |
| CBG | 2024-06-20 | 171 | 20.83 | 26.67 | 14.94 | 23.33 | 2.50 | Week 25 |  |  |
| CBG | 2024-06-21 | 172 | 21.00 | 26.78 | 15.17 | 24.72 | 3.72 | Week 25 |  |  |
| CBG | 2024-06-22 | 173 | 21.11 | 26.94 | 15.33 | 24.56 | 3.44 | Week 25 |  |  |
| CBG | 2024-06-23 | 174 | 21.28 | 27.11 | 15.50 | 24.61 | 3.33 | Week 25 |  |  |
| CBG | 2024-06-24 | 175 | 21.44 | 27.22 | 15.67 | 26.44 | 5.00 | **Week 25** | **10.978** | **<0.001** |
| CBG | 2024-06-25 | 176 | 21.61 | 27.39 | 15.83 | 23.56 | 1.94 | Week 26 |  |  |
| CBG | 2024-06-26 | 177 | 21.72 | 27.50 | 16.00 | 21.89 | 0.17 | Week 26 |  |  |
| CBG | 2024-06-27 | 178 | 21.89 | 27.61 | 16.11 | 20.78 | -1.11 | Week 26 |  |  |
| CBG | 2024-06-28 | 179 | 22.00 | 27.72 | 16.28 | 22.39 | 0.39 | Week 26 |  |  |
| CBG | 2024-06-29 | 180 | 22.11 | 27.83 | 16.39 | 25.72 | 3.61 | Week 26 |  |  |
| CBG | 2024-06-30 | 181 | 22.28 | 27.94 | 16.56 | 26.17 | 3.89 | Week 26 |  |  |
| CBG | 2024-07-01 | 182 | 22.39 | 28.06 | 16.67 | 25.56 | 3.17 | Week 26 | 2.347 | 0.057 |
| CBG | 2024-07-02 | 183 | 22.44 | 28.17 | 16.78 | 20.83 | -1.61 | Week 27 |  |  |
| CBG | 2024-07-03 | 184 | 22.56 | 28.22 | 16.89 | 24.22 | 1.67 | Week 27 |  |  |
| CBG | 2024-07-04 | 185 | 22.67 | 28.33 | 17.00 | 27.89 | 5.22 | Week 27 |  |  |
| CBG | 2024-07-05 | 186 | 22.72 | 28.39 | 17.11 | 25.39 | 2.67 | Week 27 |  |  |
| CBG | 2024-07-06 | 187 | 22.83 | 28.44 | 17.17 | 22.83 | 0.00 | Week 27 |  |  |
| CBG | 2024-07-07 | 188 | 22.89 | 28.50 | 17.22 | 22.61 | -0.28 | Week 27 |  |  |
| CBG | 2024-07-08 | 189 | 22.94 | 28.56 | 17.33 | 20.11 | -2.83 | Week 27 | 0.671 | 0.527 |
| CBG | 2024-07-09 | 190 | 23.00 | 28.61 | 17.39 | 23.06 | 0.06 | Week 28 |  |  |
| CBG | 2024-07-10 | 191 | 23.06 | 28.67 | 17.44 | 24.72 | 1.67 | Week 28 |  |  |
| CBG | 2024-07-11 | 192 | 23.11 | 28.72 | 17.50 | 23.00 | -0.11 | Week 28 |  |  |
| CBG | 2024-07-12 | 193 | 23.11 | 28.72 | 17.56 | 21.06 | -2.06 | Week 28 |  |  |
| CBG | 2024-07-13 | 194 | 23.17 | 28.72 | 17.56 | 21.22 | -1.94 | Week 28 |  |  |
| CBG | 2024-07-14 | 195 | 23.17 | 28.78 | 17.61 | 25.44 | 2.28 | Week 28 |  |  |
| CBG | 2024-07-15 | 196 | 23.22 | 28.78 | 17.61 | 24.44 | 1.22 | Week 28 | 0.247 | 0.813 |
| CBG | 2024-07-16 | 197 | 23.22 | 28.78 | 17.67 | 23.67 | 0.44 | Week 29 |  |  |
| CBG | 2024-07-17 | 198 | 23.22 | 28.78 | 17.67 | 23.06 | -0.17 | Week 29 |  |  |
| CBG | 2024-07-18 | 199 | 23.22 | 28.78 | 17.67 | 21.94 | -1.28 | Week 29 |  |  |
| CBG | 2024-07-19 | 200 | 23.22 | 28.78 | 17.67 | 24.11 | 0.89 | Week 29 |  |  |
| CBG | 2024-07-20 | 201 | 23.22 | 28.78 | 17.67 | 26.00 | 2.78 | Week 29 |  |  |
| CBG | 2024-07-21 | 202 | 23.22 | 28.78 | 17.67 | 23.06 | -0.17 | Week 29 |  |  |
| CBG | 2024-07-22 | 203 | 23.22 | 28.78 | 17.67 | 23.44 | 0.22 | Week 29 | 0.823 | 0.442 |
| CBG | 2024-07-23 | 204 | 23.22 | 28.72 | 17.67 | 23.39 | 0.17 | Week 30 |  |  |
| CBG | 2024-07-24 | 205 | 23.17 | 28.72 | 17.67 | 25.56 | 2.39 | Week 30 |  |  |
| CBG | 2024-07-25 | 206 | 23.17 | 28.72 | 17.61 | 27.00 | 3.83 | Week 30 |  |  |
| CBG | 2024-07-26 | 207 | 23.17 | 28.67 | 17.61 | 28.00 | 4.83 | Week 30 |  |  |
| CBG | 2024-07-27 | 208 | 23.11 | 28.67 | 17.61 | 28.89 | 5.78 | Week 30 |  |  |
| CBG | 2024-07-28 | 209 | 23.11 | 28.61 | 17.56 | 27.06 | 3.94 | Week 30 |  |  |
| CBG | 2024-07-29 | 210 | 23.06 | 28.61 | 17.56 | 23.94 | 0.89 | **Week 30** | **4.009** | **0.007** |
| CBG | 2024-07-30 | 211 | 23.06 | 28.56 | 17.50 | 23.39 | 0.33 | Week 31 |  |  |
| CBG | 2024-07-31 | 212 | 23.00 | 28.56 | 17.50 | 23.33 | 0.33 | Week 31 |  |  |
| CBG | 2024-08-01 | 213 | 23.00 | 28.50 | 17.50 | 25.00 | 2.00 | Week 31 |  |  |
| CBG | 2024-08-02 | 214 | 22.94 | 28.44 | 17.44 | 25.00 | 2.06 | Week 31 |  |  |
| CBG | 2024-08-03 | 215 | 22.94 | 28.44 | 17.44 | 25.39 | 2.44 | Week 31 |  |  |
| CBG | 2024-08-04 | 216 | 22.89 | 28.39 | 17.39 | 25.61 | 2.72 | Week 31 |  |  |
| CBG | 2024-08-05 | 217 | 22.83 | 28.33 | 17.39 | 22.89 | 0.06 | **Week 31** | **3.316** | **0.016** |
| CBG | 2024-08-06 | 218 | 22.83 | 28.33 | 17.33 | 20.28 | -2.56 | Week 32 |  |  |
| CBG | 2024-08-07 | 219 | 22.78 | 28.28 | 17.28 | 22.44 | -0.33 | Week 32 |  |  |
| CBG | 2024-08-08 | 220 | 22.78 | 28.22 | 17.28 | 23.28 | 0.50 | Week 32 |  |  |
| CBG | 2024-08-09 | 221 | 22.72 | 28.22 | 17.22 | 22.56 | -0.17 | Week 32 |  |  |
| CBG | 2024-08-10 | 222 | 22.67 | 28.17 | 17.22 | 23.00 | 0.33 | Week 32 |  |  |
| CBG | 2024-08-11 | 223 | 22.67 | 28.11 | 17.17 | 24.94 | 2.28 | Week 32 |  |  |
| CBG | 2024-08-12 | 224 | 22.61 | 28.06 | 17.11 | 25.72 | 3.11 | Week 32 | 0.648 | 0.541 |
| CBG | 2024-08-13 | 225 | 22.56 | 28.06 | 17.11 | 22.83 | 0.28 | Week 33 |  |  |
| CBG | 2024-08-14 | 226 | 22.50 | 28.00 | 17.06 | 21.06 | -1.44 | Week 33 |  |  |
| CBG | 2024-08-15 | 227 | 22.50 | 27.94 | 17.00 | 20.06 | -2.44 | Week 33 |  |  |
| CBG | 2024-08-16 | 228 | 22.44 | 27.89 | 16.94 | 22.06 | -0.39 | Week 33 |  |  |
| CBG | 2024-08-17 | 229 | 22.39 | 27.83 | 16.94 | 21.44 | -0.94 | Week 33 |  |  |
| CBG | 2024-08-18 | 230 | 22.33 | 27.78 | 16.89 | 20.61 | -1.72 | Week 33 |  |  |
| CBG | 2024-08-19 | 231 | 22.28 | 27.72 | 16.83 | 22.94 | 0.67 | Week 33 | -2.035 | 0.088 |
| CBG | 2024-08-20 | 232 | 22.22 | 27.67 | 16.72 | 26.72 | 4.50 | Week 34 |  |  |
| CBG | 2024-08-21 | 233 | 22.11 | 27.56 | 16.67 | 24.33 | 2.22 | Week 34 |  |  |
| CBG | 2024-08-22 | 234 | 22.06 | 27.50 | 16.61 | 26.56 | 4.50 | Week 34 |  |  |
| CBG | 2024-08-23 | 235 | 22.00 | 27.44 | 16.56 | 30.56 | 8.56 | Week 34 |  |  |
| CBG | 2024-08-24 | 236 | 21.89 | 27.33 | 16.44 | 31.28 | 9.39 | Week 34 |  |  |
| CBG | 2024-08-25 | 237 | 21.83 | 27.28 | 16.39 | 23.89 | 2.06 | Week 34 |  |  |
| CBG | 2024-08-26 | 238 | 21.72 | 27.17 | 16.28 | 22.11 | 0.39 | **Week 34** | **3.538** | **0.012** |
| CBG | 2024-08-27 | 239 | 21.61 | 27.11 | 16.17 | 21.00 | -0.61 | Week 35 |  |  |
| CBG | 2024-08-28 | 240 | 21.50 | 27.00 | 16.06 | 21.67 | 0.17 | Week 35 |  |  |
| CBG | 2024-08-29 | 241 | 21.39 | 26.89 | 15.94 | 22.39 | 1.00 | Week 35 |  |  |
| CBG | 2024-08-30 | 242 | 21.28 | 26.78 | 15.83 | 18.56 | -2.72 | Week 35 |  |  |
| CBG | 2024-08-31 | 243 | 21.17 | 26.67 | 15.67 | 19.11 | -2.06 | Week 35 |  |  |
| CBG | 2024-09-01 | 244 | 21.06 | 26.56 | 15.56 | 22.22 | 1.17 | Week 35 |  |  |
| CBG | 2024-09-02 | 245 | 20.89 | 26.39 | 15.39 | 24.67 | 3.78 | Week 35 | 0.125 | 0.905 |
| CBG | 2024-09-03 | 246 | 20.78 | 26.28 | 15.28 | 27.39 | 6.61 | Week 36 |  |  |
| CBG | 2024-09-04 | 247 | 20.61 | 26.17 | 15.11 | 28.06 | 7.44 | Week 36 |  |  |
| CBG | 2024-09-05 | 248 | 20.44 | 26.00 | 14.94 | 27.89 | 7.44 | Week 36 |  |  |
| CBG | 2024-09-06 | 249 | 20.28 | 25.83 | 14.72 | 23.28 | 3.00 | Week 36 |  |  |
| CBG | 2024-09-07 | 250 | 20.11 | 25.67 | 14.56 | 20.17 | 0.06 | Week 36 |  |  |
| CBG | 2024-09-08 | 251 | 19.94 | 25.56 | 14.39 | 18.39 | -1.56 | Week 36 |  |  |
| CBG | 2024-09-09 | 252 | 19.78 | 25.39 | 14.17 | 18.50 | -1.28 | Week 36 | 2.009 | 0.091 |
| CBG | 2024-09-10 | 253 | 19.56 | 25.17 | 14.00 | 20.50 | 0.94 | Week 37 |  |  |
| CBG | 2024-09-11 | 254 | 19.39 | 25.00 | 13.78 | 17.94 | -1.44 | Week 37 |  |  |
| CBG | 2024-09-12 | 255 | 19.17 | 24.83 | 13.56 | 17.11 | -2.06 | Week 37 |  |  |
| CBG | 2024-09-13 | 256 | 19.00 | 24.67 | 13.33 | 16.44 | -2.56 | Week 37 |  |  |
| CBG | 2024-09-14 | 257 | 18.78 | 24.44 | 13.11 | 17.06 | -1.72 | Week 37 |  |  |
| CBG | 2024-09-15 | 258 | 18.56 | 24.28 | 12.89 | 19.17 | 0.61 | Week 37 |  |  |
| CBG | 2024-09-16 | 259 | 18.33 | 24.06 | 12.61 | 18.39 | 0.06 | Week 37 | -1.673 | 0.145 |
| CBG | 2024-09-17 | 260 | 18.11 | 23.83 | 12.39 | 16.94 | -1.17 | Week 38 |  |  |
| CBG | 2024-09-18 | 261 | 17.89 | 23.61 | 12.17 | 16.83 | -1.06 | Week 38 |  |  |
| CBG | 2024-09-19 | 262 | 17.67 | 23.44 | 11.89 | 16.56 | -1.11 | Week 38 |  |  |
| CBG | 2024-09-20 | 263 | 17.44 | 23.22 | 11.67 | 21.67 | 4.22 | Week 38 |  |  |
| CBG | 2024-09-21 | 264 | 17.22 | 23.00 | 11.39 | 21.44 | 4.22 | Week 38 |  |  |
| CBG | 2024-09-22 | 265 | 16.94 | 22.78 | 11.17 | 22.00 | 5.06 | Week 38 |  |  |
| CBG | 2024-09-23 | 266 | 16.72 | 22.56 | 10.89 | 21.72 | 5.00 | Week 38 | 1.859 | 0.112 |
| CBG | 2024-09-24 | 267 | 16.50 | 22.33 | 10.67 | 20.22 | 3.72 | Week 39 |  |  |
| CBG | 2024-09-25 | 268 | 16.22 | 22.11 | 10.39 | 20.44 | 4.22 | Week 39 |  |  |
| CBG | 2024-09-26 | 269 | 16.00 | 21.83 | 10.17 | 19.83 | 3.83 | Week 39 |  |  |
| CBG | 2024-09-27 | 270 | 15.78 | 21.61 | 9.89 | 18.89 | 3.11 | Week 39 |  |  |
| CBG | 2024-09-28 | 271 | 15.56 | 21.39 | 9.67 | 18.83 | 3.28 | Week 39 |  |  |
| CBG | 2024-09-29 | 272 | 15.28 | 21.17 | 9.44 | 17.94 | 2.67 | Week 39 |  |  |
| CBG | 2024-09-30 | 273 | 15.06 | 20.94 | 9.17 | 21.67 | 6.61 | **Week 39** | **8.028** | **<0.001** |
| CBG | 2024-10-01 | 274 | 14.83 | 20.67 | 8.94 | 22.78 | 7.94 | Week 40 |  |  |
| CBG | 2024-10-02 | 275 | 14.56 | 20.44 | 8.72 | 23.33 | 8.78 | Week 40 |  |  |
| CBG | 2024-10-03 | 276 | 14.33 | 20.22 | 8.50 | 24.72 | 10.39 | Week 40 |  |  |
| CBG | 2024-10-04 | 277 | 14.11 | 20.00 | 8.28 | 24.78 | 10.67 | Week 40 |  |  |
| CBG | 2024-10-05 | 278 | 13.89 | 19.72 | 8.06 | 19.50 | 5.61 | Week 40 |  |  |
| CBG | 2024-10-06 | 279 | 13.67 | 19.50 | 7.83 | 11.50 | -2.17 | Week 40 |  |  |
| CBG | 2024-10-07 | 280 | 13.44 | 19.28 | 7.61 | 10.61 | -2.83 | **Week 40** | **2.542** | **0.044** |
| CBG | 2024-10-08 | 281 | 13.22 | 19.06 | 7.39 | 10.06 | -3.17 | Week 41 |  |  |
| CBG | 2024-10-09 | 282 | 13.00 | 18.78 | 7.17 | 9.94 | -3.06 | Week 41 |  |  |
| CBG | 2024-10-10 | 283 | 12.78 | 18.56 | 7.00 | 10.94 | -1.83 | Week 41 |  |  |
| CBG | 2024-10-11 | 284 | 12.56 | 18.33 | 6.78 | 12.22 | -0.33 | Week 41 |  |  |
| CBG | 2024-10-12 | 285 | 12.33 | 18.06 | 6.56 | 13.72 | 1.39 | Week 41 |  |  |
| CBG | 2024-10-13 | 286 | 12.11 | 17.83 | 6.39 | 13.72 | 1.61 | Week 41 |  |  |
| CBG | 2024-10-14 | 287 | 11.89 | 17.61 | 6.22 | 12.39 | 0.50 | Week 41 | -0.920 | 0.393 |
| CBG | 2024-10-15 | 288 | 11.72 | 17.39 | 6.00 | 10.83 | -0.89 | Week 42 |  |  |
| CBG | 2024-10-16 | 289 | 11.50 | 17.11 | 5.83 | 10.89 | -0.61 | Week 42 |  |  |
| CBG | 2024-10-17 | 290 | 11.28 | 16.89 | 5.67 | 10.94 | -0.33 | Week 42 |  |  |
| CBG | 2024-10-18 | 291 | 11.06 | 16.67 | 5.50 | 13.50 | 2.44 | Week 42 |  |  |
| CBG | 2024-10-19 | 292 | 10.89 | 16.39 | 5.33 | 13.33 | 2.44 | Week 42 |  |  |
| CBG | 2024-10-20 | 293 | 10.67 | 16.17 | 5.17 | 12.22 | 1.56 | Week 42 |  |  |
| CBG | 2024-10-21 | 294 | 10.44 | 15.94 | 5.00 | 11.83 | 1.39 | Week 42 | 1.576 | 0.166 |
| CBG | 2024-10-22 | 295 | 10.22 | 15.67 | 4.78 | 9.83 | -0.39 | Week 43 |  |  |
| CBG | 2024-10-23 | 296 | 10.06 | 15.44 | 4.61 | 13.00 | 2.94 | Week 43 |  |  |
| CBG | 2024-10-24 | 297 | 9.83 | 15.22 | 4.44 | 20.50 | 10.67 | Week 43 |  |  |
| CBG | 2024-10-25 | 298 | 9.61 | 14.94 | 4.28 | 17.61 | 8.00 | Week 43 |  |  |
| CBG | 2024-10-26 | 299 | 9.44 | 14.72 | 4.11 | 19.33 | 9.89 | Week 43 |  |  |
| CBG | 2024-10-27 | 300 | 9.22 | 14.44 | 3.94 | 16.50 | 7.28 | Week 43 |  |  |
| CBG | 2024-10-28 | 301 | 9.00 | 14.22 | 3.78 | 8.00 | -1.00 | **Week 43** | **2.940** | **0.026** |
| CBG | 2024-10-29 | 302 | 8.78 | 13.94 | 3.61 | 7.33 | -1.44 | Week 44 |  |  |
| CBG | 2024-10-30 | 303 | 8.61 | 13.72 | 3.44 | 3.00 | -5.61 | Week 44 |  |  |
| CBG | 2024-10-31 | 304 | 8.39 | 13.44 | 3.28 | 0.83 | -7.56 | Week 44 | -2.702 | 0.114 |
| Mobot | 2024-01-01 | 1 | 0.94 | 5.11 | -3.22 | 12.22 | 11.28 | Week 1 |  |  |
| Mobot | 2024-01-02 | 2 | 0.83 | 5.00 | -3.33 | 13.89 | 13.06 | Week 1 |  |  |
| Mobot | 2024-01-03 | 3 | 0.72 | 4.94 | -3.44 | 14.72 | 14.00 | Week 1 |  |  |
| Mobot | 2024-01-04 | 4 | 0.67 | 4.83 | -3.56 | 5.28 | 4.61 | Week 1 |  |  |
| Mobot | 2024-01-05 | 5 | 0.56 | 4.78 | -3.67 | 3.06 | 2.50 | Week 1 |  |  |
| Mobot | 2024-01-06 | 6 | 0.50 | 4.72 | -3.72 | 3.06 | 2.56 | Week 1 |  |  |
| Mobot | 2024-01-07 | 7 | 0.39 | 4.67 | -3.83 | 2.78 | 2.39 | **Week 1** | **3.571** | **0.012** |
| Mobot | 2024-01-08 | 8 | 0.33 | 4.61 | -3.89 | 0.56 | 0.22 | Week 2 |  |  |
| Mobot | 2024-01-09 | 9 | 0.28 | 4.56 | -4.00 | 4.17 | 3.89 | Week 2 |  |  |
| Mobot | 2024-01-10 | 10 | 0.22 | 4.56 | -4.06 | 9.17 | 8.94 | Week 2 |  |  |
| Mobot | 2024-01-11 | 11 | 0.17 | 4.50 | -4.11 | 12.22 | 12.06 | Week 2 |  |  |
| Mobot | 2024-01-12 | 12 | 0.17 | 4.50 | -4.17 | 5.28 | 5.11 | Week 2 |  |  |
| Mobot | 2024-01-13 | 13 | 0.11 | 4.44 | -4.22 | -3.06 | -3.17 | Week 2 |  |  |
| Mobot | 2024-01-14 | 14 | 0.06 | 4.44 | -4.28 | -1.67 | -1.72 | Week 2 | 1.711 | 0.138 |
| Mobot | 2024-01-15 | 15 | 0.06 | 4.44 | -4.33 | 6.11 | 6.06 | Week 3 |  |  |
| Mobot | 2024-01-16 | 16 | 0.06 | 4.50 | -4.39 | 12.22 | 12.17 | Week 3 |  |  |
| Mobot | 2024-01-17 | 17 | 0.06 | 4.50 | -4.39 | 6.94 | 6.89 | Week 3 |  |  |
| Mobot | 2024-01-18 | 18 | 0.06 | 4.50 | -4.44 | 4.72 | 4.67 | Week 3 |  |  |
| Mobot | 2024-01-19 | 19 | 0.06 | 4.56 | -4.44 | 4.72 | 4.67 | Week 3 |  |  |
| Mobot | 2024-01-20 | 20 | 0.06 | 4.61 | -4.44 | 0.56 | 0.50 | Week 3 |  |  |
| Mobot | 2024-01-21 | 21 | 0.11 | 4.67 | -4.44 | 0.56 | 0.44 | **Week 3** | **3.322** | **0.016** |
| Mobot | 2024-01-22 | 22 | 0.11 | 4.72 | -4.44 | 2.78 | 2.67 | Week 4 |  |  |
| Mobot | 2024-01-23 | 23 | 0.17 | 4.78 | -4.44 | 5.00 | 4.83 | Week 4 |  |  |
| Mobot | 2024-01-24 | 24 | 0.22 | 4.83 | -4.39 | 3.06 | 2.83 | Week 4 |  |  |
| Mobot | 2024-01-25 | 25 | 0.28 | 4.89 | -4.39 | 1.94 | 1.67 | Week 4 |  |  |
| Mobot | 2024-01-26 | 26 | 0.33 | 5.00 | -4.33 | -1.11 | -1.44 | Week 4 |  |  |
| Mobot | 2024-01-27 | 27 | 0.39 | 5.11 | -4.28 | 4.44 | 4.06 | Week 4 |  |  |
| Mobot | 2024-01-28 | 28 | 0.50 | 5.17 | -4.22 | 7.50 | 7.00 | **Week 4** | **3.084** | **0.022** |
| Mobot | 2024-01-29 | 29 | 0.56 | 5.28 | -4.17 | 2.22 | 1.67 | Week 5 |  |  |
| Mobot | 2024-01-30 | 30 | 0.67 | 5.39 | -4.11 | -6.67 | -7.33 | Week 5 |  |  |
| Mobot | 2024-01-31 | 31 | 0.78 | 5.56 | -4.00 | -8.61 | -9.39 | Week 5 |  |  |
| Mobot | 2024-02-01 | 32 | 0.89 | 5.67 | -3.94 | -4.17 | -5.06 | Week 5 |  |  |
| Mobot | 2024-02-02 | 33 | 1.00 | 5.78 | -3.83 | 2.50 | 1.50 | Week 5 |  |  |
| Mobot | 2024-02-03 | 34 | 1.11 | 5.94 | -3.72 | -6.94 | -8.06 | Week 5 |  |  |
| Mobot | 2024-02-04 | 35 | 1.22 | 6.06 | -3.61 | 3.89 | 2.67 | Week 5 | -1.744 | 0.132 |
| Mobot | 2024-02-05 | 36 | 1.33 | 6.22 | -3.50 | 9.72 | 8.39 | Week 6 |  |  |
| Mobot | 2024-02-06 | 37 | 1.50 | 6.39 | -3.39 | 8.33 | 6.83 | Week 6 |  |  |
| Mobot | 2024-02-07 | 38 | 1.61 | 6.56 | -3.28 | 8.89 | 7.28 | Week 6 |  |  |
| Mobot | 2024-02-08 | 39 | 1.78 | 6.72 | -3.11 | 4.44 | 2.67 | Week 6 |  |  |
| Mobot | 2024-02-09 | 40 | 1.94 | 6.89 | -3.00 | 8.33 | 6.39 | Week 6 |  |  |
| Mobot | 2024-02-10 | 41 | 2.11 | 7.06 | -2.83 | 3.06 | 0.94 | Week 6 |  |  |
| Mobot | 2024-02-11 | 42 | 2.28 | 7.22 | -2.67 | 1.94 | -0.33 | **Week 6** | **3.531** | **0.012** |
| Mobot | 2024-02-12 | 43 | 2.44 | 7.39 | -2.56 | 5.83 | 3.39 | Week 7 |  |  |
| Mobot | 2024-02-13 | 44 | 2.61 | 7.56 | -2.39 | 10.28 | 7.67 | Week 7 |  |  |
| Mobot | 2024-02-14 | 45 | 2.78 | 7.72 | -2.22 | 10.28 | 7.50 | Week 7 |  |  |
| Mobot | 2024-02-15 | 46 | 2.94 | 7.89 | -2.06 | 10.00 | 7.06 | Week 7 |  |  |
| Mobot | 2024-02-16 | 47 | 3.11 | 8.11 | -1.83 | 2.22 | -0.89 | Week 7 |  |  |
| Mobot | 2024-02-17 | 48 | 3.28 | 8.28 | -1.67 | -1.39 | -4.67 | Week 7 |  |  |
| Mobot | 2024-02-18 | 49 | 3.50 | 8.44 | -1.50 | 6.11 | 2.61 | Week 7 | 1.825 | 0.118 |
| Mobot | 2024-02-19 | 50 | 3.67 | 8.67 | -1.33 | 10.83 | 7.17 | Week 8 |  |  |
| Mobot | 2024-02-20 | 51 | 3.83 | 8.83 | -1.17 | 10.83 | 7.00 | Week 8 |  |  |
| Mobot | 2024-02-21 | 52 | 4.06 | 9.06 | -0.94 | 8.33 | 4.28 | Week 8 |  |  |
| Mobot | 2024-02-22 | 53 | 4.22 | 9.22 | -0.78 | 16.94 | 12.72 | Week 8 |  |  |
| Mobot | 2024-02-23 | 54 | 4.39 | 9.39 | -0.61 | 6.39 | 2.00 | Week 8 |  |  |
| Mobot | 2024-02-24 | 55 | 4.61 | 9.61 | -0.39 | -0.28 | -4.89 | Week 8 |  |  |
| Mobot | 2024-02-25 | 56 | 4.78 | 9.78 | -0.22 | 5.83 | 1.06 | Week 8 | 1.988 | 0.094 |
| Mobot | 2024-02-26 | 57 | 5.00 | 10.00 | 0.00 | 8.61 | 3.61 | Week 9 |  |  |
| Mobot | 2024-02-27 | 58 | 5.17 | 10.17 | 0.17 | 13.33 | 8.17 | Week 9 |  |  |
| Mobot | 2024-02-28 | 59 | 5.33 | 10.33 | 0.33 | 8.61 | 3.28 | Week 9 |  |  |
| Mobot | 2024-03-01 | 60 | 5.56 | 10.56 | 0.56 | 11.67 | 6.11 | Week 9 |  |  |
| Mobot | 2024-03-02 | 61 | 5.72 | 10.72 | 0.72 | 6.11 | 0.39 | Week 9 |  |  |
| Mobot | 2024-03-03 | 62 | 5.94 | 10.94 | 0.89 | 5.56 | -0.39 | Week 9 |  |  |
| Mobot | 2024-03-04 | 63 | 6.11 | 11.11 | 1.11 | 8.06 | 1.94 | **Week 9** | **2.869** | **0.028** |
| Mobot | 2024-03-05 | 64 | 6.28 | 11.33 | 1.28 | 10.56 | 4.28 | Week 10 |  |  |
| Mobot | 2024-03-06 | 65 | 6.50 | 11.50 | 1.44 | 15.56 | 9.06 | Week 10 |  |  |
| Mobot | 2024-03-07 | 66 | 6.67 | 11.72 | 1.67 | 7.78 | 1.11 | Week 10 |  |  |
| Mobot | 2024-03-08 | 67 | 6.83 | 11.89 | 1.83 | 6.39 | -0.44 | Week 10 |  |  |
| Mobot | 2024-03-09 | 68 | 7.06 | 12.06 | 2.00 | 7.22 | 0.17 | Week 10 |  |  |
| Mobot | 2024-03-10 | 69 | 7.22 | 12.28 | 2.17 | 4.44 | -2.78 | Week 10 |  |  |
| Mobot | 2024-03-11 | 70 | 7.39 | 12.44 | 2.39 | 3.33 | -4.06 | Week 10 | 0.624 | 0.555 |
| Mobot | 2024-03-12 | 71 | 7.61 | 12.67 | 2.56 | 3.89 | -3.72 | Week 11 |  |  |
| Mobot | 2024-03-13 | 72 | 7.78 | 12.89 | 2.72 | 0.56 | -7.22 | Week 11 |  |  |
| Mobot | 2024-03-14 | 73 | 8.00 | 13.06 | 2.89 | 2.50 | -5.50 | Week 11 |  |  |
| Mobot | 2024-03-15 | 74 | 8.17 | 13.28 | 3.06 | 6.11 | -2.06 | Week 11 |  |  |
| Mobot | 2024-03-16 | 75 | 8.33 | 13.44 | 3.22 | 10.56 | 2.22 | Week 11 |  |  |
| Mobot | 2024-03-17 | 76 | 8.56 | 13.67 | 3.39 | 2.78 | -5.78 | Week 11 |  |  |
| Mobot | 2024-03-18 | 77 | 8.72 | 13.83 | 3.56 | -1.67 | -10.39 | **Week 11** | **-3.060** | **0.022** |
| Mobot | 2024-03-19 | 78 | 8.89 | 14.06 | 3.78 | -0.28 | -9.17 | Week 12 |  |  |
| Mobot | 2024-03-20 | 79 | 9.11 | 14.28 | 3.94 | 6.94 | -2.17 | Week 12 |  |  |
| Mobot | 2024-03-21 | 80 | 9.28 | 14.44 | 4.11 | 8.33 | -0.94 | Week 12 |  |  |
| Mobot | 2024-03-22 | 81 | 9.50 | 14.67 | 4.28 | 13.61 | 4.11 | Week 12 |  |  |
| Mobot | 2024-03-23 | 82 | 9.67 | 14.89 | 4.44 | 13.61 | 3.94 | Week 12 |  |  |
| Mobot | 2024-03-24 | 83 | 9.83 | 15.11 | 4.61 | 6.39 | -3.44 | Week 12 |  |  |
| Mobot | 2024-03-25 | 84 | 10.06 | 15.33 | 4.78 | 10.56 | 0.50 | Week 12 | -0.589 | 0.577 |
| Mobot | 2024-03-26 | 85 | 10.22 | 15.50 | 4.94 | 13.33 | 3.11 | Week 13 |  |  |
| Mobot | 2024-03-27 | 86 | 10.44 | 15.72 | 5.17 | 8.61 | -1.83 | Week 13 |  |  |
| Mobot | 2024-03-28 | 87 | 10.61 | 15.94 | 5.33 | 6.11 | -4.50 | Week 13 |  |  |
| Mobot | 2024-03-29 | 88 | 10.83 | 16.17 | 5.50 | 8.06 | -2.78 | Week 13 |  |  |
| Mobot | 2024-03-30 | 89 | 11.06 | 16.39 | 5.67 | 12.78 | 1.72 | Week 13 |  |  |
| Mobot | 2024-03-31 | 90 | 11.22 | 16.61 | 5.89 | 19.72 | 8.50 | Week 13 |  |  |
| Mobot | 2024-04-01 | 91 | 11.44 | 16.83 | 6.06 | 10.56 | -0.89 | Week 13 | 0.287 | 0.784 |
| Mobot | 2024-04-02 | 92 | 11.61 | 17.06 | 6.22 | 12.78 | 1.17 | Week 14 |  |  |
| Mobot | 2024-04-03 | 93 | 11.83 | 17.22 | 6.44 | 19.17 | 7.33 | Week 14 |  |  |
| Mobot | 2024-04-04 | 94 | 12.06 | 17.44 | 6.61 | 23.89 | 11.83 | Week 14 |  |  |
| Mobot | 2024-04-05 | 95 | 12.22 | 17.67 | 6.78 | 16.39 | 4.17 | Week 14 |  |  |
| Mobot | 2024-04-06 | 96 | 12.44 | 17.89 | 7.00 | 8.06 | -4.39 | Week 14 |  |  |
| Mobot | 2024-04-07 | 97 | 12.67 | 18.11 | 7.17 | 9.17 | -3.50 | Week 14 |  |  |
| Mobot | 2024-04-08 | 98 | 12.83 | 18.33 | 7.39 | 11.67 | -1.17 | Week 14 | 0.984 | 0.363 |
| Mobot | 2024-04-09 | 99 | 13.06 | 18.56 | 7.56 | 13.61 | 0.56 | Week 15 |  |  |
| Mobot | 2024-04-10 | 100 | 13.28 | 18.78 | 7.78 | 15.56 | 2.28 | Week 15 |  |  |
| Mobot | 2024-04-11 | 101 | 13.44 | 19.00 | 7.94 | 19.72 | 6.28 | Week 15 |  |  |
| Mobot | 2024-04-12 | 102 | 13.67 | 19.17 | 8.17 | 21.39 | 7.72 | Week 15 |  |  |
| Mobot | 2024-04-13 | 103 | 13.89 | 19.39 | 8.33 | 18.89 | 5.00 | Week 15 |  |  |
| Mobot | 2024-04-14 | 104 | 14.06 | 19.61 | 8.56 | 20.56 | 6.50 | Week 15 |  |  |
| Mobot | 2024-04-15 | 105 | 14.28 | 19.83 | 8.78 | 18.33 | 4.06 | **Week 15** | **4.841** | **0.003** |
| Mobot | 2024-04-16 | 106 | 14.50 | 20.00 | 8.94 | 8.33 | -6.17 | Week 16 |  |  |
| Mobot | 2024-04-17 | 107 | 14.67 | 20.22 | 9.17 | 13.06 | -1.61 | Week 16 |  |  |
| Mobot | 2024-04-18 | 108 | 14.89 | 20.39 | 9.39 | 14.72 | -0.17 | Week 16 |  |  |
| Mobot | 2024-04-19 | 109 | 15.11 | 20.61 | 9.56 | 21.11 | 6.00 | Week 16 |  |  |
| Mobot | 2024-04-20 | 110 | 15.28 | 20.78 | 9.78 | 19.44 | 4.17 | Week 16 |  |  |
| Mobot | 2024-04-21 | 111 | 15.50 | 21.00 | 10.00 | 14.44 | -1.06 | Week 16 |  |  |
| Mobot | 2024-04-22 | 112 | 15.67 | 21.17 | 10.17 | 19.17 | 3.50 | Week 16 | 0.423 | 0.687 |
| Mobot | 2024-04-23 | 113 | 15.89 | 21.39 | 10.39 | 8.61 | -7.28 | Week 17 |  |  |
| Mobot | 2024-04-24 | 114 | 16.06 | 21.56 | 10.61 | 10.00 | -6.06 | Week 17 |  |  |
| Mobot | 2024-04-25 | 115 | 16.28 | 21.72 | 10.78 | 16.11 | -0.17 | Week 17 |  |  |
| Mobot | 2024-04-26 | 116 | 16.44 | 21.89 | 11.00 | 12.50 | -3.94 | Week 17 |  |  |
| Mobot | 2024-04-27 | 117 | 16.67 | 22.06 | 11.22 | 14.44 | -2.22 | Week 17 |  |  |
| Mobot | 2024-04-28 | 118 | 16.83 | 22.28 | 11.39 | 17.78 | 0.94 | Week 17 |  |  |
| Mobot | 2024-04-29 | 119 | 17.00 | 22.44 | 11.61 | 14.44 | -2.56 | **Week 17** | **-2.708** | **0.035** |
| Mobot | 2024-04-30 | 120 | 17.22 | 22.61 | 11.83 | 10.28 | -6.94 | Week 18 |  |  |
| Mobot | 2024-05-01 | 121 | 17.39 | 22.78 | 12.00 | 13.89 | -3.50 | Week 18 |  |  |
| Mobot | 2024-05-02 | 122 | 17.56 | 22.94 | 12.22 | 13.89 | -3.67 | Week 18 |  |  |
| Mobot | 2024-05-03 | 123 | 17.72 | 23.11 | 12.39 | 13.61 | -4.11 | Week 18 |  |  |
| Mobot | 2024-05-04 | 124 | 17.94 | 23.28 | 12.61 | 16.11 | -1.83 | Week 18 |  |  |
| Mobot | 2024-05-05 | 125 | 18.11 | 23.39 | 12.78 | 18.89 | 0.78 | Week 18 |  |  |
| Mobot | 2024-05-06 | 126 | 18.28 | 23.56 | 13.00 | 22.22 | 3.94 | Week 18 | -1.620 | 0.156 |
| Mobot | 2024-05-07 | 127 | 18.44 | 23.72 | 13.17 | 26.11 | 7.67 | Week 19 |  |  |
| Mobot | 2024-05-08 | 128 | 18.61 | 23.89 | 13.33 | 23.89 | 5.28 | Week 19 |  |  |
| Mobot | 2024-05-09 | 129 | 18.78 | 24.06 | 13.56 | 20.00 | 1.22 | Week 19 |  |  |
| Mobot | 2024-05-10 | 130 | 18.94 | 24.22 | 13.72 | 22.50 | 3.56 | Week 19 |  |  |
| Mobot | 2024-05-11 | 131 | 19.11 | 24.33 | 13.94 | 22.22 | 3.11 | Week 19 |  |  |
| Mobot | 2024-05-12 | 132 | 19.28 | 24.50 | 14.11 | 24.17 | 4.89 | Week 19 |  |  |
| Mobot | 2024-05-13 | 133 | 19.50 | 24.67 | 14.28 | 26.11 | 6.61 | **Week 19** | **5.587** | **0.001** |
| Mobot | 2024-05-14 | 134 | 19.67 | 24.83 | 14.44 | 20.56 | 0.89 | Week 20 |  |  |
| Mobot | 2024-05-15 | 135 | 19.83 | 25.00 | 14.67 | 18.06 | -1.78 | Week 20 |  |  |
| Mobot | 2024-05-16 | 136 | 20.00 | 25.17 | 14.83 | 21.11 | 1.11 | Week 20 |  |  |
| Mobot | 2024-05-17 | 137 | 20.17 | 25.28 | 15.00 | 22.50 | 2.33 | Week 20 |  |  |
| Mobot | 2024-05-18 | 138 | 20.33 | 25.44 | 15.17 | 21.39 | 1.06 | Week 20 |  |  |
| Mobot | 2024-05-19 | 139 | 20.50 | 25.61 | 15.33 | 17.22 | -3.28 | Week 20 |  |  |
| Mobot | 2024-05-20 | 140 | 20.67 | 25.78 | 15.50 | 18.33 | -2.33 | Week 20 | -0.354 | 0.736 |
| Mobot | 2024-05-21 | 141 | 20.83 | 25.94 | 15.72 | 18.06 | -2.78 | Week 21 |  |  |
| Mobot | 2024-05-22 | 142 | 21.00 | 26.11 | 15.89 | 20.00 | -1.00 | Week 21 |  |  |
| Mobot | 2024-05-23 | 143 | 21.17 | 26.28 | 16.06 | 21.67 | 0.50 | Week 21 |  |  |
| Mobot | 2024-05-24 | 144 | 21.33 | 26.44 | 16.22 | 25.28 | 3.94 | Week 21 |  |  |
| Mobot | 2024-05-25 | 145 | 21.50 | 26.61 | 16.39 | 22.50 | 1.00 | Week 21 |  |  |
| Mobot | 2024-05-26 | 146 | 21.67 | 26.78 | 16.56 | 18.89 | -2.78 | Week 21 |  |  |
| Mobot | 2024-05-27 | 147 | 21.83 | 26.94 | 16.72 | 20.56 | -1.28 | Week 21 | -0.379 | 0.718 |
| Mobot | 2024-05-28 | 148 | 22.00 | 27.11 | 16.89 | 21.67 | -0.33 | Week 22 |  |  |
| Mobot | 2024-05-29 | 149 | 22.17 | 27.28 | 17.06 | 23.06 | 0.89 | Week 22 |  |  |
| Mobot | 2024-05-30 | 150 | 22.33 | 27.44 | 17.22 | 24.44 | 2.11 | Week 22 |  |  |
| Mobot | 2024-05-31 | 151 | 22.50 | 27.67 | 17.39 | 26.67 | 4.17 | Week 22 |  |  |
| Mobot | 2024-06-01 | 152 | 22.67 | 27.83 | 17.56 | 27.50 | 4.83 | Week 22 |  |  |
| Mobot | 2024-06-02 | 153 | 22.83 | 28.00 | 17.72 | 27.78 | 4.94 | Week 22 |  |  |
| Mobot | 2024-06-03 | 154 | 23.00 | 28.17 | 17.89 | 26.39 | 3.39 | **Week 22** | **3.713** | **0.010** |
| Mobot | 2024-06-04 | 155 | 23.17 | 28.33 | 18.06 | 26.94 | 3.78 | Week 23 |  |  |
| Mobot | 2024-06-05 | 156 | 23.33 | 28.50 | 18.22 | 23.89 | 0.56 | Week 23 |  |  |
| Mobot | 2024-06-06 | 157 | 23.50 | 28.67 | 18.33 | 24.61 | 1.11 | Week 23 |  |  |
| Mobot | 2024-06-07 | 158 | 23.67 | 28.83 | 18.50 | 23.06 | -0.61 | Week 23 |  |  |
| Mobot | 2024-06-08 | 159 | 23.83 | 29.00 | 18.67 | 21.22 | -2.61 | Week 23 |  |  |
| Mobot | 2024-06-09 | 160 | 24.00 | 29.17 | 18.83 | 21.89 | -2.11 | Week 23 |  |  |
| Mobot | 2024-06-10 | 161 | 24.17 | 29.33 | 19.00 | 24.83 | 0.67 | Week 23 | 0.137 | 0.896 |
| Mobot | 2024-06-11 | 162 | 24.33 | 29.50 | 19.11 | 20.22 | -4.11 | Week 24 |  |  |
| Mobot | 2024-06-12 | 163 | 24.44 | 29.67 | 19.28 | 18.11 | -6.33 | Week 24 |  |  |
| Mobot | 2024-06-13 | 164 | 24.61 | 29.78 | 19.44 | 22.39 | -2.22 | Week 24 |  |  |
| Mobot | 2024-06-14 | 165 | 24.78 | 29.94 | 19.56 | 25.28 | 0.50 | Week 24 |  |  |
| Mobot | 2024-06-15 | 166 | 24.89 | 30.11 | 19.72 | 26.61 | 1.72 | Week 24 |  |  |
| Mobot | 2024-06-16 | 167 | 25.06 | 30.22 | 19.83 | 22.94 | -2.11 | Week 24 |  |  |
| Mobot | 2024-06-17 | 168 | 25.17 | 30.33 | 20.00 | 24.00 | -1.17 | Week 24 | -1.913 | 0.104 |
| Mobot | 2024-06-18 | 169 | 25.28 | 30.50 | 20.11 | 22.39 | -2.89 | Week 25 |  |  |
| Mobot | 2024-06-19 | 170 | 25.44 | 30.61 | 20.22 | 23.56 | -1.89 | Week 25 |  |  |
| Mobot | 2024-06-20 | 171 | 25.56 | 30.72 | 20.39 | 26.72 | 1.17 | Week 25 |  |  |
| Mobot | 2024-06-21 | 172 | 25.67 | 30.83 | 20.50 | 26.11 | 0.44 | Week 25 |  |  |
| Mobot | 2024-06-22 | 173 | 25.78 | 30.94 | 20.61 | 26.28 | 0.50 | Week 25 |  |  |
| Mobot | 2024-06-23 | 174 | 25.89 | 31.06 | 20.72 | 27.00 | 1.11 | Week 25 |  |  |
| Mobot | 2024-06-24 | 175 | 26.00 | 31.17 | 20.83 | 30.17 | 4.17 | Week 25 | 0.432 | 0.681 |
| Mobot | 2024-06-25 | 176 | 26.11 | 31.28 | 20.94 | 30.61 | 4.50 | Week 26 |  |  |
| Mobot | 2024-06-26 | 177 | 26.17 | 31.33 | 21.06 | 26.00 | -0.17 | Week 26 |  |  |
| Mobot | 2024-06-27 | 178 | 26.28 | 31.44 | 21.11 | 25.94 | -0.33 | Week 26 |  |  |
| Mobot | 2024-06-28 | 179 | 26.39 | 31.50 | 21.22 | 27.67 | 1.28 | Week 26 |  |  |
| Mobot | 2024-06-29 | 180 | 26.44 | 31.56 | 21.33 | 28.83 | 2.39 | Week 26 |  |  |
| Mobot | 2024-06-30 | 181 | 26.50 | 31.67 | 21.39 | 28.44 | 1.94 | Week 26 |  |  |
| Mobot | 2024-07-01 | 182 | 26.61 | 31.72 | 21.44 | 24.83 | -1.78 | Week 26 | 1.425 | 0.204 |
| Mobot | 2024-07-02 | 183 | 26.67 | 31.78 | 21.56 | 23.72 | -2.94 | Week 27 |  |  |
| Mobot | 2024-07-03 | 184 | 26.72 | 31.83 | 21.61 | 27.28 | 0.56 | Week 27 |  |  |
| Mobot | 2024-07-04 | 185 | 26.78 | 31.83 | 21.67 | 29.22 | 2.44 | Week 27 |  |  |
| Mobot | 2024-07-05 | 186 | 26.83 | 31.89 | 21.72 | 29.22 | 2.39 | Week 27 |  |  |
| Mobot | 2024-07-06 | 187 | 26.89 | 31.94 | 21.78 | 25.61 | -1.28 | Week 27 |  |  |
| Mobot | 2024-07-07 | 188 | 26.89 | 31.94 | 21.83 | 25.06 | -1.83 | Week 27 |  |  |
| Mobot | 2024-07-08 | 189 | 26.94 | 32.00 | 21.89 | 23.39 | -3.56 | Week 27 | -0.654 | 0.538 |
| Mobot | 2024-07-09 | 190 | 27.00 | 32.00 | 21.94 | 23.78 | -3.22 | Week 28 |  |  |
| Mobot | 2024-07-10 | 191 | 27.00 | 32.00 | 22.00 | 26.78 | -0.22 | Week 28 |  |  |
| Mobot | 2024-07-11 | 192 | 27.06 | 32.06 | 22.00 | 29.00 | 1.94 | Week 28 |  |  |
| Mobot | 2024-07-12 | 193 | 27.06 | 32.06 | 22.06 | 28.44 | 1.39 | Week 28 |  |  |
| Mobot | 2024-07-13 | 194 | 27.06 | 32.06 | 22.06 | 26.00 | -1.06 | Week 28 |  |  |
| Mobot | 2024-07-14 | 195 | 27.11 | 32.06 | 22.11 | 27.67 | 0.56 | Week 28 |  |  |
| Mobot | 2024-07-15 | 196 | 27.11 | 32.06 | 22.11 | 25.72 | -1.39 | Week 28 | -0.426 | 0.685 |
| Mobot | 2024-07-16 | 197 | 27.11 | 32.06 | 22.11 | 26.28 | -0.83 | Week 29 |  |  |
| Mobot | 2024-07-17 | 198 | 27.11 | 32.06 | 22.17 | 25.89 | -1.22 | Week 29 |  |  |
| Mobot | 2024-07-18 | 199 | 27.11 | 32.06 | 22.17 | 25.33 | -1.78 | Week 29 |  |  |
| Mobot | 2024-07-19 | 200 | 27.11 | 32.06 | 22.17 | 26.28 | -0.83 | Week 29 |  |  |
| Mobot | 2024-07-20 | 201 | 27.11 | 32.06 | 22.17 | 27.28 | 0.17 | Week 29 |  |  |
| Mobot | 2024-07-21 | 202 | 27.11 | 32.06 | 22.17 | 23.72 | -3.39 | Week 29 |  |  |
| Mobot | 2024-07-22 | 203 | 27.11 | 32.06 | 22.17 | 24.89 | -2.22 | **Week 29** | **-3.333** | **0.016** |
| Mobot | 2024-07-23 | 204 | 27.11 | 32.06 | 22.17 | 27.22 | 0.11 | Week 30 |  |  |
| Mobot | 2024-07-24 | 205 | 27.11 | 32.06 | 22.17 | 29.06 | 1.94 | Week 30 |  |  |
| Mobot | 2024-07-25 | 206 | 27.11 | 32.06 | 22.17 | 30.78 | 3.67 | Week 30 |  |  |
| Mobot | 2024-07-26 | 207 | 27.11 | 32.06 | 22.11 | 31.17 | 4.06 | Week 30 |  |  |
| Mobot | 2024-07-27 | 208 | 27.06 | 32.06 | 22.11 | 32.94 | 5.89 | Week 30 |  |  |
| Mobot | 2024-07-28 | 209 | 27.06 | 32.00 | 22.11 | 33.72 | 6.67 | Week 30 |  |  |
| Mobot | 2024-07-29 | 210 | 27.06 | 32.00 | 22.06 | 28.22 | 1.17 | **Week 30** | **3.663** | **0.011** |
| Mobot | 2024-07-30 | 211 | 27.06 | 32.00 | 22.06 | 27.39 | 0.33 | Week 31 |  |  |
| Mobot | 2024-07-31 | 212 | 27.00 | 32.00 | 22.06 | 25.50 | -1.50 | Week 31 |  |  |
| Mobot | 2024-08-01 | 213 | 27.00 | 32.00 | 22.00 | 25.83 | -1.17 | Week 31 |  |  |
| Mobot | 2024-08-02 | 214 | 26.94 | 31.94 | 22.00 | 24.28 | -2.67 | Week 31 |  |  |
| Mobot | 2024-08-03 | 215 | 26.94 | 31.94 | 21.94 | 25.67 | -1.28 | Week 31 |  |  |
| Mobot | 2024-08-04 | 216 | 26.94 | 31.94 | 21.89 | 26.94 | 0.00 | Week 31 |  |  |
| Mobot | 2024-08-05 | 217 | 26.89 | 31.89 | 21.89 | 25.94 | -0.94 | **Week 31** | **-2.751** | **0.033** |
| Mobot | 2024-08-06 | 218 | 26.83 | 31.89 | 21.83 | 25.83 | -1.00 | Week 32 |  |  |
| Mobot | 2024-08-07 | 219 | 26.83 | 31.89 | 21.78 | 24.22 | -2.61 | Week 32 |  |  |
| Mobot | 2024-08-08 | 220 | 26.78 | 31.83 | 21.72 | 25.56 | -1.22 | Week 32 |  |  |
| Mobot | 2024-08-09 | 221 | 26.78 | 31.83 | 21.67 | 24.00 | -2.78 | Week 32 |  |  |
| Mobot | 2024-08-10 | 222 | 26.72 | 31.78 | 21.61 | 25.11 | -1.61 | Week 32 |  |  |
| Mobot | 2024-08-11 | 223 | 26.67 | 31.78 | 21.56 | 28.11 | 1.44 | Week 32 |  |  |
| Mobot | 2024-08-12 | 224 | 26.61 | 31.72 | 21.50 | 26.61 | 0.00 | Week 32 | -1.992 | 0.093 |
| Mobot | 2024-08-13 | 225 | 26.56 | 31.67 | 21.44 | 24.72 | -1.83 | Week 33 |  |  |
| Mobot | 2024-08-14 | 226 | 26.50 | 31.67 | 21.39 | 24.06 | -2.44 | Week 33 |  |  |
| Mobot | 2024-08-15 | 227 | 26.44 | 31.61 | 21.28 | 20.72 | -5.72 | Week 33 |  |  |
| Mobot | 2024-08-16 | 228 | 26.39 | 31.56 | 21.22 | 23.17 | -3.22 | Week 33 |  |  |
| Mobot | 2024-08-17 | 229 | 26.33 | 31.50 | 21.17 | 24.17 | -2.17 | Week 33 |  |  |
| Mobot | 2024-08-18 | 230 | 26.28 | 31.44 | 21.06 | 22.89 | -3.39 | Week 33 |  |  |
| Mobot | 2024-08-19 | 231 | 26.17 | 31.39 | 20.94 | 26.28 | 0.11 | **Week 33** | **-3.978** | **0.007** |
| Mobot | 2024-08-20 | 232 | 26.11 | 31.33 | 20.89 | 30.28 | 4.17 | Week 34 |  |  |
| Mobot | 2024-08-21 | 233 | 26.00 | 31.22 | 20.78 | 29.67 | 3.67 | Week 34 |  |  |
| Mobot | 2024-08-22 | 234 | 25.89 | 31.17 | 20.67 | 30.50 | 4.61 | Week 34 |  |  |
| Mobot | 2024-08-23 | 235 | 25.83 | 31.06 | 20.56 | 32.39 | 6.56 | Week 34 |  |  |
| Mobot | 2024-08-24 | 236 | 25.72 | 31.00 | 20.44 | 32.94 | 7.22 | Week 34 |  |  |
| Mobot | 2024-08-25 | 237 | 25.61 | 30.89 | 20.33 | 33.72 | 8.11 | Week 34 |  |  |
| Mobot | 2024-08-26 | 238 | 25.50 | 30.78 | 20.22 | 25.44 | -0.06 | **Week 34** | **4.723** | **0.003** |
| Mobot | 2024-08-27 | 239 | 25.39 | 30.67 | 20.06 | 22.06 | -3.33 | Week 35 |  |  |
| Mobot | 2024-08-28 | 240 | 25.28 | 30.56 | 19.94 | 23.78 | -1.50 | Week 35 |  |  |
| Mobot | 2024-08-29 | 241 | 25.11 | 30.44 | 19.78 | 24.61 | -0.50 | Week 35 |  |  |
| Mobot | 2024-08-30 | 242 | 25.00 | 30.33 | 19.67 | 23.22 | -1.78 | Week 35 |  |  |
| Mobot | 2024-08-31 | 243 | 24.83 | 30.22 | 19.50 | 20.83 | -4.00 | Week 35 |  |  |
| Mobot | 2024-09-01 | 244 | 24.72 | 30.06 | 19.33 | 22.33 | -2.39 | Week 35 |  |  |
| Mobot | 2024-09-02 | 245 | 24.56 | 29.94 | 19.17 | 25.67 | 1.11 | **Week 35** | **-2.720** | **0.035** |
| Mobot | 2024-09-03 | 246 | 24.39 | 29.78 | 19.00 | 28.00 | 3.61 | Week 36 |  |  |
| Mobot | 2024-09-04 | 247 | 24.22 | 29.67 | 18.83 | 27.56 | 3.33 | Week 36 |  |  |
| Mobot | 2024-09-05 | 248 | 24.06 | 29.50 | 18.67 | 26.06 | 2.00 | Week 36 |  |  |
| Mobot | 2024-09-06 | 249 | 23.89 | 29.33 | 18.50 | 24.72 | 0.83 | Week 36 |  |  |
| Mobot | 2024-09-07 | 250 | 23.72 | 29.17 | 18.28 | 20.89 | -2.83 | Week 36 |  |  |
| Mobot | 2024-09-08 | 251 | 23.56 | 29.00 | 18.11 | 22.83 | -0.72 | Week 36 |  |  |
| Mobot | 2024-09-09 | 252 | 23.39 | 28.83 | 17.89 | 21.00 | -2.39 | Week 36 | 0.554 | 0.599 |
| Mobot | 2024-09-10 | 253 | 23.17 | 28.67 | 17.72 | 22.72 | -0.44 | Week 37 |  |  |
| Mobot | 2024-09-11 | 254 | 23.00 | 28.44 | 17.50 | 22.33 | -0.67 | Week 37 |  |  |
| Mobot | 2024-09-12 | 255 | 22.78 | 28.28 | 17.33 | 20.22 | -2.56 | Week 37 |  |  |
| Mobot | 2024-09-13 | 256 | 22.61 | 28.11 | 17.11 | 20.89 | -1.72 | Week 37 |  |  |
| Mobot | 2024-09-14 | 257 | 22.39 | 27.89 | 16.89 | 21.22 | -1.17 | Week 37 |  |  |
| Mobot | 2024-09-15 | 258 | 22.17 | 27.72 | 16.67 | 20.56 | -1.61 | Week 37 |  |  |
| Mobot | 2024-09-16 | 259 | 22.00 | 27.50 | 16.50 | 20.22 | -1.78 | **Week 37** | **-5.205** | **0.002** |
| Mobot | 2024-09-17 | 260 | 21.78 | 27.28 | 16.28 | 19.33 | -2.44 | Week 38 |  |  |
| Mobot | 2024-09-18 | 261 | 21.56 | 27.11 | 16.06 | 20.17 | -1.39 | Week 38 |  |  |
| Mobot | 2024-09-19 | 262 | 21.33 | 26.89 | 15.83 | 23.67 | 2.33 | Week 38 |  |  |
| Mobot | 2024-09-20 | 263 | 21.17 | 26.67 | 15.61 | 21.33 | 0.17 | Week 38 |  |  |
| Mobot | 2024-09-21 | 264 | 20.94 | 26.50 | 15.39 | 22.44 | 1.50 | Week 38 |  |  |
| Mobot | 2024-09-22 | 265 | 20.72 | 26.28 | 15.17 | 24.83 | 4.11 | Week 38 |  |  |
| Mobot | 2024-09-23 | 266 | 20.50 | 26.06 | 14.94 | 25.17 | 4.67 | Week 38 | 1.265 | 0.253 |
| Mobot | 2024-09-24 | 267 | 20.28 | 25.83 | 14.72 | 24.39 | 4.11 | Week 39 |  |  |
| Mobot | 2024-09-25 | 268 | 20.06 | 25.61 | 14.50 | 25.17 | 5.11 | Week 39 |  |  |
| Mobot | 2024-09-26 | 269 | 19.83 | 25.44 | 14.28 | 23.89 | 4.06 | Week 39 |  |  |
| Mobot | 2024-09-27 | 270 | 19.61 | 25.22 | 14.06 | 21.22 | 1.61 | Week 39 |  |  |
| Mobot | 2024-09-28 | 271 | 19.39 | 25.00 | 13.83 | 22.78 | 3.39 | Week 39 |  |  |
| Mobot | 2024-09-29 | 272 | 19.17 | 24.78 | 13.61 | 24.61 | 5.44 | Week 39 |  |  |
| Mobot | 2024-09-30 | 273 | 18.94 | 24.56 | 13.39 | 25.67 | 6.72 | **Week 39** | **7.052** | **<0.001** |
| Mobot | 2024-10-01 | 274 | 18.78 | 24.33 | 13.17 | 23.89 | 5.11 | Week 40 |  |  |
| Mobot | 2024-10-02 | 275 | 18.56 | 24.11 | 12.94 | 24.06 | 5.50 | Week 40 |  |  |
| Mobot | 2024-10-03 | 276 | 18.33 | 23.89 | 12.72 | 26.11 | 7.78 | Week 40 |  |  |
| Mobot | 2024-10-04 | 277 | 18.11 | 23.67 | 12.50 | 21.83 | 3.72 | Week 40 |  |  |
| Mobot | 2024-10-05 | 278 | 17.89 | 23.44 | 12.28 | 20.61 | 2.72 | Week 40 |  |  |
| Mobot | 2024-10-06 | 279 | 17.67 | 23.22 | 12.06 | 14.83 | -2.83 | Week 40 |  |  |
| Mobot | 2024-10-07 | 280 | 17.44 | 23.06 | 11.83 | 12.00 | -5.44 | Week 40 | 1.311 | 0.238 |
| Mobot | 2024-10-08 | 281 | 17.22 | 22.83 | 11.67 | 14.78 | -2.44 | Week 41 |  |  |
| Mobot | 2024-10-09 | 282 | 17.00 | 22.61 | 11.44 | 14.39 | -2.61 | Week 41 |  |  |
| Mobot | 2024-10-10 | 283 | 16.78 | 22.39 | 11.22 | 14.17 | -2.61 | Week 41 |  |  |
| Mobot | 2024-10-11 | 284 | 16.56 | 22.11 | 11.00 | 18.44 | 1.89 | Week 41 |  |  |
| Mobot | 2024-10-12 | 285 | 16.33 | 21.89 | 10.78 | 21.06 | 4.72 | Week 41 |  |  |
| Mobot | 2024-10-13 | 286 | 16.17 | 21.67 | 10.61 | 18.78 | 2.61 | Week 41 |  |  |
| Mobot | 2024-10-14 | 287 | 15.94 | 21.44 | 10.39 | 13.22 | -2.72 | Week 41 | -0.140 | 0.893 |
| Mobot | 2024-10-15 | 288 | 15.72 | 21.22 | 10.17 | 12.06 | -3.67 | Week 42 |  |  |
| Mobot | 2024-10-16 | 289 | 15.50 | 21.00 | 10.00 | 11.33 | -4.17 | Week 42 |  |  |
| Mobot | 2024-10-17 | 290 | 15.28 | 20.78 | 9.78 | 13.06 | -2.22 | Week 42 |  |  |
| Mobot | 2024-10-18 | 291 | 15.06 | 20.56 | 9.56 | 17.39 | 2.33 | Week 42 |  |  |
| Mobot | 2024-10-19 | 292 | 14.83 | 20.28 | 9.33 | 16.61 | 1.78 | Week 42 |  |  |
| Mobot | 2024-10-20 | 293 | 14.61 | 20.06 | 9.17 | 16.00 | 1.39 | Week 42 |  |  |
| Mobot | 2024-10-21 | 294 | 14.39 | 19.83 | 8.94 | 17.83 | 3.44 | Week 42 | -0.135 | 0.897 |
| Mobot | 2024-10-22 | 295 | 14.17 | 19.61 | 8.78 | 13.39 | -0.78 | Week 43 |  |  |
| Mobot | 2024-10-23 | 296 | 13.94 | 19.33 | 8.56 | 18.61 | 4.67 | Week 43 |  |  |
| Mobot | 2024-10-24 | 297 | 13.72 | 19.11 | 8.33 | 21.33 | 7.61 | Week 43 |  |  |
| Mobot | 2024-10-25 | 298 | 13.50 | 18.83 | 8.17 | 21.33 | 7.83 | Week 43 |  |  |
| Mobot | 2024-10-26 | 299 | 13.28 | 18.61 | 7.94 | 22.39 | 9.11 | Week 43 |  |  |
| Mobot | 2024-10-27 | 300 | 13.06 | 18.39 | 7.72 | 17.94 | 4.89 | Week 43 |  |  |
| Mobot | 2024-10-28 | 301 | 12.83 | 18.11 | 7.56 | 7.11 | -5.72 | Week 43 | 1.947 | 0.100 |
| Mobot | 2024-10-29 | 302 | 12.61 | 17.83 | 7.33 | 6.83 | -5.78 | Week 44 |  |  |
| Mobot | 2024-10-30 | 303 | 12.39 | 17.61 | 7.11 | 3.39 | -9.00 | Week 44 |  |  |
| Mobot | 2024-10-31 | 304 | 12.17 | 17.33 | 6.94 | 2.22 | -9.94 | **Week 44** | **-6.534** | **0.023** |
| Dawes | 2024-01-01 | 1 | -0.33 | 4.11 | -4.83 | 7.50 | 7.83 | Week 1 |  |  |
| Dawes | 2024-01-02 | 2 | -0.44 | 4.06 | -4.94 | 7.22 | 7.67 | Week 1 |  |  |
| Dawes | 2024-01-03 | 3 | -0.56 | 4.00 | -5.06 | 10.83 | 11.39 | Week 1 |  |  |
| Dawes | 2024-01-04 | 4 | -0.61 | 3.94 | -5.17 | 14.44 | 15.06 | Week 1 |  |  |
| Dawes | 2024-01-05 | 5 | -0.72 | 3.83 | -5.28 | 7.78 | 8.50 | Week 1 |  |  |
| Dawes | 2024-01-06 | 6 | -0.78 | 3.78 | -5.39 | 3.33 | 4.11 | Week 1 |  |  |
| Dawes | 2024-01-07 | 7 | -0.89 | 3.72 | -5.50 | 0.83 | 1.72 | **Week 1** | **4.834** | **<0.001** |
| Dawes | 2024-01-08 | 8 | -0.94 | 3.72 | -5.61 | -1.11 | -0.17 | Week 2 |  |  |
| Dawes | 2024-01-09 | 9 | -1.00 | 3.67 | -5.67 | 0.28 | 1.28 | Week 2 |  |  |
| Dawes | 2024-01-10 | 10 | -1.11 | 3.61 | -5.78 | -1.67 | -0.56 | Week 2 |  |  |
| Dawes | 2024-01-11 | 11 | -1.17 | 3.56 | -5.89 | 0.00 | 1.17 | Week 2 |  |  |
| Dawes | 2024-01-12 | 12 | -1.22 | 3.56 | -5.94 | 3.06 | 4.28 | Week 2 |  |  |
| Dawes | 2024-01-13 | 13 | -1.28 | 3.50 | -6.06 | 4.72 | 6.00 | Week 2 |  |  |
| Dawes | 2024-01-14 | 14 | -1.28 | 3.50 | -6.11 | -2.22 | -0.94 | Week 2 | 1.597 | 0.161 |
| Dawes | 2024-01-15 | 15 | -1.33 | 3.50 | -6.17 | -6.39 | -5.06 | Week 3 |  |  |
| Dawes | 2024-01-16 | 16 | -1.39 | 3.50 | -6.22 | -3.89 | -2.50 | Week 3 |  |  |
| Dawes | 2024-01-17 | 17 | -1.39 | 3.44 | -6.28 | 0.56 | 1.94 | Week 3 |  |  |
| Dawes | 2024-01-18 | 18 | -1.44 | 3.44 | -6.33 | 8.89 | 10.33 | Week 3 |  |  |
| Dawes | 2024-01-19 | 19 | -1.44 | 3.44 | -6.39 | 4.17 | 5.61 | Week 3 |  |  |
| Dawes | 2024-01-20 | 20 | -1.44 | 3.50 | -6.44 | 8.06 | 9.50 | Week 3 |  |  |
| Dawes | 2024-01-21 | 21 | -1.50 | 3.50 | -6.44 | 0.56 | 2.06 | Week 3 | 1.433 | 0.202 |
| Dawes | 2024-01-22 | 22 | -1.50 | 3.50 | -6.44 | -1.39 | 0.11 | Week 4 |  |  |
| Dawes | 2024-01-23 | 23 | -1.50 | 3.56 | -6.50 | -0.83 | 0.67 | Week 4 |  |  |
| Dawes | 2024-01-24 | 24 | -1.44 | 3.56 | -6.50 | -1.94 | -0.50 | Week 4 |  |  |
| Dawes | 2024-01-25 | 25 | -1.44 | 3.61 | -6.50 | 0.00 | 1.44 | Week 4 |  |  |
| Dawes | 2024-01-26 | 26 | -1.44 | 3.61 | -6.50 | 3.61 | 5.06 | Week 4 |  |  |
| Dawes | 2024-01-27 | 27 | -1.39 | 3.67 | -6.44 | -2.22 | -0.83 | Week 4 |  |  |
| Dawes | 2024-01-28 | 28 | -1.33 | 3.72 | -6.44 | 0.56 | 1.89 | Week 4 | 1.485 | 0.188 |
| Dawes | 2024-01-29 | 29 | -1.33 | 3.78 | -6.39 | 4.72 | 6.06 | Week 5 |  |  |
| Dawes | 2024-01-30 | 30 | -1.28 | 3.83 | -6.39 | 3.33 | 4.61 | Week 5 |  |  |
| Dawes | 2024-01-31 | 31 | -1.22 | 3.89 | -6.33 | -2.78 | -1.56 | Week 5 |  |  |
| Dawes | 2024-02-01 | 32 | -1.17 | 4.00 | -6.28 | -7.78 | -6.61 | Week 5 |  |  |
| Dawes | 2024-02-02 | 33 | -1.11 | 4.06 | -6.22 | -6.67 | -5.56 | Week 5 |  |  |
| Dawes | 2024-02-03 | 34 | -1.00 | 4.11 | -6.17 | -2.78 | -1.78 | Week 5 |  |  |
| Dawes | 2024-02-04 | 35 | -0.94 | 4.22 | -6.06 | -10.00 | -9.06 | Week 5 | -0.927 | 0.390 |
| Dawes | 2024-02-05 | 36 | -0.83 | 4.28 | -6.00 | -3.33 | -2.50 | Week 6 |  |  |
| Dawes | 2024-02-06 | 37 | -0.78 | 4.39 | -5.89 | 5.00 | 5.78 | Week 6 |  |  |
| Dawes | 2024-02-07 | 38 | -0.67 | 4.50 | -5.83 | -0.83 | -0.17 | Week 6 |  |  |
| Dawes | 2024-02-08 | 39 | -0.56 | 4.61 | -5.72 | 5.28 | 5.83 | Week 6 |  |  |
| Dawes | 2024-02-09 | 40 | -0.44 | 4.72 | -5.61 | 3.89 | 4.33 | Week 6 |  |  |
| Dawes | 2024-02-10 | 41 | -0.33 | 4.83 | -5.50 | 12.22 | 12.56 | Week 6 |  |  |
| Dawes | 2024-02-11 | 42 | -0.22 | 4.94 | -5.39 | -0.83 | -0.61 | Week 6 | 1.848 | 0.114 |
| Dawes | 2024-02-12 | 43 | -0.11 | 5.06 | -5.28 | -1.67 | -1.56 | Week 7 |  |  |
| Dawes | 2024-02-13 | 44 | 0.00 | 5.22 | -5.17 | 2.22 | 2.22 | Week 7 |  |  |
| Dawes | 2024-02-14 | 45 | 0.17 | 5.33 | -5.00 | 3.61 | 3.44 | Week 7 |  |  |
| Dawes | 2024-02-15 | 46 | 0.28 | 5.50 | -4.89 | 3.33 | 3.06 | Week 7 |  |  |
| Dawes | 2024-02-16 | 47 | 0.44 | 5.61 | -4.78 | 14.17 | 13.72 | Week 7 |  |  |
| Dawes | 2024-02-17 | 48 | 0.56 | 5.78 | -4.61 | 7.22 | 6.67 | Week 7 |  |  |
| Dawes | 2024-02-18 | 49 | 0.72 | 5.94 | -4.50 | -3.06 | -3.78 | Week 7 | 1.577 | 0.166 |
| Dawes | 2024-02-19 | 50 | 0.89 | 6.06 | -4.33 | -0.28 | -1.17 | Week 8 |  |  |
| Dawes | 2024-02-20 | 51 | 1.00 | 6.22 | -4.17 | 7.50 | 6.50 | Week 8 |  |  |
| Dawes | 2024-02-21 | 52 | 1.17 | 6.39 | -4.06 | 6.11 | 4.94 | Week 8 |  |  |
| Dawes | 2024-02-22 | 53 | 1.33 | 6.56 | -3.89 | 5.56 | 4.22 | Week 8 |  |  |
| Dawes | 2024-02-23 | 54 | 1.50 | 6.72 | -3.72 | 8.61 | 7.11 | Week 8 |  |  |
| Dawes | 2024-02-24 | 55 | 1.67 | 6.94 | -3.56 | 10.28 | 8.61 | Week 8 |  |  |
| Dawes | 2024-02-25 | 56 | 1.83 | 7.11 | -3.44 | 0.56 | -1.28 | **Week 8** | **2.785** | **0.032** |
| Dawes | 2024-02-26 | 57 | 2.00 | 7.28 | -3.28 | 2.50 | 0.50 | Week 9 |  |  |
| Dawes | 2024-02-27 | 58 | 2.17 | 7.50 | -3.11 | 3.61 | 1.44 | Week 9 |  |  |
| Dawes | 2024-02-28 | 59 | 2.33 | 7.67 | -2.94 | 8.33 | 6.00 | Week 9 |  |  |
| Dawes | 2024-03-01 | 60 | 2.56 | 7.89 | -2.78 | 2.78 | 0.22 | Week 9 |  |  |
| Dawes | 2024-03-02 | 61 | 2.72 | 8.06 | -2.67 | 11.11 | 8.39 | Week 9 |  |  |
| Dawes | 2024-03-03 | 62 | 2.89 | 8.28 | -2.50 | 5.56 | 2.67 | Week 9 |  |  |
| Dawes | 2024-03-04 | 63 | 3.06 | 8.50 | -2.33 | 6.94 | 3.89 | **Week 9** | **2.893** | **0.028** |
| Dawes | 2024-03-05 | 64 | 3.28 | 8.67 | -2.17 | 4.72 | 1.44 | Week 10 |  |  |
| Dawes | 2024-03-06 | 65 | 3.44 | 8.89 | -2.00 | 5.28 | 1.83 | Week 10 |  |  |
| Dawes | 2024-03-07 | 66 | 3.61 | 9.11 | -1.89 | 11.39 | 7.78 | Week 10 |  |  |
| Dawes | 2024-03-08 | 67 | 3.83 | 9.33 | -1.72 | 1.39 | -2.44 | Week 10 |  |  |
| Dawes | 2024-03-09 | 68 | 4.00 | 9.56 | -1.56 | 1.94 | -2.06 | Week 10 |  |  |
| Dawes | 2024-03-10 | 69 | 4.22 | 9.78 | -1.39 | 3.06 | -1.17 | Week 10 |  |  |
| Dawes | 2024-03-11 | 70 | 4.39 | 10.00 | -1.22 | 2.22 | -2.17 | Week 10 | 0.332 | 0.751 |
| Dawes | 2024-03-12 | 71 | 4.56 | 10.22 | -1.11 | -0.28 | -4.83 | Week 11 |  |  |
| Dawes | 2024-03-13 | 72 | 4.78 | 10.50 | -0.94 | 0.00 | -4.78 | Week 11 |  |  |
| Dawes | 2024-03-14 | 73 | 5.00 | 10.72 | -0.78 | -1.67 | -6.67 | Week 11 |  |  |
| Dawes | 2024-03-15 | 74 | 5.17 | 10.94 | -0.61 | -3.33 | -8.50 | Week 11 |  |  |
| Dawes | 2024-03-16 | 75 | 5.39 | 11.22 | -0.44 | 1.11 | -4.28 | Week 11 |  |  |
| Dawes | 2024-03-17 | 76 | 5.56 | 11.44 | -0.28 | 5.56 | 0.00 | Week 11 |  |  |
| Dawes | 2024-03-18 | 77 | 5.78 | 11.67 | -0.17 | 3.33 | -2.44 | **Week 11** | **-4.328** | **0.005** |
| Dawes | 2024-03-19 | 78 | 5.94 | 11.94 | 0.00 | -3.61 | -9.56 | Week 12 |  |  |
| Dawes | 2024-03-20 | 79 | 6.17 | 12.17 | 0.17 | -3.06 | -9.22 | Week 12 |  |  |
| Dawes | 2024-03-21 | 80 | 6.39 | 12.44 | 0.33 | 1.39 | -5.00 | Week 12 |  |  |
| Dawes | 2024-03-22 | 81 | 6.61 | 12.67 | 0.50 | 4.72 | -1.89 | Week 12 |  |  |
| Dawes | 2024-03-23 | 82 | 6.78 | 12.94 | 0.67 | 8.61 | 1.83 | Week 12 |  |  |
| Dawes | 2024-03-24 | 83 | 7.00 | 13.17 | 0.83 | 10.83 | 3.83 | Week 12 |  |  |
| Dawes | 2024-03-25 | 84 | 7.22 | 13.44 | 0.94 | 5.00 | -2.22 | Week 12 | -1.640 | 0.152 |
| Dawes | 2024-03-26 | 85 | 7.39 | 13.67 | 1.11 | 8.33 | 0.94 | Week 13 |  |  |
| Dawes | 2024-03-27 | 86 | 7.61 | 13.94 | 1.28 | 6.11 | -1.50 | Week 13 |  |  |
| Dawes | 2024-03-28 | 87 | 7.83 | 14.22 | 1.44 | 6.67 | -1.17 | Week 13 |  |  |
| Dawes | 2024-03-29 | 88 | 8.06 | 14.44 | 1.61 | 3.33 | -4.72 | Week 13 |  |  |
| Dawes | 2024-03-30 | 89 | 8.28 | 14.72 | 1.83 | 3.33 | -4.94 | Week 13 |  |  |
| Dawes | 2024-03-31 | 90 | 8.44 | 14.94 | 2.00 | 3.61 | -4.83 | Week 13 |  |  |
| Dawes | 2024-04-01 | 91 | 8.67 | 15.22 | 2.17 | 9.72 | 1.06 | Week 13 | -2.145 | 0.076 |
| Dawes | 2024-04-02 | 92 | 8.89 | 15.44 | 2.33 | 5.00 | -3.89 | Week 14 |  |  |
| Dawes | 2024-04-03 | 93 | 9.11 | 15.72 | 2.50 | 3.06 | -6.06 | Week 14 |  |  |
| Dawes | 2024-04-04 | 94 | 9.33 | 15.94 | 2.67 | 9.72 | 0.39 | Week 14 |  |  |
| Dawes | 2024-04-05 | 95 | 9.56 | 16.22 | 2.89 | 15.00 | 5.44 | Week 14 |  |  |
| Dawes | 2024-04-06 | 96 | 9.72 | 16.44 | 3.06 | 5.00 | -4.72 | Week 14 |  |  |
| Dawes | 2024-04-07 | 97 | 9.94 | 16.67 | 3.22 | 7.50 | -2.44 | Week 14 |  |  |
| Dawes | 2024-04-08 | 98 | 10.17 | 16.94 | 3.44 | 5.00 | -5.17 | Week 14 | -1.539 | 0.175 |
| Dawes | 2024-04-09 | 99 | 10.39 | 17.17 | 3.61 | 5.83 | -4.56 | Week 15 |  |  |
| Dawes | 2024-04-10 | 100 | 10.61 | 17.39 | 3.78 | 6.94 | -3.67 | Week 15 |  |  |
| Dawes | 2024-04-11 | 101 | 10.83 | 17.61 | 4.00 | 8.89 | -1.94 | Week 15 |  |  |
| Dawes | 2024-04-12 | 102 | 11.00 | 17.83 | 4.17 | 11.39 | 0.39 | Week 15 |  |  |
| Dawes | 2024-04-13 | 103 | 11.22 | 18.06 | 4.39 | 15.28 | 4.06 | Week 15 |  |  |
| Dawes | 2024-04-14 | 104 | 11.44 | 18.28 | 4.56 | 15.83 | 4.39 | Week 15 |  |  |
| Dawes | 2024-04-15 | 105 | 11.61 | 18.50 | 4.78 | 16.11 | 4.50 | Week 15 | 0.305 | 0.771 |
| Dawes | 2024-04-16 | 106 | 11.83 | 18.72 | 4.94 | 15.56 | 3.72 | Week 16 |  |  |
| Dawes | 2024-04-17 | 107 | 12.06 | 18.94 | 5.17 | 15.00 | 2.94 | Week 16 |  |  |
| Dawes | 2024-04-18 | 108 | 12.28 | 19.17 | 5.33 | 6.39 | -5.89 | Week 16 |  |  |
| Dawes | 2024-04-19 | 109 | 12.44 | 19.33 | 5.56 | 10.56 | -1.89 | Week 16 |  |  |
| Dawes | 2024-04-20 | 110 | 12.67 | 19.56 | 5.72 | 20.56 | 7.89 | Week 16 |  |  |
| Dawes | 2024-04-21 | 111 | 12.83 | 19.72 | 5.94 | 16.94 | 4.11 | Week 16 |  |  |
| Dawes | 2024-04-22 | 112 | 13.06 | 19.94 | 6.17 | 10.28 | -2.78 | Week 16 | 0.639 | 0.547 |
| Dawes | 2024-04-23 | 113 | 13.22 | 20.11 | 6.33 | 8.06 | -5.17 | Week 17 |  |  |
| Dawes | 2024-04-24 | 114 | 13.44 | 20.33 | 6.56 | 4.17 | -9.28 | Week 17 |  |  |
| Dawes | 2024-04-25 | 115 | 13.61 | 20.50 | 6.72 | 2.78 | -10.83 | Week 17 |  |  |
| Dawes | 2024-04-26 | 116 | 13.83 | 20.67 | 6.94 | 5.28 | -8.56 | Week 17 |  |  |
| Dawes | 2024-04-27 | 117 | 14.00 | 20.83 | 7.17 | 6.39 | -7.61 | Week 17 |  |  |
| Dawes | 2024-04-28 | 118 | 14.17 | 21.00 | 7.33 | 8.61 | -5.56 | Week 17 |  |  |
| Dawes | 2024-04-29 | 119 | 14.39 | 21.22 | 7.56 | 13.33 | -1.06 | **Week 17** | **-5.591** | **0.001** |
| Dawes | 2024-04-30 | 120 | 14.56 | 21.39 | 7.72 | 10.83 | -3.72 | Week 18 |  |  |
| Dawes | 2024-05-01 | 121 | 14.72 | 21.50 | 7.94 | 5.83 | -8.89 | Week 18 |  |  |
| Dawes | 2024-05-02 | 122 | 14.89 | 21.67 | 8.11 | 5.28 | -9.61 | Week 18 |  |  |
| Dawes | 2024-05-03 | 123 | 15.11 | 21.83 | 8.33 | 4.44 | -10.67 | Week 18 |  |  |
| Dawes | 2024-05-04 | 124 | 15.28 | 22.00 | 8.50 | 6.39 | -8.89 | Week 18 |  |  |
| Dawes | 2024-05-05 | 125 | 15.44 | 22.17 | 8.72 | 8.89 | -6.56 | Week 18 |  |  |
| Dawes | 2024-05-06 | 126 | 15.61 | 22.33 | 8.89 | 11.11 | -4.50 | **Week 18** | **-7.504** | **<0.001** |
| Dawes | 2024-05-07 | 127 | 15.78 | 22.44 | 9.11 | 14.17 | -1.61 | Week 19 |  |  |
| Dawes | 2024-05-08 | 128 | 15.94 | 22.61 | 9.28 | 18.06 | 2.11 | Week 19 |  |  |
| Dawes | 2024-05-09 | 129 | 16.11 | 22.78 | 9.44 | 17.22 | 1.11 | Week 19 |  |  |
| Dawes | 2024-05-10 | 130 | 16.28 | 22.94 | 9.67 | 12.50 | -3.78 | Week 19 |  |  |
| Dawes | 2024-05-11 | 131 | 16.44 | 23.06 | 9.83 | 13.89 | -2.56 | Week 19 |  |  |
| Dawes | 2024-05-12 | 132 | 16.61 | 23.22 | 10.06 | 16.94 | 0.33 | Week 19 |  |  |
| Dawes | 2024-05-13 | 133 | 16.78 | 23.39 | 10.22 | 21.11 | 4.33 | Week 19 | -0.007 | 0.994 |
| Dawes | 2024-05-14 | 134 | 16.94 | 23.50 | 10.39 | 20.00 | 3.06 | Week 20 |  |  |
| Dawes | 2024-05-15 | 135 | 17.11 | 23.67 | 10.56 | 17.22 | 0.11 | Week 20 |  |  |
| Dawes | 2024-05-16 | 136 | 17.28 | 23.83 | 10.78 | 16.11 | -1.17 | Week 20 |  |  |
| Dawes | 2024-05-17 | 137 | 17.44 | 23.94 | 10.94 | 13.89 | -3.56 | Week 20 |  |  |
| Dawes | 2024-05-18 | 138 | 17.61 | 24.11 | 11.11 | 10.00 | -7.61 | Week 20 |  |  |
| Dawes | 2024-05-19 | 139 | 17.78 | 24.28 | 11.28 | 21.39 | 3.61 | Week 20 |  |  |
| Dawes | 2024-05-20 | 140 | 17.94 | 24.44 | 11.50 | 16.67 | -1.28 | Week 20 | -0.670 | 0.528 |
| Dawes | 2024-05-21 | 141 | 18.11 | 24.56 | 11.67 | 16.11 | -2.00 | Week 21 |  |  |
| Dawes | 2024-05-22 | 142 | 18.28 | 24.72 | 11.83 | 18.61 | 0.33 | Week 21 |  |  |
| Dawes | 2024-05-23 | 143 | 18.44 | 24.89 | 12.00 | 20.56 | 2.11 | Week 21 |  |  |
| Dawes | 2024-05-24 | 144 | 18.61 | 25.06 | 12.17 | 20.56 | 1.94 | Week 21 |  |  |
| Dawes | 2024-05-25 | 145 | 18.78 | 25.22 | 12.33 | 14.72 | -4.06 | Week 21 |  |  |
| Dawes | 2024-05-26 | 146 | 18.94 | 25.39 | 12.50 | 12.22 | -6.72 | Week 21 |  |  |
| Dawes | 2024-05-27 | 147 | 19.11 | 25.50 | 12.67 | 14.17 | -4.94 | Week 21 | -1.444 | 0.199 |
| Dawes | 2024-05-28 | 148 | 19.28 | 25.67 | 12.89 | 15.28 | -4.00 | Week 22 |  |  |
| Dawes | 2024-05-29 | 149 | 19.44 | 25.83 | 13.06 | 17.50 | -1.94 | Week 22 |  |  |
| Dawes | 2024-05-30 | 150 | 19.61 | 26.00 | 13.22 | 20.56 | 0.94 | Week 22 |  |  |
| Dawes | 2024-05-31 | 151 | 19.78 | 26.17 | 13.39 | 20.83 | 1.06 | Week 22 |  |  |
| Dawes | 2024-06-01 | 152 | 19.94 | 26.33 | 13.56 | 20.28 | 0.33 | Week 22 |  |  |
| Dawes | 2024-06-02 | 153 | 20.11 | 26.50 | 13.67 | 20.83 | 0.72 | Week 22 |  |  |
| Dawes | 2024-06-03 | 154 | 20.28 | 26.67 | 13.83 | 21.94 | 1.67 | Week 22 | -0.226 | 0.828 |
| Dawes | 2024-06-04 | 155 | 20.39 | 26.83 | 14.00 | 22.78 | 2.39 | Week 23 |  |  |
| Dawes | 2024-06-05 | 156 | 20.56 | 27.00 | 14.17 | 20.00 | -0.56 | Week 23 |  |  |
| Dawes | 2024-06-06 | 157 | 20.72 | 27.11 | 14.33 | 19.94 | -0.78 | Week 23 |  |  |
| Dawes | 2024-06-07 | 158 | 20.89 | 27.28 | 14.50 | 17.78 | -3.11 | Week 23 |  |  |
| Dawes | 2024-06-08 | 159 | 21.06 | 27.44 | 14.61 | 17.33 | -3.72 | Week 23 |  |  |
| Dawes | 2024-06-09 | 160 | 21.17 | 27.61 | 14.78 | 19.44 | -1.72 | Week 23 |  |  |
| Dawes | 2024-06-10 | 161 | 21.33 | 27.78 | 14.94 | 21.72 | 0.39 | Week 23 | -1.290 | 0.245 |
| Dawes | 2024-06-11 | 162 | 21.50 | 27.89 | 15.06 | 19.33 | -2.17 | Week 24 |  |  |
| Dawes | 2024-06-12 | 163 | 21.61 | 28.06 | 15.22 | 16.78 | -4.83 | Week 24 |  |  |
| Dawes | 2024-06-13 | 164 | 21.78 | 28.22 | 15.33 | 13.83 | -7.94 | Week 24 |  |  |
| Dawes | 2024-06-14 | 165 | 21.89 | 28.33 | 15.50 | 17.22 | -4.67 | Week 24 |  |  |
| Dawes | 2024-06-15 | 166 | 22.06 | 28.50 | 15.61 | 19.39 | -2.67 | Week 24 |  |  |
| Dawes | 2024-06-16 | 167 | 22.17 | 28.61 | 15.78 | 17.28 | -4.89 | Week 24 |  |  |
| Dawes | 2024-06-17 | 168 | 22.28 | 28.72 | 15.89 | 19.06 | -3.22 | **Week 24** | **-5.942** | **0.001** |
| Dawes | 2024-06-18 | 169 | 22.44 | 28.83 | 16.00 | 21.61 | -0.83 | Week 25 |  |  |
| Dawes | 2024-06-19 | 170 | 22.56 | 29.00 | 16.11 | 21.72 | -0.83 | Week 25 |  |  |
| Dawes | 2024-06-20 | 171 | 22.67 | 29.11 | 16.22 | 20.56 | -2.11 | Week 25 |  |  |
| Dawes | 2024-06-21 | 172 | 22.78 | 29.22 | 16.33 | 23.06 | 0.28 | Week 25 |  |  |
| Dawes | 2024-06-22 | 173 | 22.89 | 29.28 | 16.44 | 19.50 | -3.39 | Week 25 |  |  |
| Dawes | 2024-06-23 | 174 | 22.94 | 29.39 | 16.56 | 19.94 | -3.00 | Week 25 |  |  |
| Dawes | 2024-06-24 | 175 | 23.06 | 29.50 | 16.67 | 22.28 | -0.78 | **Week 25** | **-3.011** | **0.024** |
| Dawes | 2024-06-25 | 176 | 23.17 | 29.56 | 16.72 | 23.94 | 0.78 | Week 26 |  |  |
| Dawes | 2024-06-26 | 177 | 23.22 | 29.67 | 16.83 | 22.61 | -0.61 | Week 26 |  |  |
| Dawes | 2024-06-27 | 178 | 23.33 | 29.72 | 16.89 | 19.56 | -3.78 | Week 26 |  |  |
| Dawes | 2024-06-28 | 179 | 23.39 | 29.78 | 17.00 | 20.56 | -2.83 | Week 26 |  |  |
| Dawes | 2024-06-29 | 180 | 23.44 | 29.83 | 17.06 | 24.00 | 0.56 | Week 26 |  |  |
| Dawes | 2024-06-30 | 181 | 23.50 | 29.89 | 17.11 | 25.61 | 2.11 | Week 26 |  |  |
| Dawes | 2024-07-01 | 182 | 23.56 | 29.94 | 17.17 | 25.11 | 1.56 | Week 26 | -0.377 | 0.719 |
| Dawes | 2024-07-02 | 183 | 23.61 | 30.00 | 17.22 | 22.94 | -0.67 | Week 27 |  |  |
| Dawes | 2024-07-03 | 184 | 23.67 | 30.06 | 17.28 | 22.33 | -1.33 | Week 27 |  |  |
| Dawes | 2024-07-04 | 185 | 23.72 | 30.06 | 17.33 | 25.28 | 1.56 | Week 27 |  |  |
| Dawes | 2024-07-05 | 186 | 23.72 | 30.11 | 17.39 | 26.72 | 3.00 | Week 27 |  |  |
| Dawes | 2024-07-06 | 187 | 23.78 | 30.11 | 17.44 | 24.33 | 0.56 | Week 27 |  |  |
| Dawes | 2024-07-07 | 188 | 23.78 | 30.17 | 17.44 | 24.61 | 0.83 | Week 27 |  |  |
| Dawes | 2024-07-08 | 189 | 23.83 | 30.17 | 17.50 | 23.22 | -0.61 | Week 27 | 0.842 | 0.432 |
| Dawes | 2024-07-09 | 190 | 23.83 | 30.17 | 17.50 | 22.50 | -1.33 | Week 28 |  |  |
| Dawes | 2024-07-10 | 191 | 23.83 | 30.17 | 17.56 | 22.72 | -1.11 | Week 28 |  |  |
| Dawes | 2024-07-11 | 192 | 23.89 | 30.17 | 17.56 | 24.33 | 0.44 | Week 28 |  |  |
| Dawes | 2024-07-12 | 193 | 23.89 | 30.17 | 17.56 | 25.78 | 1.89 | Week 28 |  |  |
| Dawes | 2024-07-13 | 194 | 23.89 | 30.17 | 17.56 | 26.78 | 2.89 | Week 28 |  |  |
| Dawes | 2024-07-14 | 195 | 23.89 | 30.11 | 17.61 | 26.39 | 2.50 | Week 28 |  |  |
| Dawes | 2024-07-15 | 196 | 23.83 | 30.11 | 17.61 | 23.50 | -0.33 | Week 28 | 1.079 | 0.322 |
| Dawes | 2024-07-16 | 197 | 23.83 | 30.11 | 17.61 | 24.06 | 0.22 | Week 29 |  |  |
| Dawes | 2024-07-17 | 198 | 23.83 | 30.06 | 17.56 | 24.11 | 0.28 | Week 29 |  |  |
| Dawes | 2024-07-18 | 199 | 23.83 | 30.06 | 17.56 | 21.89 | -1.94 | Week 29 |  |  |
| Dawes | 2024-07-19 | 200 | 23.78 | 30.06 | 17.56 | 24.17 | 0.39 | Week 29 |  |  |
| Dawes | 2024-07-20 | 201 | 23.78 | 30.00 | 17.56 | 24.50 | 0.72 | Week 29 |  |  |
| Dawes | 2024-07-21 | 202 | 23.78 | 30.00 | 17.50 | 21.67 | -2.11 | Week 29 |  |  |
| Dawes | 2024-07-22 | 203 | 23.72 | 29.94 | 17.50 | 22.44 | -1.28 | Week 29 | -1.169 | 0.287 |
| Dawes | 2024-07-23 | 204 | 23.72 | 29.89 | 17.50 | 22.39 | -1.33 | Week 30 |  |  |
| Dawes | 2024-07-24 | 205 | 23.67 | 29.89 | 17.44 | 22.39 | -1.28 | Week 30 |  |  |
| Dawes | 2024-07-25 | 206 | 23.61 | 29.83 | 17.44 | 22.67 | -0.94 | Week 30 |  |  |
| Dawes | 2024-07-26 | 207 | 23.61 | 29.83 | 17.39 | 26.44 | 2.83 | Week 30 |  |  |
| Dawes | 2024-07-27 | 208 | 23.56 | 29.78 | 17.33 | 27.33 | 3.78 | Week 30 |  |  |
| Dawes | 2024-07-28 | 209 | 23.56 | 29.78 | 17.33 | 24.83 | 1.28 | Week 30 |  |  |
| Dawes | 2024-07-29 | 210 | 23.50 | 29.72 | 17.28 | 24.06 | 0.56 | Week 30 | 0.903 | 0.401 |
| Dawes | 2024-07-30 | 211 | 23.44 | 29.72 | 17.22 | 23.61 | 0.17 | Week 31 |  |  |
| Dawes | 2024-07-31 | 212 | 23.44 | 29.67 | 17.22 | 21.94 | -1.50 | Week 31 |  |  |
| Dawes | 2024-08-01 | 213 | 23.39 | 29.61 | 17.17 | 21.44 | -1.94 | Week 31 |  |  |
| Dawes | 2024-08-02 | 214 | 23.33 | 29.61 | 17.11 | 22.33 | -1.00 | Week 31 |  |  |
| Dawes | 2024-08-03 | 215 | 23.33 | 29.56 | 17.06 | 24.33 | 1.00 | Week 31 |  |  |
| Dawes | 2024-08-04 | 216 | 23.28 | 29.56 | 17.00 | 25.11 | 1.83 | Week 31 |  |  |
| Dawes | 2024-08-05 | 217 | 23.22 | 29.50 | 16.94 | 24.00 | 0.78 | Week 31 | -0.178 | 0.864 |
| Dawes | 2024-08-06 | 218 | 23.17 | 29.50 | 16.89 | 25.28 | 2.11 | Week 32 |  |  |
| Dawes | 2024-08-07 | 219 | 23.17 | 29.44 | 16.83 | 21.94 | -1.22 | Week 32 |  |  |
| Dawes | 2024-08-08 | 220 | 23.11 | 29.44 | 16.78 | 22.39 | -0.72 | Week 32 |  |  |
| Dawes | 2024-08-09 | 221 | 23.06 | 29.39 | 16.72 | 22.44 | -0.61 | Week 32 |  |  |
| Dawes | 2024-08-10 | 222 | 23.00 | 29.39 | 16.61 | 21.78 | -1.22 | Week 32 |  |  |
| Dawes | 2024-08-11 | 223 | 22.94 | 29.33 | 16.56 | 23.56 | 0.61 | Week 32 |  |  |
| Dawes | 2024-08-12 | 224 | 22.89 | 29.33 | 16.50 | 25.78 | 2.89 | Week 32 | 0.417 | 0.691 |
| Dawes | 2024-08-13 | 225 | 22.83 | 29.28 | 16.44 | 24.17 | 1.33 | Week 33 |  |  |
| Dawes | 2024-08-14 | 226 | 22.78 | 29.28 | 16.33 | 22.06 | -0.72 | Week 33 |  |  |
| Dawes | 2024-08-15 | 227 | 22.72 | 29.22 | 16.28 | 20.28 | -2.44 | Week 33 |  |  |
| Dawes | 2024-08-16 | 228 | 22.67 | 29.17 | 16.17 | 20.94 | -1.72 | Week 33 |  |  |
| Dawes | 2024-08-17 | 229 | 22.61 | 29.17 | 16.11 | 20.83 | -1.78 | Week 33 |  |  |
| Dawes | 2024-08-18 | 230 | 22.56 | 29.11 | 16.00 | 19.50 | -3.06 | Week 33 |  |  |
| Dawes | 2024-08-19 | 231 | 22.50 | 29.06 | 15.89 | 19.61 | -2.89 | **Week 33** | **-2.799** | **0.031** |
| Dawes | 2024-08-20 | 232 | 22.44 | 29.06 | 15.83 | 24.17 | 1.72 | Week 34 |  |  |
| Dawes | 2024-08-21 | 233 | 22.33 | 29.00 | 15.72 | 27.28 | 4.94 | Week 34 |  |  |
| Dawes | 2024-08-22 | 234 | 22.28 | 28.94 | 15.61 | 23.67 | 1.39 | Week 34 |  |  |
| Dawes | 2024-08-23 | 235 | 22.17 | 28.89 | 15.50 | 25.17 | 3.00 | Week 34 |  |  |
| Dawes | 2024-08-24 | 236 | 22.11 | 28.83 | 15.39 | 23.89 | 1.78 | Week 34 |  |  |
| Dawes | 2024-08-25 | 237 | 22.00 | 28.78 | 15.28 | 26.22 | 4.22 | Week 34 |  |  |
| Dawes | 2024-08-26 | 238 | 21.94 | 28.72 | 15.17 | 22.89 | 0.94 | **Week 34** | **4.464** | **0.004** |
| Dawes | 2024-08-27 | 239 | 21.83 | 28.61 | 15.06 | 21.00 | -0.83 | Week 35 |  |  |
| Dawes | 2024-08-28 | 240 | 21.72 | 28.56 | 14.89 | 20.00 | -1.72 | Week 35 |  |  |
| Dawes | 2024-08-29 | 241 | 21.61 | 28.50 | 14.78 | 20.00 | -1.61 | Week 35 |  |  |
| Dawes | 2024-08-30 | 242 | 21.50 | 28.39 | 14.67 | 18.61 | -2.89 | Week 35 |  |  |
| Dawes | 2024-08-31 | 243 | 21.39 | 28.28 | 14.50 | 18.72 | -2.67 | Week 35 |  |  |
| Dawes | 2024-09-01 | 244 | 21.28 | 28.22 | 14.39 | 20.61 | -0.67 | Week 35 |  |  |
| Dawes | 2024-09-02 | 245 | 21.17 | 28.11 | 14.22 | 23.78 | 2.61 | Week 35 | -1.597 | 0.161 |
| Dawes | 2024-09-03 | 246 | 21.06 | 28.00 | 14.06 | 25.94 | 4.89 | Week 36 |  |  |
| Dawes | 2024-09-04 | 247 | 20.89 | 27.89 | 13.89 | 25.67 | 4.78 | Week 36 |  |  |
| Dawes | 2024-09-05 | 248 | 20.78 | 27.78 | 13.72 | 26.67 | 5.89 | Week 36 |  |  |
| Dawes | 2024-09-06 | 249 | 20.61 | 27.67 | 13.61 | 25.56 | 4.94 | Week 36 |  |  |
| Dawes | 2024-09-07 | 250 | 20.50 | 27.56 | 13.44 | 22.11 | 1.61 | Week 36 |  |  |
| Dawes | 2024-09-08 | 251 | 20.33 | 27.39 | 13.22 | 22.33 | 2.00 | Week 36 |  |  |
| Dawes | 2024-09-09 | 252 | 20.17 | 27.28 | 13.06 | 19.94 | -0.22 | **Week 36** | **3.974** | **0.007** |
| Dawes | 2024-09-10 | 253 | 20.00 | 27.11 | 12.89 | 19.78 | -0.22 | Week 37 |  |  |
| Dawes | 2024-09-11 | 254 | 19.83 | 26.94 | 12.72 | 19.56 | -0.28 | Week 37 |  |  |
| Dawes | 2024-09-12 | 255 | 19.67 | 26.83 | 12.56 | 19.06 | -0.61 | Week 37 |  |  |
| Dawes | 2024-09-13 | 256 | 19.50 | 26.67 | 12.33 | 17.00 | -2.50 | Week 37 |  |  |
| Dawes | 2024-09-14 | 257 | 19.33 | 26.50 | 12.17 | 16.72 | -2.61 | Week 37 |  |  |
| Dawes | 2024-09-15 | 258 | 19.17 | 26.33 | 11.94 | 16.89 | -2.28 | Week 37 |  |  |
| Dawes | 2024-09-16 | 259 | 18.94 | 26.17 | 11.78 | 16.94 | -2.00 | **Week 37** | **-3.673** | **0.010** |
| Dawes | 2024-09-17 | 260 | 18.78 | 25.94 | 11.56 | 18.28 | -0.50 | Week 38 |  |  |
| Dawes | 2024-09-18 | 261 | 18.56 | 25.78 | 11.39 | 17.17 | -1.39 | Week 38 |  |  |
| Dawes | 2024-09-19 | 262 | 18.39 | 25.61 | 11.17 | 16.56 | -1.83 | Week 38 |  |  |
| Dawes | 2024-09-20 | 263 | 18.17 | 25.39 | 10.94 | 20.00 | 1.83 | Week 38 |  |  |
| Dawes | 2024-09-21 | 264 | 18.00 | 25.22 | 10.78 | 20.83 | 2.83 | Week 38 |  |  |
| Dawes | 2024-09-22 | 265 | 17.78 | 25.00 | 10.56 | 20.44 | 2.67 | Week 38 |  |  |
| Dawes | 2024-09-23 | 266 | 17.56 | 24.78 | 10.33 | 18.00 | 0.44 | Week 38 | 0.803 | 0.453 |
| Dawes | 2024-09-24 | 267 | 17.39 | 24.61 | 10.17 | 18.22 | 0.83 | Week 39 |  |  |
| Dawes | 2024-09-25 | 268 | 17.17 | 24.39 | 9.94 | 17.83 | 0.67 | Week 39 |  |  |
| Dawes | 2024-09-26 | 269 | 16.94 | 24.17 | 9.72 | 19.28 | 2.33 | Week 39 |  |  |
| Dawes | 2024-09-27 | 270 | 16.72 | 23.94 | 9.50 | 18.67 | 1.94 | Week 39 |  |  |
| Dawes | 2024-09-28 | 271 | 16.50 | 23.72 | 9.28 | 18.33 | 1.83 | Week 39 |  |  |
| Dawes | 2024-09-29 | 272 | 16.28 | 23.50 | 9.11 | 19.11 | 2.83 | Week 39 |  |  |
| Dawes | 2024-09-30 | 273 | 16.06 | 23.28 | 8.89 | 19.89 | 3.83 | **Week 39** | **4.884** | **0.003** |
| Dawes | 2024-10-01 | 274 | 15.89 | 23.06 | 8.67 | 20.89 | 5.00 | Week 40 |  |  |
| Dawes | 2024-10-02 | 275 | 15.67 | 22.83 | 8.44 | 21.89 | 6.22 | Week 40 |  |  |
| Dawes | 2024-10-03 | 276 | 15.44 | 22.61 | 8.22 | 22.78 | 7.33 | Week 40 |  |  |
| Dawes | 2024-10-04 | 277 | 15.22 | 22.39 | 8.06 | 22.72 | 7.50 | Week 40 |  |  |
| Dawes | 2024-10-05 | 278 | 15.00 | 22.17 | 7.83 | 20.28 | 5.28 | Week 40 |  |  |
| Dawes | 2024-10-06 | 279 | 14.78 | 21.89 | 7.61 | 18.06 | 3.28 | Week 40 |  |  |
| Dawes | 2024-10-07 | 280 | 14.56 | 21.67 | 7.39 | 10.06 | -4.50 | **Week 40** | **2.746** | **0.033** |
| Dawes | 2024-10-08 | 281 | 14.33 | 21.44 | 7.22 | 9.33 | -5.00 | Week 41 |  |  |
| Dawes | 2024-10-09 | 282 | 14.11 | 21.22 | 7.00 | 11.50 | -2.61 | Week 41 |  |  |
| Dawes | 2024-10-10 | 283 | 13.89 | 20.94 | 6.78 | 12.11 | -1.78 | Week 41 |  |  |
| Dawes | 2024-10-11 | 284 | 13.67 | 20.72 | 6.61 | 13.00 | -0.67 | Week 41 |  |  |
| Dawes | 2024-10-12 | 285 | 13.44 | 20.50 | 6.39 | 16.33 | 2.89 | Week 41 |  |  |
| Dawes | 2024-10-13 | 286 | 13.22 | 20.22 | 6.17 | 16.28 | 3.06 | Week 41 |  |  |
| Dawes | 2024-10-14 | 287 | 13.00 | 20.00 | 6.00 | 14.44 | 1.44 | Week 41 | -0.335 | 0.749 |
| Dawes | 2024-10-15 | 288 | 12.78 | 19.78 | 5.78 | 10.33 | -2.44 | Week 42 |  |  |
| Dawes | 2024-10-16 | 289 | 12.56 | 19.50 | 5.61 | 10.50 | -2.06 | Week 42 |  |  |
| Dawes | 2024-10-17 | 290 | 12.33 | 19.28 | 5.39 | 11.33 | -1.00 | Week 42 |  |  |
| Dawes | 2024-10-18 | 291 | 12.11 | 19.06 | 5.22 | 10.89 | -1.22 | Week 42 |  |  |
| Dawes | 2024-10-19 | 292 | 11.89 | 18.78 | 5.00 | 13.33 | 1.44 | Week 42 |  |  |
| Dawes | 2024-10-20 | 293 | 11.72 | 18.56 | 4.83 | 12.33 | 0.61 | Week 42 |  |  |
| Dawes | 2024-10-21 | 294 | 11.50 | 18.33 | 4.67 | 10.94 | -0.56 | Week 42 | -1.422 | 0.205 |
| Dawes | 2024-10-22 | 295 | 11.28 | 18.06 | 4.44 | 9.39 | -1.89 | Week 43 |  |  |
| Dawes | 2024-10-23 | 296 | 11.06 | 17.83 | 4.28 | 9.17 | -1.89 | Week 43 |  |  |
| Dawes | 2024-10-24 | 297 | 10.83 | 17.56 | 4.11 | 16.00 | 5.17 | Week 43 |  |  |
| Dawes | 2024-10-25 | 298 | 10.61 | 17.33 | 3.94 | 18.78 | 8.17 | Week 43 |  |  |
| Dawes | 2024-10-26 | 299 | 10.39 | 17.06 | 3.72 | 18.56 | 8.17 | Week 43 |  |  |
| Dawes | 2024-10-27 | 300 | 10.22 | 16.83 | 3.56 | 18.56 | 8.33 | Week 43 |  |  |
| Dawes | 2024-10-28 | 301 | 10.00 | 16.61 | 3.39 | 14.56 | 4.56 | **Week 43** | **2.550** | **0.044** |
| Dawes | 2024-10-29 | 302 | 9.78 | 16.33 | 3.22 | 12.28 | 2.50 | Week 44 |  |  |
| Dawes | 2024-10-30 | 303 | 9.56 | 16.11 | 3.06 | 7.44 | -2.11 | Week 44 |  |  |
| Dawes | 2024-10-31 | 304 | 9.39 | 15.83 | 2.89 | 2.39 | -7.00 | Week 44 | -0.803 | 0.506 |

**Table 1:** Data table containing 30-year historic daily temperature normals (ºC), 30-year historic daily maximum temperatures (ºC), 30-year historic daily minimum temperatures (ºC), mean daily temperatures in 2023 (ºC), deviation in 2023 mean daily temperatures and 30-year normals; garden location, date, Julian day, and week of the year, along with test statistics and *p*-values from one-sample t-tests applied to the deviations for each week. Garden/weeks yielding significant *p*-values are indicated with bold font.


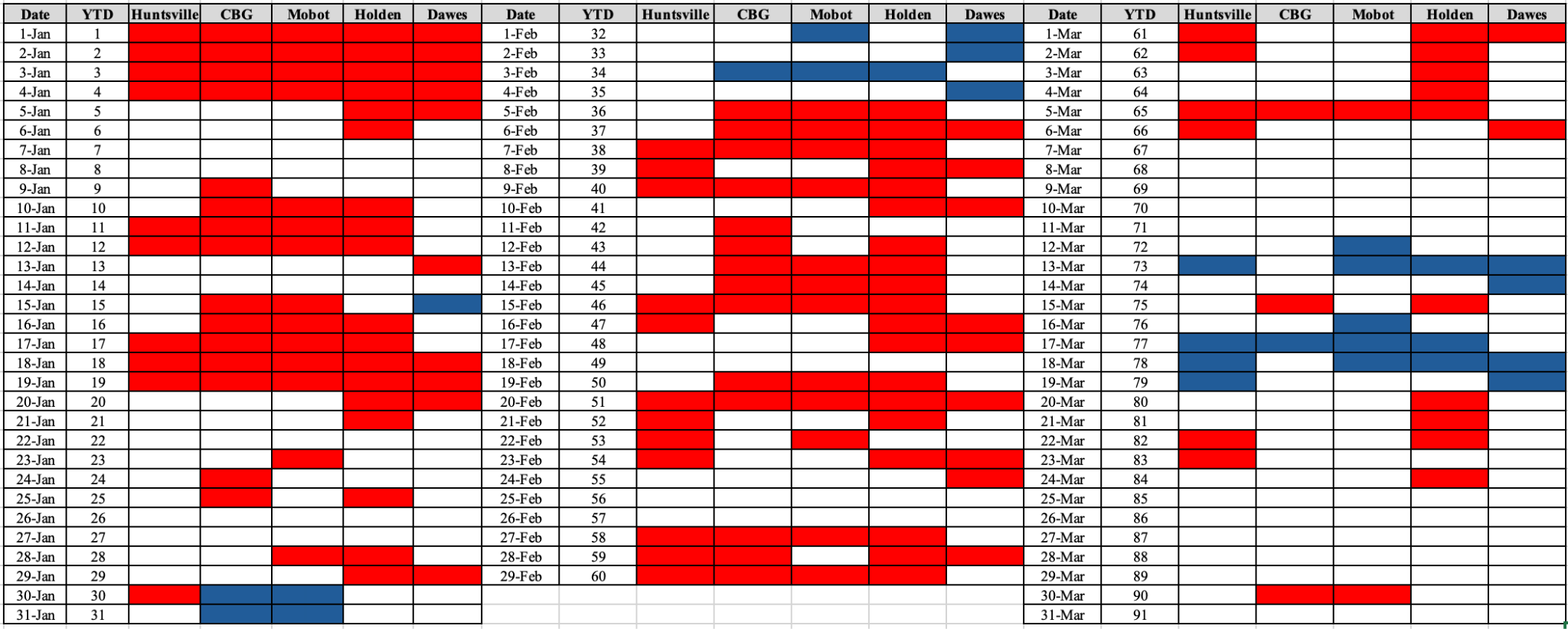


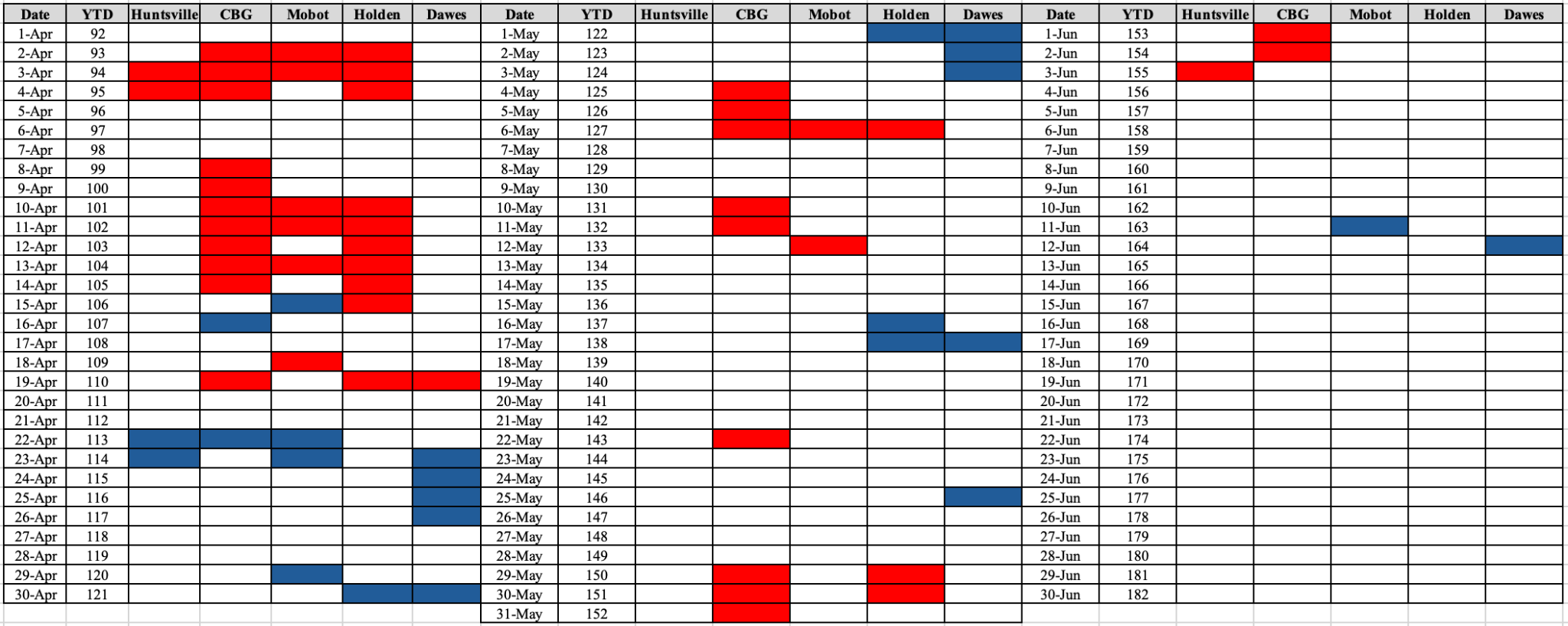


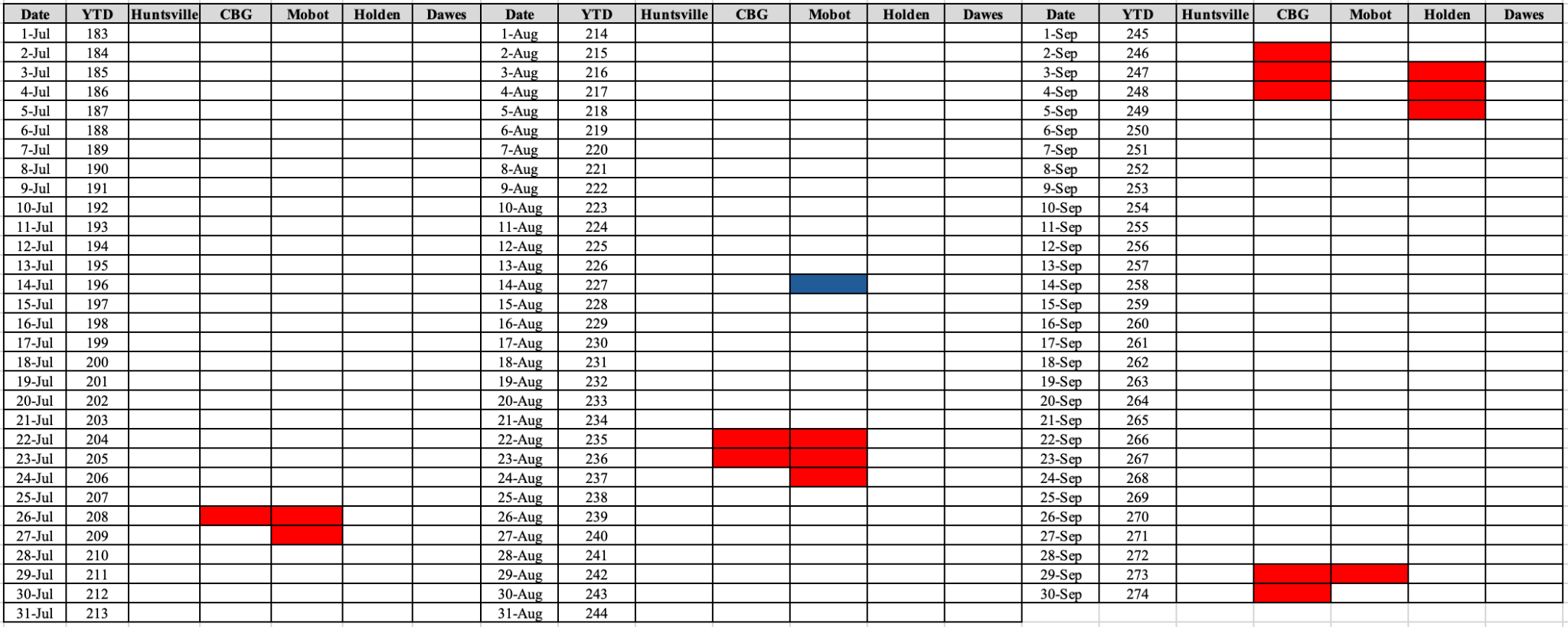


**
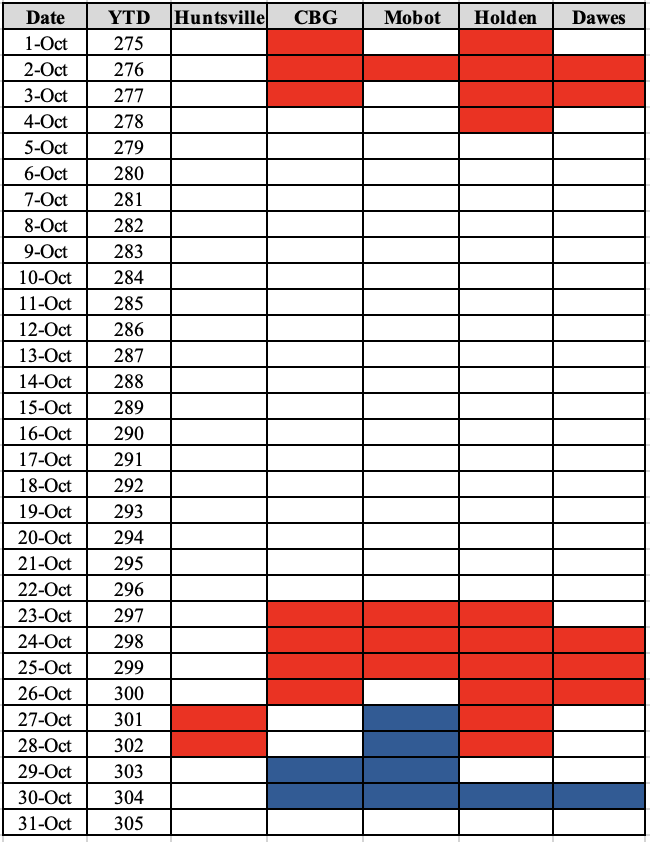
**

**Table 2:** Days in 2023 with mean temperatures that exceed (red) or were below (blue) the mean 30-year (1991-2020) maximum or minimum temperature, respectively, at the five garden locations. Total number of days in 2023 that fall outside the maximum/minimum historic temperature range are as follows: Huntsville - 39; CBG - 78; Mobot - 65; Holden - 82; Dawes - 47.

| **Garden** | **Mean fruiting duration** | **SD fruiting duration** | **Mean earliest date of fruiting** | **SD earliest date of fruiting** | **Mean flowering duration** | **SD flowering duration** | **Mean earliest date of flowering** | **SD earliest date of flowering** | **2023 variance from mean 30-yr daily temps** |
| --- | --- | --- | --- | --- | --- | --- | --- | --- | --- |
| **CBG** | 6.30 | 7.45 | 2023-05-19 | 13.08 | 23.60 | 19.12 | 2023-04-24 | 13.83 | 111.38 |
| **Holden** | 23.57 | 16.04 | 2023-05-09 | 15.85 | 36.04 | 13.80 | 2023-04-23 | 12.58 | 111.53 |
| **Dawes** | 43.82 | 11.81 | 2023-04-12 | 7.02 | 50.23 | 19.81 | 2023-04-09 | 15.27 | 85.22 |
| **Mobot** | 27.22 | 23.46 | 2023-04-30 | 22.73 | 46.28 | 22.75 | 2023-04-07 | 17.57 | 84.43 |
| **Huntsville** | 33.09 | 16.50 | 2023-04-13 | 21.68 | 50.93 | 25.11 | 2023-03-28 | 20.50 | 84.20 |

**Table 3:** Mean and standard deviation of flowering and fruiting deviation, as well as mean earliest dates of flowering and fruiting in 2023 for all study species at each garden location. Last column indicates the total variance from 30-year historic mean daily temperatures for 2023 average daily temperatures at each garden.
